# Supplementary material for: RELEASE-HF study: a protocol for an observational, registry-based study on the effectiveness of telemedicine in heart failure in the Netherlands
Source: BMJ Open. 2024 Jan 4;14(1):e078021. doi: 10.1136/bmjopen-2023-078021 (PMC10773380; doi:10.1136/bmjopen-2023-078021)
Supplement: Supplementary data [file bmjopen-2023-078021supp001.pdf]

Supplementary material 1 Variables of the heart failure registry

NOTE: Based on the dynamic data dictionary version 22.1.4 September 9, 2022. Definitions of variables and included variables are subject to change, since data dictionary depends on the current HF guidelines and is compiled by a committee consisting of delegated cardiologists from Dutch hospitals.

	Variables	Answer possibilities	Baseline (T0)	6 months (T1)	12 months (T2)
**Identifying variables**	Diagnosis number *	Unique number set up by the hospital	**X**		
	Date of diagnosis *	Date (DD-MM-YYYY)	**X**		
	Patient number*	Identification number in the hospital	**X**		
	Maiden name*	Name	**X**		
	Surname*	Name	**X**		
	Date of birth[$]	Date (DD-MM-YYYY)	**X**		
	Sex	Male<br>Female	**X**		
	Zip code*	1000 AA – 9999 ZZ= the Netherlands<br>0000= Abroad<br>-1= Unknown	**X**		
**Diagnostic variables**	Setting of inclusion	10= Outpatient clinic<br>20= Hospital, novo patient<br>30= Hospital, acute/chronic HF patient<br>-1 = Unknown	**X**		
	Previous HF diagnosis	0= No<br>1= Yes<br>-1= Unknown	**X**		
	Date of previous diagnosis (if applicable)	Date (DD-MM-YYYY)	**X**		
	Location diagnosis HF	10= Not earlier diagnosed with HF<br>10= Primary care<br>20= Secondary care<br>30= Tertiary care<br>-1= Unknown	**X**		
	Type of HF	10= HFrEF<br>20= HFmrEF<br>30= HFpEF<br>-1= Unknown	**X**		
	Etiology HFrEF	0= No HFrEF<br>10= Ischemic cardiomyopathy<br>20= Non-ischemic, hypertensive cardiomyopathy<br>50= Non-ischemic, arrhythmia/tachycardia mediated<br>60= Non-ischemic, hypertrophic cardiomyopathy<br>70= Non-ischemic, dilated cardiomyopathy (eci)<br>80= Non-ischemic, inflammation/infection (i.e., myocarditis)<br>90= Nonischemic, restrictive cardiomyopathy and/or accumulation<br>111= Nonischemic, familiar cq. genetic cardiomyopathy with proven DNA mutation	**X**		

van Eijk J, *et al. BMJ Open* 2024; 14:e078021. doi: 10.1136/bmjopen-2023-078021

		120= Non-ischemic, pericardial<br>135= Non-ischemic, toxic cardiomyopathy due to alcohol/drugs<br>136= Non-ischemic, toxic cardiomyopathy due to chemotherapy/radiation<br>139= Non-ischemic, toxic cardiomyopathy due to other<br>140= Non-ischemic, eci or (as yet) undetermined<br>900= Other (i.e., valve disease, metabolic, peripartum)<br>-1= Unknown			
	Etiology HFpEF	0= No HFpEF<br>10= Secondary HFpEF<br>20= Isolated hypertensive heart disease<br>30= Genetic HCM<br>40= Amyloidosis<br>50= Sarcoidosis<br>60= Hemochromatosis<br>70= Immune/inflammatory<br>80= Aortic valve stenosis<br>90= Pericardial diseases<br>900= Other<br>-1= Unknown	**X**		
**Patient characteristics**	***Identifying variables***				
	Follow-up moment	0 = T0<br>6 = T1<br>12 = T2<br>24 = T3<br>36 = T4<br>48 = T5	**X**	**X**	**X**
	Comorbidities				
	Hypertension	0= No<br>1= Yes<br>-1= Unknown	**X**	**X**	**X**
	Cardiovascular accident	0= No<br>1= Yes<br>-1= Unknown	**X**	**X**	**X**
	Extracardiac arterial vascular pathology	0= No<br>1= Yes<br>-1= Unknown	**X**	**X**	**X**
	Chronic respiratory disease	0= No<br>1= Yes<br>-1= Unknown	**X**	**X**	**X**
	Obstructive Sleep Apnea Syndrome	0= No<br>1= Yes<br>-1= Unknown	**X**	**X**	**X**
	Diabetes Mellitus	0= No<br>1= Diabetes, treatment unknown<br>2= Diabetes, no treatment<br>10= Diabetes, diet<br>20= Diabetes, oral medication<br>30= Diabetes, insulin<br>90= Diabetes, other<br>-1= Unknown	**X**	**X**	**X**
	Thyroid disorder	0= No<br>10= Yes, hypothyroidism<br>20= Yes, hyperthyroidism	**X**	**X**	**X**

		-1= Unknown			
	Malignancy	0= No<br>10= Yes, in the past<br>20= Yes, currently<br>-1= Unknown	X	X	X
	Measurements				
	Length	Measurement in centimeters	X	X	X
	Weight	Measurement in kilogram	X	X	X
	Systolic blood pressure	Measurement in mmHg	X	X	X
	Diastolic blood pressure	Measurement in mmHg	X	X	X
	Heart rhythm	10= Sinus rhythm<br>20= Atrium fibrillation<br>30= Atrial pacing<br>90= Other<br>-1= Unknown	X	X	X
	Heartrate	Measurement in beats per minute	X	X	X
	Atrial heart rhythm disorder	0= No<br>1= Yes<br>-1= Unknown	X	X	X
	Left bundle branch block	0= No<br>10= Yes, own heart's electrical conduction<br>20= Yes, pacing rhythm<br>-1= Unknown	X	X	X
	QRS complex duration	Measurement in milliseconds	X	X	X
	Left ventricle ejection fraction, NYHA classification and laboratory parameters				
	Left ventricle ejection fraction	Measurement in percentages	X	X	X
	NYHA class	1= Class I<br>2= Class II<br>3= Class III<br>4= Class IV<br>-1= Unknown	X	X	X
	NT-proBNP	1 t/m 8000= Value in pmol/l<br>0= No NT-proBNP measurement<br>-1= Unknown	X	X	X
	Date measurement NT-proBNP[$]	Date (DD-MM-YYYY)	X	X	X
	Serum creatinine	Value in µmol/l	X	X	X
	Iron deficiency	1= No<br>2= Yes, absolute ferritin < 100 µg/L<br>3= Yes, relative ferritin 100-300 µg/L with transferrin saturation < 20%<br>-1= Unknown	X	X	X
	Anemia	0= No<br>1= Yes<br>-1= Unknown	X	X	X
**Medication**	***Medication with target dose***				
	Type ACE inhibitor	0= No ACE inhibitor prescribed at Tn<br>10= Captopril (td in ESC 2021 = 150 mg)<br>20= Enalapril (td in ESC 2021= 20-40 mg)<br>3-= Lisinopril (td in ESC 2021 = 20-35 mg)<br>40= Ramipril (td in ESC 2021 = 10 mg)<br>50= Perindopril (td in ESC 2021 = 8 mg)	**(X)**	X	X

		60= Quinapril (td in ESC 2021 = 20 mg)<br>70= Fosinopril (td in ESC 2021 = 20-40 mg)<br>90= Other<br>-1= Unknown			
	Target dose ACE inhibitor	0= No ACE inhibitor prescribed at Tn<br>1= No, target dose in ESC 2021 not reached due to contraindication or otherwise<br>2= No, target dose in ESC 2021 not reached due to titration phase<br>10= Yes, target dose in ESC 2021 reached or maximally tolerable for this patient<br>-1= Unknown	**(X)**	**X**	**X**
	Type ARB	0= No ARB prescribed at Tn<br>10= Candesartan (td in ESC 2021 = 32 mg)<br>20= Losartan (td in ESC 2021 = 150 mg)<br>30= Valsartan (td in ESC 2021 = 320 mg)<br>40= Telmisartan (td in ESC 2021 = 80 mg)<br>50= Irbesartan (td in ESC 2021 = 300 mg)<br>90= Other<br>-1= Unknown	**(X)**	**X**	**X**
	Target dose ARB	0= No ARB prescribed at Tn<br>1= No, target dose in ESC 2021 not reached due to contraindication or otherwise<br>2= No, target dose in ESC 2021 not reached due to titration phase<br>10= Yes, target dose in ESC 2021 reached or maximally tolerable for this patient<br>-1= Unknown	**(X)**	**X**	**X**
	ARNI	0= No<br>1= Yes (td in ESC 2021 = 194-206 mg)<br>-1= Unknown	**(X)**	**X**	**X**
	Target dose ARNI	0= No ARNI prescribed at Tn<br>1= No, target dose in ESC 2021 not reached due to contraindication or otherwise<br>2= No, target dose in ESC 2021 not reached due to titration phase<br>10= Yes, target dose in ESC 2021 reached or maximally tolerable for this patient<br>-1= Unknown	**(X)**	**X**	**X**
	Type beta-blocker	0 = No beta blocker prescribed at Tn<br>10= Bisoprolol (td in ESC 2021 = 10 mg)<br>20= Metoprolol (td in ESC 2021 = 200 mg)<br>30= Carvedilol (td in ESC 2021 = 50 mg)	**(X)**	**X**	**X**

		40= Nebivolol (td in ESC 2021 = 10 mg)<br>50= Atenolol (td in ESC 2021 = 100 mg)<br>60= Pindolol (td in ESC 2021 = 20 mg)<br>70= Propranolol (td in ESC 2021 = 160 mg)<br>90= Other<br>-1= Unknown			
	Target dose beta-blocker	0= No beta blocker prescribed at Tn<br>1= No, target dose in ESC 2021 not reached due to contraindication or otherwise<br>2= No, target dose in ESC 2021 not reached due to titration phase<br>10= Yes, target dose in ESC 2021 reached or maximally tolerable for this patient<br>-1= Unknown	**(X)**	**X**	**X**
	Type MRA	0= No MRA prescribed at Tn<br>10= Eplerenone (td in ESC 2021= 50 mg)<br>20= Spironolactone (td in ESC 2021= 50 mg)<br>90= Other<br>-1= Unknown	**(X)**	**X**	**X**
	Target dose MRA	0= No MRA prescribed at Tn<br>1= No, target dose in ESC 2021 not reached due to contraindication or otherwise<br>2= No, target dose in ESC 2021 not reached due to titration phase<br>10= Yes, target dose in ESC 2021 reached or maximally tolerable for this patient<br>-1= Unknown	**(X)**	**X**	**X**
	Type SGLT2 inhibitor	0= No SGLT2 inhibitor prescribed at Tn<br>10= Canagliflozin (td in ESC 2021 = 10 mg)<br>20= Dapagliflozin (td in ESC 2021 = 10 mg)<br>30= Empagliflozin (td in ESC 2021 = 10 mg)<br>90= Other<br>-1= Unknown	**(X)**	**X**	**X**
	Target dose SGLT2 inhibitor	0= No SGLT2 inhibitor prescribed at Tn<br>1= No, target dose in ESC 2021 not reached due to contraindication or otherwise<br>2= No, target dose in ESC 2021 not reached due to titration phase<br>10= Yes, target dose in ESC 2021 reached or maximally tolerable for this patient<br>-1= Unknown	**(X)**	**X**	**X**
	Ivabradine	0= No<br>1= Yes	**(X)**	**X**	**X**

		-1= Unknown			
	Target dose Ivabradine	0= No ivabradine prescribed at Tn<br>1= No, target dose in ESC 2021 not reached due to contraindication or otherwise<br>2= No, target dose in ESC 2021 not reached due to titration phase<br>10= Yes, target dose in ESC 2021 reached or maximally tolerable for this patient<br>-1= Unknown	**(X)**	**X**	**X**
	Additional medication				
	Diuretic	0= No<br>1= Yes (target dose in ESC 2021 = 15mg)<br>-1= Unknown	**(X)**	**X**	**X**
	Vericiguat	0= No<br>1= Yes<br>-1= Unknown	**(X)**	**X**	**X**
	Digoxin	0= No<br>1= Yes<br>-1= Unknown	**(X)**	**X**	**X**
	Amiodarone	0= No<br>1= Yes<br>-1= Unknown	**(X)**	**X**	**X**
	Sotalol	0= No<br>1= Yes<br>-1= Unknown	**(X)**	**X**	**X**
	Hydralazine / Isosorbide dinitrate	0= No<br>1= Yes<br>-1= Unknown	**(X)**	**X**	**X**
**Outcome variables**	***Cardiac intervention and implantable***				
	Pacemaker – VVI	0= No<br>1= Yes<br>-1= Unknown	**(X)**	**X**	**X**
	Pacemaker – VVI date$	Date (DD-MM-YYYY)	**(X)**	**X**	**X**
	Pacemaker – DDD	0= No<br>1= Yes<br>-1= Unknown	**(X)**	**X**	**X**
	Pacemaker – DDD date$	Date (DD-MM-YYYY)	**(X)**	**X**	**X**
	CRT-P	0= No<br>1= Yes<br>-1= Unknown	**(X)**	**X**	**X**
	CRT-P date$	Date (DD-MM-YYYY)	**(X)**	**X**	**X**
	CRT-D	0= No<br>1= No, because of medical condition<br>2= No, because of patients' wish<br>10= Yes<br>-1= Unknown	**(X)**	**X**	**X**
	CRT-D date$	Date (DD-MM-YYYY)	**(X)**	**X**	**X**
	ICD – VVI	0= No<br>1= No, because of medical condition<br>2= No, because of patients' wish<br>10= Yes<br>-1= Unknown	**(X)**	**X**	**X**
	ICD – VVI date$	Date (DD-MM-YYYY)	**(X)**	**X**	**X**
	ICD – DDD	0= No<br>1= No, because of medical condition<br>2= No, because of patients' wish	**(X)**	**X**	**X**

		10= Yes<br>-1= Unknown			
	ICD – DDD date$	Date (DD-MM-YYYY)	(X)	X	X
	PCI	0= No<br>1= Yes<br>-1= Unknown	(X)	X	X
	PCI date$	Date (DD-MM-YYYY)	(X)	X	X
	CABG	0= No<br>1= Yes<br>-1= Unknown	(X)	X	X
	CABG date$	Date (DD-MM-YYYY)	(X)	X	X
	LVAD	0= No<br>1= Yes<br>-1= Unknown	(X)	X	X
	LVAD date$	Date (DD-MM-YYYY)	(X)	X	X
	Heart transplantation	0= No<br>1= Yes<br>-1= Unknown	(X)	X	X
	Heart transplantation date$	Date (DD-MM-YYYY)	(X)	X	X
	Heart revalidation	0= No<br>1= Yes<br>-1= Unknown	(X)	X	X
	Telemonitoring	0= No telemonitoring<br>10= Telemonitoring by telephone<br>20= Telemonitoring, non-invasive based on traditional parameters (e.g., blood pressure, ECG)<br>30= Telemonitoring, using ICD based on HF parameters<br>40= Telemonitoring, invasive by sensors in the blood stream or heart<br>-1= Unknown	(X)	X	X
	Patient status and quality of life				
	Mortality	0= Alive<br>1= Deceased<br>-1= Unknown		X	X
	Date mortality$	Date (DD-MM-YYYY)		X	X
	Number of hospitalizations	Number		X	X
	Date hospitalization$	Date (DD-MM-YYYY)		X	X
	Number of hospitalization days	Number		X	X
	Location follow-up	0= No follow-up, because of mortality<br>10= Patient stays in secondary care, outpatient HF clinic<br>20= Patient stays in tertiary care, outpatient HF clinic<br>30= Patient transferred to primary care<br>40= Patient transferred to the cardiology outpatient clinic<br>90= Other<br>-1= Unknown		X	X
	Type quality of life questionnaire	0= Not measured<br>10= SF12-2<br>20= SF36-2<br>30= SF36-1<br>-1= Unknown	X	X	X
	Quality of life	Coding depends on questionnaire	X	X	X

*Variables are collected in the Heart failure registry, but the data is not distributed and available for research.

$All variables which consist of a date will be transformed to number of days since diagnosed with HF. The actual date is not available in research.

HF: Heart Failure; HFrEF: Heart Failure with reduced Ejection Fraction; HFmrEF: Heart Failure with mildly reduced Ejection Fraction; HFpEF: Heart failure with preserved Ejection Fraction; NYHA: New York Heart Association; eci: e causa ignota (unknown cause); NT-proBNP: N-Terminal pro B-type Natriuretic Peptide; ACE: Angiotensin Converting Enzyme; td: target dose; ARB: Angiotensin Receptor Blocker; ARNI: Angiotensin Receptor-Neprilysin Inhibitor; MRA: Mineralocorticoid Receptor Antagonists; SGLT2: Sodium-glucose co-transporter-2; VVI: Ventricular pacing; DDD: dual-chamber antibradycardia pacing; CRT-P: Cardiac Resynchronization Therapy with a Pacemaker; CRT-D: Cardiac Resynchronization Therapy with a pacemaker and an ICD; ICD: Implantable Cardioverter-Defibrillator; PCI: Percutaneous Coronary Intervention; CABG: Coronary Artery Bypass Graft; LVAD: Left Ventricular Assist Device; ECG: Electro Cardio Gram; SF12/SF36: Short Form Health Survey with 12 questions or 36 questions.

van Eijk J, *et al. BMJ Open* 2024; 14:e078021. doi: 10.1136/bmjopen-2023-078021
