# Supplementary material for: RELEASE-HF study: a protocol for an observational, registry-based study on the effectiveness of telemedicine in heart failure in the Netherlands
Source: BMJ Open. 2024 Jan 4;14(1):e078021. doi: 10.1136/bmjopen-2023-078021 (PMC10773380; doi:10.1136/bmjopen-2023-078021)
Supplement: Supplementary data [file bmjopen-2023-078021supp002.pdf]

Supplementary material 2 Interview guide telemedicine

Introduction
What do we mean by telemedicine?
With telemedicine, we mean the provision of care at a distance. The healthcare provider and the patient are not physically present with each other at that moment. Telemedicine can consist of telemonitoring or tele-education (according to the Collaboration Agreement and Quality Criteria for Telemedicine).

- *Telemonitoring* involves monitoring heart failure symptoms and relevant parameters used to support patients and healthcare providers in the treatment of heart failure.
- *Tele-education* is a service that allows the patient/user to receive remote education. Receiving this education can be accessed through a variety of applications and devices.

Background Information: Use of Telemedicine

1) Does your hospital offer telemedicine to outpatient heart failure patients?
    - Yes, we offer telemedicine.
        - i. Why do you offer telemedicine?
            - ➢ *Possibilities: monitoring, education, self-care/self-management, consultation, cost reduction, reduction in hospital admissions, improvement in quality of life, reducing workload.*
    - No, we do not offer telemedicine.
        - i. Why don't you offer telemedicine? (How strong is the influence of [x] in this?)
            - ➢ *Possibilities: no patient/clinician demand, costs, evidence, user-unfriendly technology, no connection to EHR, HF care pathway is different, time, shortage of healthcare professionals, significant investment compared to the amount of patients, legal/privacy concerns, others.*
        - ii. Have you considered using telemedicine for HF patients?
            - ➢ What prompted you to consider it but not proceed?
        - iii. Which professionals were involved in this decision?
    - No, but we plan to start telemedicine.
        - i. Why are you planning to start telemedicine?
            - ➢ *Possibilities: patient/clinician desire, guidelines, COVID-19, funding, others.*
        - ii. To what extent did COVID-19 influence this decision?
        - iii. Which professionals are involved in this decision?
    - No, we stopped telemedicine.
        - i. Why did you stop telemedicine?
            - ➢ *Possibilities: no patient demand, didn't meet expectations, more time, moderate positive effects, cost, lack of staff, others.*
        - ii. Who were involved in this decision? (Professionals)

Users of Telemedicine
From this point on, the conversation continues with the focus on the performers of telemedicine from the perspective of healthcare professional.

van Eijk J, *et al. BMJ Open* 2024; 14:e078021. doi: 10.1136/bmjopen-2023-078021

2) Which system do you use for telemedicine?
   - Luscii
   - Sananet/SanaCoach
   - Motiva (Philips)
   - 24Care
   - Hartwacht
   - CardioMEMS
   - Empower
   - Other, namely: ...

3) Why do you use telemedicine for heart failure patients?
   - Why is telemedicine used for [goal X]?
      i. *Functionalities: monitoring, education, self-care/self-management, consultation.*
      ii. *Endpoints: cost reduction, reduction in hospital admissions, improvement in quality of life, reducing workload for healthcare professionals.*
      iii. *Other, namely: ...*

Offering Telemedicine

4) Which patients visit the heart failure outpatient clinic?
      i. *Severity, age, duration of illness.*

5) Which heart failure patients at your outpatient clinic are eligible for telemedicine?
      i. What factors are considered in the decision to use telemedicine?
      ii. *Telemedicine guideline: Chronic HF, recently diagnosed, readmission due to exacerbation, anxious/uncertain/depressive patients, assistance in early detection of deterioration.*
      iii. *Characteristics: caregiver network, patient/donor digital skills, language proficiency, patient mobility, internet connection, distance to hospital, other.*
   - Does the severity of heart failure play a role in initiating telemedicine?
      - Yes
      - No
   - At which NYHA class is telemedicine used?
      - NYHA 1
      - NYHA 2
      - NYHA 3
      - NYHA 4
   - Why this NYHA class?

6) Which heart failure patients at your outpatient clinic are not eligible for telemedicine?
   - Why are these patients not eligible?
      i. *Telemedicine guideline: Chronic HF, recently diagnosed, readmission due to exacerbation, anxious/uncertain/depressive patients, assistance in early detection of deterioration.*
      ii. *Characteristics: caregiver network, patient/donor digital skills, language proficiency, patient mobility, internet connection, distance to hospital, other.*

7) When is telemedicine for the first time considered for heart failure patients? (multiple answers possible)
   - For every newly diagnosed heart failure patient
   - After a hospital admission

- For patients attending the heart failure outpatient clinic (regardless of previous hospital admission)
- After an exacerbation (patient is already diagnosed with heart failure)
  i. When is telemedicine offered after an exacerbation?
    - After 1 exacerbation
    - After ... exacerbations
    - If the patient has more than ... exacerbations
  i. Does the time period in which the patient has an exacerbation play a role in offering telemedicine?
    - Yes, when ... exacerbations in ... weeks
    - No
    - In the titration phase
  i. Is telemedicine offered during the titration phase?
    - Yes, every time there is a change in medication
    - Yes, only when the patient is newly diagnosed with the disease
    - Sometimes, depending on the situation, ease of titration, patient's distance, pandemic, etc.
    - No
  i. For which medication?
- In the stable phase of HF
  i. Is telemedicine offered in every stable phase of heart failure?
    - Yes
    - No
- Others, namely: ...

8) Which healthcare professionals are involved in determining whether or not to use telemedicine for the patient? (multiple answers possible)
   - Cardiologist
   - Heart failure nurse
   - Nurse Specialist / Physician Assistant
   - General Practitioner
   - Home care nurse
   - General practice nurse
   - Other, namely: ...

Stopping/On-Off principle of telemedicine

9) Is telemedicine offered multiple times in a heart failure care trajectory?
   - Yes
     i. How is this offered?
     ii. For which patients?
     iii. What determines the on/off/on/off principle?
     iv. Is the equipment removed?
   - No

10) When is telemedicine stopped?
    i. *Patient request, death, healthcare professional's assessment, disease severity, treatment phase, established time period, other*
    - Does NYHA class play a role in this?

Modules of the Telemedicine System

11) On which device can the patient receive telemedicine? *(multiple answers possible)*
    - o Application on a mobile phone (smartphone)
    - o Computer
    - o Television
    - o Watch (smartwatch)
    - o iPad
    - o Other, namely: ...

Telemonitoring

12) Does your telemedicine system include telemonitoring (e.g., vital signs, nutrition, medication, heart failure symptoms)?
    - o Yes
        - i. What is the main reason for using telemonitoring?
            - ▪ *Early detection of exacerbation, titration, longitudinal follow-up, self-management, other*
    - o No

13) What type of telemonitoring do you use?
    - o Non-invasive
    - o Invasive
    - o Non-invasive and invasive

14) Which measurements are monitored via telemonitoring?
    - o Weight
    - o Blood pressure
    - o Heart rate
    - o Heart rhythm (irregular, regular)
    - o Temperature
    - o Saturation
    - o Intake (e.g., in the context of sodium restriction)
    - o Fluid intake
    - o Medication
    - o Heart failure-related symptoms
        - i. What symptoms do you monitor?
            - ▪ *Fatigue, decreased appetite, shortness of breath, swollen legs and ankles, cold hands and feet, a full feeling in the upper abdomen, palpitations, nocturia, constipation, tickling cough, restless sleep, memory and concentration problems, dizziness*
            - ▪ *Other, namely: ….*
    - o Other, namely: ....

15) What equipment has the patient received for telemonitoring? *(multiple answers possible)*
    - o No equipment
    - o Patient uses their own equipment
        - i. What equipment?
    - o Weight scale
    - o Bluetooth-enabled weight scale
    - o Blood pressure monitor
    - o Bluetooth-enabled blood pressure monitor
    - o Pulse oximeter
    - o Bluetooth-enabled pulse oximeter
    - o ECG device

van Eijk J, *et al. BMJ Open* 2024; 14:e078021. doi: 10.1136/bmjopen-2023-078021

- CardioMEMS
- Other, namely: ....

16) Can a patient transmit monitored (vital) signs via the telemonitoring system to the healthcare provider?
    - Yes
        i. When can the patient transmit these monitored (vital) signs? (time of day)
            - *Possible options: 24/7, office hours, weekend, evening, night, set times, other, namely*
        ii. How does the patient transmit the monitored (vital) signs to the healthcare professional? (communication)
            - *Possible options: letter, phone call, video call, chat, SMS, email, via applications, other*
    - No

17) Does the healthcare professional receive a notification when monitored (vital) signs are entered into the telemonitoring system?
    - Yes
        i. When does the healthcare professional receive a notification?
            - *Possible options: always, when threshold values are exceeded, other*
        i. Where does the healthcare professional receive the notification?
            - *Possible options: Electronic Patient Record (EPD), standalone applications, email inbox, other*
        ii. Does the healthcare professional always receive the notification in the same manner, or does severity of exceeding the threshold play a role?
            - Yes
            - No
                i. What is the difference?
        iii. Which healthcare professional receives these messages?
            - *Possible options: cardiologist, heart failure nurse, nurse practitioner, physician assistant, general practitioner (GP), practice nurse, home care worker, other*
        iv. Are there erroneous notifications?
            - *How do you handle them?*
    - No
        v. How often does the healthcare professional evaluate the monitored vital signs, questionnaires, outcomes filled in by the patient in the telemonitoring system?
            a. *Per week: ....*
            b. *Per day: ....*
            c. *Per patient: ....*

18) Can a healthcare professional respond to the received monitored (vital) signs?
    - Yes
        i. Which healthcare professional responds to the received results?
            - *Possible options: cardiologist, heart failure nurse, nurse practitioner, physician assistant, general practitioner (GP), practice nurse, home care worker, other*
        ii. When can the healthcare professional respond to these monitored (vital) signs? (time of the day)
            - *Possible options: 24/7, office hours, weekend, evening, night, set times, other*
        iii. How does the healthcare professional respond to the monitored (vital) signs?
            - *Possible options: letter, phone call, video call, chat, SMS, email, via applications, other*
        iv. Does the patient receive a notification if the healthcare professional has sent a message to the patient?
    - No

19) Can thresholds be set for the monitored (vital) signs in the telemonitoring system?
   - No
   - Yes, general thresholds (not patient-specific)
      i. What are the thresholds based on? / How are the thresholds formulated?
   - Yes, patient-specific thresholds
      i. Can the thresholds be adjusted per patient, per situation, per moment?
         - Yes
         - No
      i. What are the thresholds based on? / How are the thresholds formulated?

20) Is there always contact between the patient and the healthcare professional for the monitored (vital) signs transmitted via the telemonitoring system?
   - Yes
      i. Is the contact direct or indirect (store-and-forward) between the patient and the healthcare professional?
         - *Direct: The patient and healthcare professional are using the system simultaneously, allowing for direct contact via one of the techniques provided by the telemonitoring system.*
         - *Indirect: The patient and healthcare professional have asynchronous contact with each other. The patient/healthcare professional sends their information to the recipient. The recipient reads the message later and responds to the sender.*
         - *Combination of direct and indirect contact*
   - No
      i. When is there contact between the patient and healthcare professional?

21) What actions does the healthcare professional take with the received monitored (vital) signs?
      i. How do you initiate these actions?
         - *Possible options: letter, phone call, video call, chat, SMS, email, via applications, other*
      ii. Does the telemonitoring system provide automatically generated advice to the patient when they enter data in a module in the telemonitoring system?
         - *Yes*
         - *No*

22) What is your experience with the telemonitoring provided by the telemonitoring system?

Education and self-care

23) Does your telemedicine system include the modules education and self-care?
   - Yes
      i. Does the education module need to be actively activated for each patient by the healthcare professional? (Note: Can the telemonitoring system also exist without the education module?)
         - Yes
         - No
      ii. Which topics are included in the education?
         - General medical information about heart failure
         - Information about treatment
         - Prevention of symptoms/exacerbations
         - Medication use
         - Fluid and dietary management
         - Physical activity
         - Other, namely: ...

iii. Do you actively refer the patient to the education module in the telemedicine system? (Note: Do you discuss this topic in your consultation?)
   - Yes
   - No

i. Is the education module the same for every patient?

ii. Which self-care components are included in the telemedicine system?
   - Lifestyle interventions,
     - Which behaviors/lifestyle interventions are included?
       - *Possible answers: exercise, nutrition, stress management*
   - Psychological well-being: information, knowledge, tools
   - Patient's medication overview
   - Medication explanation (medication glossary)
   - Medication intake reminder as per prescription
   - Referral to external sources and links related to heart failure
   - Knowledge quiz/ability to test knowledge about heart failure
   - Monitoring
   - Self-detection and the ability to anticipate/adjust medication (e.g., detecting weight gain through monitoring and then taking and recording medication [diuretics])
   - Other, namely: ...

- No

24) To what extent does the patient make decisions based on the telemedicine system?
   - Are efforts made that the patient take independent action / anticipate in response to measured (vital) signs?

25) To what extent does the telemedicine system promote the patient's self-care? / What is the effect of this module on the patient's self-care (skills)?

26) What are your experiences with the built-in self-care and education modules in the telemedicine system?

Heart Failure Community

27) Can the patient use the telemedicine system to get in touch with peers (for example, a heart failure community)?
   - Yes, always
   - Yes, but this module needs to be activated
     i. How is the heart failure community organized?
       - *Local (hospital)*
       - *Regional*
       - *National*
   - No, this is not possible

Analog Telemonitoring (via telephone)

28) Do you have contact with heart failure patients by phone (hospital to patient)?
    - Yes
        i. Who initiates contact with these patients by phone?
            - *Possible options: cardiologist, heart failure nurse, nurse practitioner (NP), physician assistant (PA), general practitioner (GP), general practice nurse (POH), home care nurse, other*
        ii. When do you have contact with the patient by phone?
        iii. What is the purpose of contact by phone?
        iv. How often do you have contact with the patient by phone?
        v. Which patients receive telephone guidance?
        vi. Why these patients?
    - No

29) Can the patient initiate contact by phone with the heart failure outpatient clinic (patient to hospital)?
    - Yes
        i. When can/may the patient initiate this type of contact?
        ii. Who does the patient have telephone contact with?
            - *Possible options: cardiologist, heart failure nurse, nurse practitioner (NP), physician assistant (PA), general practitioner (GP), general practice nurse (POH), home care nurse, other*
    - No

30) Can a heart failure patient or healthcare professional seek contact in any other way?
    - Through which channel does this contact occur?
        - *Possible answer: patient portal*
    - When does this contact occur via this channel?

31) What is your experience with telemonitoring by phone?

External Influences on the Use of Telemonitoring
Evaluation

32) Is the telemedicine system evaluated (experiences, application in HF care, impact on objectives) among healthcare professionals?
    - Yes
    - No

33) What is the influence of telemedicine on the patient population that visits the (regular) heart failure outpatient clinic?
    - What are the characteristics of these patients?

34) Is the telemedicine system evaluated (experiences, application in their disease, usage) between the healthcare professional and the patient?
    - Yes
    - No
        i. Are expectations discussed with the patient regarding the use of telemedicine?
        ii. What feedback do you receive?
        iii. What is the experience of telemedicine from the patients' perspective?

Experience

35) Do you believe that telemedicine in your hospital has led to efficiency?
    - Yes
        i. On which outcomes?
        ii. Has this been objectively assessed?
    - No
        i. What makes you say that?

36) Would you recommend the telemedicine system you use?
    - Why?

37) What would you like to change/improve about your telemedicine system?

38) If you could choose a telemedicine system again, what would it look like?

COVID-19

39) Does COVID-19 have an impact on the use of telemedicine in your heart failure outpatient clinic? (multiple answers possible)
    - Yes, telemedicine is used for more patients as an intervention.
    - Yes, telemedicine is used less frequently for patients as an intervention.
    - Yes, telemedicine is used differently by patients who were already using this intervention before COVID-19.
        i. Which devices of the telemedicine system are used differently since COVID-19?
            - *Possible answers: phone calls, video calls, chat, email, SMS, chatbot, other.*
        i. Which modules of the telemedicine system are used differently since COVID-19?
            - *Possible answers: telemonitoring, education, self-care/self-management, heart failure community, other.*
        i. What do you think of this development?
    - No, there is no difference in the use of telemedicine before, during, and in the current COVID-19 period.

Time Investment in telemedicine vs. No telemedicine in Heart Failure Care (HTA)

40) How much time do you, on average, spend on an appointment at a heart failure outpatient clinic without telemedicine?
    ... minutes / hours

41) How much time do you, on average, spend on an appointment at a heart failure outpatient clinic with telemedicine?
    ... minutes / hours

42) How much time does it take to explain telemedicine to a heart failure patient (for the initial setup)?
    ... minutes / hours

43) How much time do you, on average, spend per week on providing telemedicine? (this includes monitoring digital statuses transmitted through the telemonitoring system, contacting the patient as needed, evaluating measurements, etc.)
    ... minutes / hours
