# Supplementary material for: RELEASE-HF study: a protocol for an observational, registry-based study on the effectiveness of telemedicine in heart failure in the Netherlands
Source: BMJ Open. 2024 Jan 4;14(1):e078021. doi: 10.1136/bmjopen-2023-078021 (PMC10773380; doi:10.1136/bmjopen-2023-078021)
Supplement: Author's manuscript [file bmjopen-2023-078021.draft_revisions.pdf]

BMJ Open is committed to open peer review. As part of this commitment we make the peer review history of every article we publish publicly available.

When an article is published we post the peer reviewers' comments and the authors' responses online. We also post the versions of the paper that were used during peer review. These are the versions that the peer review comments apply to.

The versions of the paper that follow are the versions that were submitted during the peer review process. They are not the versions of record or the final published versions. They should not be cited or distributed as the published version of this manuscript.

BMJ Open is an open access journal and the full, final, typeset and author-corrected version of record of the manuscript is available on our site with no access controls, subscription charges or pay-per-view fees (http://bmjopen.bmj.com).

If you have any questions on BMJ Open's open peer review process please email info.bmjopen@bmj.com

BMJ Open

Study protocol for a multi-center registry-based nationwide study on the effectiveness of telemedicine in heart failure patients: the RELEASE-HF study

Journal:	*BMJ Open*
Manuscript ID	bmjopen-2023-078021
Article Type:	Protocol
Date Submitted by the Author:	26-Jul-2023
Complete List of Authors:	van Eijk, Jorna; University Medical Centre Utrecht, Department of General Practice & Nursing Science, Julius Center for Health Sciences and Primary Care<br>Luijken, Kim; University Medical Centre Utrecht, Department of Epidemiology & Health Economics, Julius Center for Health Sciences and Primary Care<br>Jaarsma, Tiny; University Medical Centre Utrecht, Department of General Practice & Nursing Science, Julius Center for Health Sciences and Primary Care<br>Reitsma, Johannes; University Medical Centre Utrecht, Department of Epidemiology & Health Economics, Julius Center for Health Sciences and Primary Care<br>Schuit, Ewoud; University Medical Centre Utrecht, Department of Epidemiology & Health Economics, Julius Center for Health Sciences and Primary Care<br>Frederix, Geert; Universitair Medisch Centrum Utrecht, Department of Epidemiology & Health Economics, Julius Center for Health Sciences and Primary Care<br>Derks, Lineke; Netherlands Heart Registration<br>Schaap, Jeroen; Amphia Hospital, Department of Cardiology; WCN, Dutch Network for Cardiovascular Research<br>Rutten, Frans; University Medical Centre Utrecht, Department of General Practice & Nursing Science, Julius Center for Health Sciences and Primary Care<br>Brugts, Jasper; Erasmus Medical Center, Department of Cardiology<br>de Boer, Rudolf; Erasmus Medical Center, Department of Cardiology<br>Asselbergs, Folkert; Amsterdam University Medical Centres, Department of Cardiology; University College London, Health Data Research UK and Institute of Health Informatics<br>Trappenburg, Jaap; University Medical Centre Utrecht, The Healthcare Innovation Center, Julius Center for Health Sciences and Primary Care<br>investigators, RELEASE-HF ; University Medical Centre Utrecht
Keywords:	Heart failure < CARDIOLOGY, Telemedicine < BIOTECHNOLOGY & BIOINFORMATICS, REGISTRIES, HEALTH ECONOMICS, Protocols & guidelines < HEALTH SERVICES ADMINISTRATION & MANAGEMENT, Clinical Decision-Making

SCHOLARONE™
Manuscripts

Study protocol for a multi-center registry-based nationwide study on the effectiveness of telemedicine in heart failure patients: the RELEASE-HF study.

Authors:

Jorna van Eijk, MSc RN[1]*
Kim Luijken, PhD[2]
Tiny Jaarsma, PhD RN[1]
Johannes B Reitsma, PhD[2]
Ewoud Schuit, PhD[2]
Geert WJ Frederix, PhD[2]
Lineke Derks, MSc[3]
Jeroen Schaap, MD PhD[4, 5]
Frans H Rutten, MD PHD[1]
Jasper J Brugts, MD PhD MSc[6]
Rudolf A de Boer, MD PhD[6]
Folkert W Asselbergs, MD PhD[7, 8]†
Jaap CA Trappenburg, PhD[9] †
RELEASE-HF investigators

RELEASE-HF investigators (group authorship/collaborator):

C Jan Willem Borleffs, MD PhD[10]
Dirk H van Dalen, MSc RN[11]
Ayten Erol-Yilmaz, MD PhD[12]
M Louis Handoko, MD PhD MSc[7, 13]
Gerardus PJ van Hout, MD[14, 15]
Jaco Houtgraaf, MD PhD[16]
Wouter W Jansen Klomp, MD MSc[17]
Stefan Koudstaal, MD PHD MSc FESC FHFA[18]
Gerard CM Linssen, MD PhD FESC FHFA[19]
Manon G van der Meer, MD PhD[15]
Marco C Post, MD, PhD[14, 15]
Sandra Sanders-van Wijk, MD PhD[20]
Eric Wierda, MD PhD[21]
Stijn CW Wouters, MD PhD[22]

For peer review only - http://bmjopen.bmj.com/site/about/guidelines.xhtml

1 Department of General Practice & Nursing Science, Julius Center for Health Sciences and Primary Care, University Medical Center Utrecht, Utrecht University, Utrecht, the Netherlands
2 Department of Epidemiology & Health Economics, Julius Center for Health Sciences and Primary Care, University Medical Center Utrecht, Utrecht University, Utrecht, The Netherlands
3 Netherlands Heart Registration, Utrecht, The Netherlands
4 Department of Cardiology, Amphia Hospital, Breda, The Netherlands
5 Dutch Network for Cardiovascular Research (WCN), Utrecht, The Netherlands
6 Department of Cardiology, Erasmus Medical Center, Rotterdam, the Netherlands
7 Department of Cardiology, Amsterdam University Medical Centers, VU University, Amsterdam, The Netherlands
8 Health Data Research UK and Institute of Health Informatics, University College London, London, United Kingdom
9 The Healthcare Innovation Center, Julius Center for Health Sciences and Primary Care, University Medical Center Utrecht, Utrecht University, Utrecht, the Netherlands
10 Department of Cardiology, Haga Teaching Hospital, Den Haag, the Netherlands
11 Department of Cardiology, Jeroen Bosch Hospital, 's-Hertogenbosch, the Netherlands
12 Department of Cardiology, Bernhoven Hospital, Uden, the Netherlands
13 Amsterdam Cardiovascular Sciences, Heart Failure and Arrhythmias, Amsterdam, The Netherlands
14 Department of Cardiology, St. Antonius Hospital, Nieuwegein/Utrecht, the Netherlands
15 Department of Cardiology, University Medical Center Utrecht, Utrecht, the Netherlands
16 Department of Cardiology, Diakonessenhuis, Utrecht, the Netherlands
17 Department of Cardiology, Deventer hospital, Deventer, the Netherlands
18 Department of Cardiology, Groene Hart Ziekenhuis Gouda, Gouda, the Netherlands
19 Department of Cardiology, Hospital Group Twente, Almelo & Hengelo, the Netherlands
20 Department of Cardiology, Zuyderland Medical Center, Heerlen & Sittard-Geleen, the Netherlands
21 Department of Cardiology, Dijklander hospital, Hoorn, The Netherlands
22 Department of Cardiology, Maasziekenhuis Pantein, Beugen, the Netherlands

***Corresponding author.** Department of General Practice & Nursing Science, Julius Center for Health Sciences and Primary Care, University Medical Center Utrecht, Utrecht University, Heidelberglaan 100, 3584 CX Utrecht, the Netherlands. Email: j.vaneijk-4@umcutrecht.nl

† **Contributed equally**

Word count:
4291

For peer review only - http://bmjopen.bmj.com/site/about/guidelines.xhtml

ABSTRACT

Introduction

Telemedicine gradually gains acceptance in heart failure (HF) management and meta-analyses show positive effects of telemedicine on hospitalization, mortality, and costs. These effects, however, are heterogeneous across studies due to variation in the included HF population, telemedicine components, and the quality of the comparator usual care. In this nationwide study we aim to identify (i) in which subgroup(s) of HF patients telemedicine is (cost-)effective, and (ii) which components of telemedicine are most (cost-)effective.

Methods and analysis

The 'REsponsible roLl-out of E-heAlth through Systematic Evaluation – Heart Failure' study (RELEASE-HF) is a multi-center, registry-based cohort study planning to enroll 6480 HF patients using data from the HF registry facilitated by the Netherlands Heart Registration. Patient characteristics, treatment information, and clinical outcomes are collected at baseline, 6 months, and 12 months from time since diagnosis. Components of telemedicine will be described based on close-ended interviews with clinicians on hospital level and on patient level added with extracted data from Electronic Health Records and telemedicine generated data. Costs of telemedicine are calculated using registration data and interviews with clinicians and finance department staff. To overcome missing data and prevent administrational burden, additional national databases will be linked to the HF registry if feasible. Heterogeneity of effects of offering telemedicine compared to not offering on days alive without unplanned hospitalizations in 1 year will be assessed across predefined patient characteristics using exploratory stratified analyses. Effects of telemedicine components will be assessed by fitting separate models for three component contrasts.

Ethics and dissemination

The study has been approved by the medical ethics committee 2021 at University Medical Center Utrecht (The Netherlands). Results will be published in peer-reviewed journals and presented at (inter)national conferences. Effective telemedicine scenarios will be proposed amongst hospitals, throughout the country and abroad, if applicable and feasible.

Trial registration number

NCT05654961

Key words

Heart failure
Telemedicine
Clinical effectiveness
Cost effectiveness
Registry
Study protocol

For peer review only

STRENGHTS AND LIMITATIONS OF THIS STUDY

- This study will use a nationwide registry-based cohort linked to national registries and databases to capture real world data on telemedicine use, heart failure patient characteristics, treatments, and clinically relevant outcomes with a 1-year follow-up.
- Data of the Heart failure registry in the Netherlands and a separate data collection on telemedicine features by additional questionnaires allow consideration of heterogeneous treatment effects across the heart failure population and telemedicine components.
- Most data on telemedicine characteristics will be collected on a hospital level rather than on the individual patient level.
- Cost-effectivity analyses are specific for the Dutch healthcare system, which may hamper generalizability of findings to other settings or countries.
- Since the Heart failure registry is not incorporated in care pathways in Electronic Health Record systems, selection bias can occur when healthcare providers do not include all patients in the outpatient clinic in the Heart failure registry.

For peer review only

INTRODUCTION

Heart failure (HF) is a major public health problem, affecting over 64.3 million people worldwide.[1] It is a clinical syndrome associated with an unpredictable but in general progressive disease trajectory, characterized by frequent exacerbations, often leading to increase in symptoms, decreased health-related quality of life (hrQoL), accelerated decline of cardiac function, (re-)hospitalisation, and increased risk of death.[2, 3] It is associated with a large economic and social burden on society.[2, 4-6]

Optimal medical management, but also self-care (i.e., healthy diet, medication adherence, exercise), and adequate monitoring of vital signs and symptoms may all help to reduce HF-related morbidity.[2, 7, 8] Recently, interest has gone out to telemedicine as a tool to support patients in optimizing HF management, self-care support and symptom monitoring to improve care and prevent (re-)hospitalization.[7-9] Additionally, due to the aging population, the number of patients with HF will increase significantly in the future. Telemedicine could be a solution to keep HF care manageable when it is integrated in care pathways.[10, 11] “Telemedicine” is a very heterogeneous intervention, and includes a wide range of digital technologies (smartphones, mobile wireless devices/sensors, video connections, implantable devices, etc.) that exchange digital health information between patient and clinician to support and optimize the care process remotely.[12, 13]

A large number of meta-analyses has evaluated the (cost-)effectiveness of telemedicine for HF patients. Overall, these meta-analyses point towards a positive effect of telemedicine on hospital admission, length of hospital stay, mortality, and costs[7, 14-26], but the effects are heterogeneous across subgroups.[27-31] In addition, the effects differ depending on variation in telemedicine components, such as type of monitored functions, number of alerts and risk of alert fatigue, contact with health care professional, type of device, and different quality of usual care.[14, 21, 22, 26, 32] Finally, in depth evaluation of the cost-effectiveness of telemedicine is missing because of variety in calculating costs, i.e. purchase costs, staff costs, and components of telemedicine.[9, 19, 23]

As a result, HF guidelines lack specific recommendation on *how, when and in whom* (stable or unstable HF patients*)* telemedicine should be provided. Despite the lack of solid evidence and advice, payers (e.g., procurement officers, insurance companies) and patient organizations are advocating to accelerate implementation of telemedicine for HF patients in outpatient clinics.[33] Consequently, telemedicine is implemented in different formats with varying objectives, intervention components and implementation strategies. To reveal the real potential of telemedicine for HF patients and its implementation in everyday practice, clarity on *how, when and in whom,* and which telemedicine components are (cost-)effective, is essential.

Here, we present the protocol for the ‘REsponsible roLl-out of E-heAlth through Systematic Evaluation – Heart Failure’ study (RELEASE-HF). In this study, we focus on both patient-related uncertainties in telemedicine use and telemedicine intervention-related uncertainties, from a clinical as well as an economical perspective. Our specific study objectives are to examine: (1) which patient characteristics are related to an increase in number of days spent alive without unplanned hospitalizations within one year when telemedicine is part of HF care compared to regular HF care; (2) which components of telemedicine as part of HF care lead to an increased number of days alive without unplanned hospitalizations within one year; (3) which HF patient characteristics are related to cost-effectiveness when telemedicine is part of HF care compared to regular HF care; and (4) which components of telemedicine as part of HF care are cost-effective.

For peer review only - http://bmjopen.bmj.com/site/about/guidelines.xhtml

METHODS AND ANALYSIS

Study design

RELEASE-HF is a nationwide, registry-based cohort study across multiple hospitals in the Netherlands.[34] The study has an observational character, collecting routine data longitudinally. We record the health status of and cardiac interventions in HF patients, and simultaneously observe the natural (de)implementation of telemedicine. RELEASE-HF is linked to the Heart4Data consortium that started in 2022 to create a national and sustainable infrastructure for cardiovascular registry-based research in the Netherlands.[35] This infrastructure will develop a framework for the governance, ethical, legal, financial, information technology and methodological factors necessary for registry-based research.

Progress and time plan

RELEASE-HF started in June 2021 and will last four years (Figure 1). This study includes a collaboration with at least 29 hospitals that signed a letter of commitment. Additional hospitals may enter the study later if they can complete a 12-month follow-up per included patient through the HF registry.

Data sources

RELEASE-HF will combine various sources of data: the Netherlands Heart Registration (NHR) HF registry, other national databases, interviews, electronic health records (EHR), and telemedicine generated data (Figure 2). A detailed description of each data source is provided below.

Heart failure registry

The HF registry is an ongoing quality registry in the Netherlands, facilitated by the NHR; a non-profit organization that aims to contribute to quality improvement and safety in cardiac care.[36, 37] The HF registry serves as an ongoing learning healthcare system through benchmarking and quality control. Since 2019, it has been introduced as a voluntary, nationwide HF registration. Patient data is collected nonconsecutively and for the registry no informed consent is asked. Data of included patients are collected at three timepoints: at baseline, 6 months, and 12 months from time since diagnosis. The HF registry includes variables on three levels and are defined in a data dictionary: patient characteristics, treatment characteristics and clinically relevant outcomes. The data dictionary is a dynamic document (i.e., included variables and definitions may change over time) because it is based on current HF guidelines and compiled by a committee consisting of delegated cardiologists from Dutch hospitals. A detailed up to date data dictionary with an overview of the variables collected within the HF registry is always published at the NHR website (Table S1 provides an overview of the data dictionary of the HF registry, version September 2022. The content is subject to change).[38]

For peer review only - http://bmjopen.bmj.com/site/about/guidelines.xhtml

National registries and databases

If legally and technically feasible, we plan to link the HF registry data, specific for RELEASE-HF, to external data sources to (1) complement missing variables, (2) enrich and validate the HF registry, and (3) reduce registration burden for health care professionals/data managers. The external data sources considered are Statistics Netherlands (CBS), national health insurance database (VEKTIS), national registry for hospital care (Dutch Hospital Data (DHD)) and national database for drug utilization and drug safety (PHARMO). Variables of interests are visits to the outpatient clinic, to the emergency room, and to the general practitioner related to HF, admission days at an intensive care unit, and treatment characteristics specific to HF (i.e., medication).

Interviews on the use of telemedicine

Separate data collection will be performed using semi-structured interviews among clinicians and finance department staff on features and costs of telemedicine on hospital level.

Electronic Health Records and telemedicine generated data

File search in EHR of patients and telemedicine generated data will be used to identify telemedicine components on patient level. Collected data will include the start date of telemedicine use, and the frequency in which measurements are sent to the treating hospital, with a high rate typically referred to as a high-intensity (or acute) protocol and a low rate as a low-intensity (or stable) protocol.

Study population

The HF registry comprises patients admitted to a Dutch hospital or outpatient clinic for HF (irrespective of telemedicine use), where all phenotypes of HF that meet the ESC 2021 guidelines on HF are included.[2] If a patient has been diagnosed with HF in a setting other than the one where the patient currently presents (primary, secondary or tertiary care), the patient will also be included.[38] Current HF registry is not yet incorporated and implemented in care pathways in EHR systems, therefore selected patients fulfilling the NHR criteria are included in the HF registry and not on a consecutive basis or full coverage of all patients at the outpatient HF clinic. This may lead to selection bias. To prevent this selection bias, a researcher of RELEASE-HF is alerting health care professionals regularly to include all patients with HF in the HF registry, regardless of their telemedicine use, treatment and disease severity. Additionally, national working groups are progressing to fully incorporate the HF registry into the EHR.

Outcome measures

Primary outcome

Number of days alive without unplanned hospitalization within one year of follow-up is derived from the number of unplanned hospital days as collected in the HF registry (Table 1).

For peer review only - http://bmjopen.bmj.com/site/about/guidelines.xhtml

Table 1 Operationalization of exposure variable and study outcomes

Variable	Source	Time of collection	Operationalization
**Exposure**			
**Telemedicine**	HF registry, telemedicine generated data	T0, T1[a], T2	Whether a patient receives telemedicine. Five categories: 1. No telemonitoring[b], 2. Telemonitoring by telephone, 3. Telemonitoring, non-invasive based on traditional parameters (e.g., blood pressure, ECG), 4. Telemonitoring, using ICD based on HF parameters, and 5. Telemonitoring, invasive by sensors in the blood stream or heart.
**Primary outcome**			
**Number of days spent alive without unplanned hospitalizations within one year of follow-up**	HF registry	T1[a], T2	Number of days directly related to unplanned cardiac hospital admission due to HF. Number of admission days will be summed up over a period between follow-up moments and subtracted from 365 days.
**Secondary outcomes**			
**Costs**	Derived from HF registry, external data source, EHR, interviews	T0, T1[a], T2	Costs estimated from patient, disease, and treatment characteristics. Information taken into account includes medication use, whether the patient underwent cardiac interventions (e.g., pacemaker implantation, percutaneous coronary intervention), use of telemedicine, hospital admission days, visits to the outpatient clinic, visits to the emergency room, admission days at an intensive care unit, visits at the GP related to HF.
**All-cause mortality**	HF registry, external data source	T1[a], T2	Mortality status, determined after verification at the Personal Records Database (in Dutch: Basisregistratie Personen). Mortality is independent of HF (all-cause).
**Functional status**	HF registry	T0, T1[a], T2	New York Heart Association (NYHA) classification: a functional classification of patients, based on severity of symptoms and physical activity, with specific attention to fatigue, palpitation, and dyspnea. Scores are linked to one of four NYHA classes. Class I: no limitation, class II: slight limitation, class III: marked limitation, class IV: unable to carry on any physical activity without discomfort.[2]
**Health status**	HF registry	T0, T1[a], T2	SF-36 or SF-12 questionnaire (subset of SF-36)[39]: a validated patient-reported survey of patient health. Both questionnaires consist of eight sections with scores: vitality, physical functioning, bodily pain, general health perceptions, physical role functioning, emotional role functioning, social role functioning, and mental health. Each score is transformed into a 0-100 scale on the assumption that each question carries equal weight. The lower the score the more disability.
**Health related Quality of Life**	HF registry	T0, T1[a], T2	SF-36 or SF-12 questionnaire. Quality Adjusted Life Years (QALY) will be calculated based on the SF-6D: a model in which directly a single, preference-based score can be calculated for the SF-36 and SF-12.[40] Scores range from 0.0 (worst health state) to 1.0 (best health state).
**Healthcare utilization**	External data source, EHR	T1, T2	Healthcare utilization based on: 1) number of outpatient visits, plus 2) number of visits at the general practice related to HF.

[a] The HF registry collects data standard at baseline (T0), after 6 months (T1), after 12 months (T2). RELEASE-HF study is conform the timeframes of the HF registry. Therefore, data will be collected at 6 months, although the outcome measurements are after 12 months.

For peer review only - http://bmjopen.bmj.com/site/about/guidelines.xhtml

[b] The HF registry defines telemedicine as *telemonitoring*.
HF: Heart Failure; EHR: Electronic Health Record; ECG: Electrocardiogram; ICD: Implantable Cardioverter Defibrillator; GP: General Practitioner.

Secondary outcomes

Secondary outcomes are all-cause mortality, functional status, health status, hrQoL, healthcare utilization within one year of follow-up, and costs of care (Table 1).

Exposure measurement

Exposure to telemedicine is measured in the HF registry at 6 months and 12 months follow-up as non-use of telemedicine, telephone only, non-invasive telemedicine, implantable-cardioverter-defibrillator-based telemedicine, or invasive telemedicine (Table 1). Telemonitoring output can be different from the close-ended interviews that correspond to all outpatient clinic contacts in general. Components of the telemedicine intervention will be assessed on hospital level at one time during follow-up period. Table 2 provides an overview and operationalization of main components of the telemedicine intervention that have been described in existing literature or at local (e.g., hospital) level.[14, 41-43] The components will be validated and possibly further refined based on the interviews.

Table 2 Overview and operationalization telemedicine variables that are collected in the qualitative study

Component	Definition	Operationalization (examples)
**Supplier**	(External) Supplier of telemedicine for HF patients	Sanacoach, Luscii, Motiva/Philips, linked in personal environment in EHR
**Purpose of telemedicine**	The intention/motives for which the telemedicine intervention is administered: patient level and/or hospital level	Monitoring, prevent exacerbation, reduce workload, reduce costs, patient centered care
**Considering telemedicine**	First time a clinician considered telemedicine in HF management.	After diagnosis, (re)hospitalization, titration phase
**Structured telephone support**	Structured monitoring by telephone without using applications and devices specific to telemedicine and monitoring HF.	Present or not present
**Applications**	Technologies or platforms on which the patient could receive telemedicine	Smartphone, tablet, laptop, television, smartwatch
**Devices**	Accessory a patient could use to perform telemedicine	Blood pressure device (with or without Bluetooth), weight scale (with or without Bluetooth), smartwatch
**Involved health care workers**	Involved health care workers and their role in considering and executing telemedicine	HF nurse, nurse specialist, cardiologist
**Control centrum**	The presence or absence of a(n) (extern) control centrum to check the submitted measurements and questions	Present or not present
**Use of telemedicine**	Moment a patient could use telemedicine and have contact with clinicians	24/7, office hours, during weekend, at night
**Type of contact**	The manner of contact between the clinician and patient	Direct or indirect (store-and-forward) contact with a clinician
**Measurements**	Type of measurements: vital functions and HF related complaints, used to detect deterioration of and/or to monitor HF	Blood pressure, heart rate, weight, temperature, oxygen level, HF complaints as swelling ankles, nycturia,

For peer review only - http://bmjopen.bmj.com/site/about/guidelines.xhtml

		shortness of breath, tiredness, loss of appetite, coughing/wheezing, dizziness
**Notifications**	Messages from the telemedicine intervention. Notifications can be two-sided: from patient to clinician and vice versa	Automatic or non-automatic generated messages. Notifications present or not present.
**Modifiable aspects**	The presence or absence to set up *thresholds* of monitored vital function, and the possibility to tailor these thresholds per patient, severity, type of HF or other aspects of the telemedicine intervention (e.g., set up a tele-education environment).	Available or not available
**Connection with EHR**	Feature of telemedicine if the intervention is integrated with the EHR. This means that the data entered by the patient through the telemedicine intervention is visible to the clinician in the EHR without using other applications.	Connected or not connected with EHR
**Education**	The presence or absence of an educational environment and the manner how this educational environment is shaped	Present or not present
**Educational topics**	The covered topics in the educational environment of telemedicine	Nutrition, behavior, exercise, medication
**Self-care**	The presence or absence of self-care modules and actions taken by the patient based on a digital advice or measurement	Available or not available
**Protocol**	Local (hospital) protocol which consists definitions on patient level about how often a patient should use telemedicine/monitor the vital signs and HF complaints, depending on the HF complaints or phase (i.e., titration, stable monitoring)	Low-intensity protocol defined as <2 times per week a measurement of vital signs. High-intensity protocol defined as ≥ 2 times per week a measurement of vital signs.
HF: Heart Failure; EHR: Electronic Health Record		

For peer review only

Additional variables

Additional information includes: age at HF diagnosis, sex, social economic status (SES), body mass index (BMI), New York Heart Association classification (NYHA), left ventricular ejection fraction (LVEF), etiology of HF, systolic and diastolic blood pressure, heart rhythm and rate. Comorbidities are chronic respiratory disorders, obstructive sleep apnea syndrome (OSAS), stroke, extra cardiac arterial vascular pathology, diabetes mellitus (DM), hypertension, anemia, chronic kidney disease (CKD), malignancy, heart rhythm disorders, depression, and thyroid disease. Overview of these variables can be found in table S1 and are published in detail at the NHR website.[38] Table 3 provides an overview and operationalization of variables derived from this HF registry.

Table 3 Operationalization of variables calculated from included variables in the HF registry

Variable	Operationalization
**Social economic status**	Score for socioeconomic status (including degree of education, wealth and employment history (WOA)) by zip code area according to 2019 data from the Social Cultural Planning Agency. Divided into quartiles or quintiles, where a 1 is a low SES-WOA score.
**Body mass index**	Derived from weight in kilogram and length in centimeters; calculated as weight / (length*length)

For peer review only - http://bmjopen.bmj.com/site/about/guidelines.xhtml

**Chronic kidney disease**	Based on cut-off values of eGFR calculated from serum creatinine level (collected by HF registry). The HF registry calculates eGFR as:<br>Male: (175*((creatinine level/88,4)^- 1,154)*(age Tn^-0,203)<br>Female: (175*((creatinine level/88.4)^- 1,154)*(age Tn^-0,203))*0,742<br><br>CKD = eGFR ≥60 mL/min/1.73 m²<br>No CKD = eGFR <60 mL/min/1.73 m²
**Depression**	Health Related QoL derived from the SF-12 or SF-36 questionnaire.<br><br>Scoring:<br>0-100 = The lower the score the more disability. The higher the score the less disability i.e., a score of zero is equivalent to maximum disability and a score of 100 is equivalent to no disability.
SES: Social Economic Status; WOA: Wealth, educational level and employment history (in Dutch: welvaart, opleidingsniveau, arbeidsverleden); HF: Heart Failure; CKD: Chronic Kidney Disease; QoL: Quality of Life; SF-12 or SF-36: type of Short Form questionnaire	

For peer review only

Data collection and quality control

Data of the HF registry will be remotely monitored for major outliers and missing variables by the NHR, these processes have been described elsewhere.[37, 44] During the RELEASE-HF study, researchers will perform an additional check on completed data and provide assistance to hospitals to reduce missing values, as well as assistance to reduce the registration burden.

Interviews on telemedicine, using an interview guide with close-ended questions, will be performed by an investigator of RELEASE-HF and conducted with clinicians (e.g., cardiologist, HF nurse, nurse specialist, physician assistant). Interviews will be held during the 36-months period of data collection. All hospitals with telemedicine in HF care will be interviewed once, and the data will be checked with the hospitals once during period of data collection to be aware of changes in the components and status of telemedicine. Per hospital, the six-monthly costs of telemedicine are determined via interviews and registration data (if at hand). The units will be linked to the unit cost guideline provided by the Dutch National Health Care Institute (Dutch: Zorginstituut Nederland).[45] These interviews will be performed by a Health Technology Assessment (HTA) scientist, and conducted with clinicians or finance department staff. All participants provide informed consent.

Patient-level telemedicine data are collected by a clinician, data manager or researcher at the hospital where the patient is being treated, because they have access to the identification log of the HF registry.

The Heart4Data consortium will establish possibilities for data linkage between the HF registry and other real-world data sources, in collaboration with public partners (Figure 2).[35] At the moment of writing, a collaboration between other real-world data sources and the HF registry is not yet established for all external databases.

Statistical analysis

<u>Clinical effectiveness</u>

Descriptives are presented for all variables of the HF registry and telemedicine components. We will present the number of days without unplanned hospitalization for HF during 365 days after baseline,

For peer review only - http://bmjopen.bmj.com/site/about/guidelines.xhtml

the average duration of unplanned HF-related hospital stays, the number of deaths, and time until death.

Preparation of the statistical model

As a first step, we describe how the overall effect of offering any type of telemedicine versus not offering telemedicine will be estimated. This analysis will subsequently be used for a comparison of telemedicine effects on days alive without unplanned HF-related hospitalization during one year across subgroups.

The cohort is restricted to individuals with newly diagnosed HF, that is, at most three months prior to baseline. The exposure contrast is defined as offering any type of telemedicine after first presentation with HF at the hospital outpatient clinic versus patients not offering telemedicine. The telemedicine status for the entire follow-up is defined by the 6-months after baseline measurement of telemedicine, used as an observational analog of an “intention to treat” analysis in which stopping of telemedicine is disregarded.

Because telemedicine administration is dependent on patient characteristics, confounding by indication will be accounted for. Based on clinical consensus, the following patient-level confounding variables measured at baseline will be adjusted for: age, sex, SES, BMI, NYHA, LVEF, etiology of HF, systolic blood pressure, diastolic blood pressure, heart rhythm, heart rate, chronic respiratory disorders, OSAS, stroke, extra cardiac arterial vascular pathology, DM, hypertension, anemia, CKD, malignancy, heart rhythm disorders, depression, and thyroid disease (Table 3 and S1).

The primary outcome model is a linear mixed model with a random intercept for participating center. It is specified as follows. The outcome is measured in days and because the final measurement may be less or more than 365 days after baseline, we rescale the number of days of unplanned hospitalization to the number of days under follow-up to 365 days. The outcome is computed by subtracting days of unplanned hospitalization or death from 365. The earlier described confounding variables are added to the primary outcome model to adjust for confounding. Continuous covariates are modelled using splines. The coefficient for telemedicine in the primary outcome model represents the average treatment effect of offering any telemedicine within 3 months after HF diagnosis compared to not offering telemedicine, on days with unplanned HF-related hospitalization during a year in the population of newly diagnosed HF patients. Robust standard errors are estimated to compute confidence intervals.

For the secondary outcome functional status, a similar approach is taken to estimate the average treatment effect of offering any telemedicine within 3 months after HF diagnosis compared to not offering telemedicine on functional status after one year in the population of newly diagnosed HF patients. For this analysis, the outcome model is a multinomial regression model and baseline functional status is added as a covariate. The risk difference relative to the reference outcome category is assessed using the predicted outcomes of the primary outcome model under offering of telemedicine versus not offering to obtain the marginal risk difference.[46-48] Similarly, for the secondary outcomes health status and hrQoL, the outcome model is a probit regression model and baseline measurements are added as a covariate. Healthcare utilization will be assessed using a Poisson regression model, and all-cause mortality will be assessed using binary logistic regression.

We plan to perform the following additional analyses. First, we plan to perform the analysis with the start of follow up defined at the T1 measurement and outcome of unplanned hospitalization days

For peer review only - http://bmjopen.bmj.com/site/about/guidelines.xhtml

during 6 months. This is because telemedicine status can be misclassified when telemedicine is started shortly after baseline but is not registered in the data. Second, we plan to perform a per protocol analysis of telemedicine use if detailed information on starting and stopping of telemedicine is available through linkage. Finally, we plan to perform all analyses in the entire cohort, rather than the cohort restricted on HF diagnosis at most 3 months prior to T0.

RQ 1: effectiveness of telemedicine in patient subgroups

Research question 1 investigates the effectiveness of telemedicine across subgroups of HF patients. Patient-related subgroups were identified by a systematic literature review of RCTs of telemedicine: age, severity of HF (NYHA class at baseline), sex, SES, presence of depression, atrial fibrillation (AF), type of HF (HFpEF, HFmrEF, HFrEF) (Table 3).[27-31, 49-57] Stratified analyses of the primary outcome model are performed to estimate the above defined average treatment effect in each of the subgroups. Heterogeneity in telemedicine effect across age is assessed on a continuous scale, where an interaction between age and telemedicine status is added to the primary outcome model and expected number of days out of hospital across age ranges 50 to 90 years are predicted from this model under telemedicine offered versus not offered.

In an additional analysis, heterogeneity across time since diagnosis is explored for the entire cohort without restricting to patients with a maximum of three months since HF diagnosis at baseline (diagnosed ≤3 months before baseline compared to >3 months before baseline).

The secondary outcomes functional status, health status, and hrQoL are assessed using similar approaches but instead using a multinomial, probit, and probit regression model, respectively. Healthcare utilization will be assessed using a Poisson regression model, and all-cause mortality will be assessed using binary logistic regression.

RQ 2: effectiveness of different telemedicine components

Research question 2 investigates the effectiveness of the different forms of telemedicine intervention. We assess this question by performing three separate analyses in which intervention aspects are contrasted in the subset of participants that received telemedicine. The presence or absence of a telemedicine component is determined on a hospital level. Components of telemedicine that are contrasted are: presence versus absence of a service center, presence versus absence of an educational module, high-intensity versus low-intensity protocol (Table 2).

The population of interest are newly diagnosed patients with HF (maximum 3 months prior to baseline) who received telemedicine. Offering of telemedicine is measured at 6-months after baseline.

In addition to the set of confounding variables at the patient level used in the analysis above, confounding variables at the hospital level are considered because indication for telemedicine is expected to differ across hospitals. Included confounding variables are: academic or non-academic hospital, presence of a specialized heart center, presence of an outpatient ward, size of outpatient ward (number of patients/year), staffing of the HF outpatient clinic, and full-time equivalent of outpatient-ward staff.

For each of the three contrasts, the primary outcome model is fitted to estimate the average treatment effect of a component relative to the corresponding reference component on days of

For peer review only - http://bmjopen.bmj.com/site/about/guidelines.xhtml

unplanned hospitalisation during one year in the population of newly diagnosed HF patients that initiated telemedicine.

The secondary outcomes functional status, health status, and hrQoL are assessed using similar approaches but instead using a multinomial, probit, and probit regression model, respectively. Healthcare utilization and all-cause mortality will be assessed using a Poisson regression model, and binary logistic regression, respectively.

<u>Cost effectiveness</u>

Costs will be estimated by computing the average costs (using cost guideline provided by the National Health Care Institute) per individual based on the information collected by NHR and in the interviews with hospitals. Since 2023 Dutch hospitals can claim costs of telemedicine using Diagnosis Treatment Combination (DBCs) by the National Health Care Institute. These DBCs can be used to estimate the average costs.[45] A 95% credible interval (CE) will be computed using the percentiles of a Monte-Carlo bootstrap analysis with 5,000 resamplings. This average cost represents a sum of the costs of telemedicine use, inpatient days, days at ICU, HF related hospital procedures, and outpatient visits. In a secondary analysis, we will re-estimate average costs and 95% CE again using data enriched with VEKTIS data (if linking is available), meaning that costs of visits to the GP, pharmacy, and care at home are taken into account as well.

Linkage with CBS may be incomplete for several participants because of missing values or incomplete datasets (for example twins, different available time windows between registries of collected data). If the number of non-linked participants is below 10%, we will perform multiple imputation. If the number of non-linked participants is above 10%, we will perform HTA analysis on the complete subset.

Utility values will be estimated using the average hrQoL score collected in the HF registry and if needed from literature. The difference in average QoL score between the group that uses telemedicine versus the group that does not use telemedicine represents the difference in disutility between the groups under the assumption that the collected hrQoL is a good representation of the utility of the particular health state that individuals were in.

Quality Adjusted Life Years (QALYs) are estimated by multiplying the observed number of follow-up years by the corresponding hrQoL score. Subsequently, the Incremental Cost Effectiveness Ratio (ICER) is computed by taking the ratio between the difference of costs of the average patient in the telemedicine group and those not in the telemedicine group and the difference in QALY of both groups, thereby providing the cost per additional QALY gained. The 95% CEs are obtained using the Monte-Carlo percentile methods described above. Both deterministic and probabilistic sensitivity analyses will be performed to completely outline uncertainty on individual and combined parameters in the model.

In addition, willingness to pay curves will be drawn to draw impact of different threshold on cost-effectiveness outcomes.

RQ 3: cost-effectiveness of telemedicine in patient subgroups

For research question 3, costs, utility, QALYs, ICER, and willingness to pay curves will be computed to estimate the difference in cost-effectiveness between users of telemedicine and non-users of

For peer review only - http://bmjopen.bmj.com/site/about/guidelines.xhtml

telemedicine in general. Subsequently, the analyses will be repeated to estimate cost-effectiveness in subgroups, similar to the groups defined for research question 2 in the clinical effectiveness analysis.

RQ 4: cost-effectiveness of different telemedicine components

For research question 4, costs, utility, QALYs, ICER, and willingness to pay curves will be computed to estimate the difference in cost-effectiveness between components of telemedicine, similar to the comparisons defined for research question 3 in the clinical effectiveness analysis.

Sample size calculation

The primary focus in RELEASE-HF is on the clinical effectiveness of telemedicine in HF management. Hence, the sample size calculation is based on parameters relevant to this analysis. The sample size calculation was conducted in three steps: (1) computing required sample size for the main effect of telemedicine on the primary outcome, (2) taking into account that subgroup effects are of main interest and (3) anticipating the accrual rate.

For the first part, we assumed a mean increase in number of days spent alive without unplanned hospitalization of 6.4 (SD 21.1) days based on findings of the TIM-HF2 study.[52] This corresponds to a required sample size of 432 patients in total, based on a type I error probability of 5%, a type II error probability of 20%, and accounting for 20% drop out.

Such a sample size would allow for an overall estimate of the difference between telemedicine and no telemedicine in days spent alive without unplanned hospitalization in the overall patient population, however our interest is in heterogeneous intervention effects. Hence, for the second part, the sample size was inflated to estimate subgroup effects.[58, 59] To be able to detect interaction effects that are 50% to 60% of the size of the overall effect (i.e., increase of more than 3.2 days to 3.8 days spent outside the hospital due to the interaction given the main effect) with a power of 80%, the required sample size is inflated with a factor 12.[58] This results in a required sample size of 5,184 individuals.

Finally, from the HF registry pilot study (CHECK-HF), we know that in view of the estimated average number of patients per hospital the average proportion of included outpatient clinic patients with HF was above 80%.[60] Taking this accrual rate into account we would require the inclusion of 6,480 HF patients.

Patient and public involvement

Patient and public organizations were involved during grant application. Public organizations were involved in recruiting hospitals, and legal support and advice. Health care professional involvement in the study includes participating in an interview about telemedicine and motives about telemedicine choice and use. Patients with HF and patient organizations will be involved in formulating and prioritizing relevant research questions which are in line with the need of the patient and the aim of RELEASE-HF. Results will be disseminated with involvement of patient and public organizations.

For peer review only - http://bmjopen.bmj.com/site/about/guidelines.xhtml

ETHICS AND DISSEMINATION

Management and storage of data

RELEASE-HF is an observational, retrospective multicentre cohort study of prospectively collected data registered within the NHR. A waiver for informed consent for analysis with the data of the NHR data registry was obtained. Data collection and registration is performed by the participating centres in a secured online environment. Detailed information on the process of data acquisition, completeness, data quality and analysis of the NHR has been published previously.[37] To obtain reliable data, the NHR has an advanced, certified data quality control system in place to ensure completeness and quality of data.[44]

Participants of the interview study will sign informed consent. Data will be stored in the secured environment at the UMC Utrecht, the Netherlands. Data will be pseudonymized on participant and setting level. A secured identification log will be used, only accessible by the main researcher of RELEASE-HF. Because the interview data will be linked to the HF registry, pseudonymized data will also be stored at the Medical Informatics Department of University Medical Centers Amsterdam (the Netherlands).

Storage of linked datasets (HF registry with other real-world data sources) will be part of the infrastructure established by the Heart4Data consortium.[35] RELEASE-HF study will follow these principles.

The Medical Ethics Committee of UMC Utrecht (the Netherlands) reviewed the study protocol and confirmed that the study does not fall under the scope of the Medical Research Involving Human Subjects Act (WMO). RELEASE-HF complies with the rules of the General Data Protection Regulation (GDPR).

Dissemination

The results will be published in peer-reviewed journals and presented at (inter)national conferences as deemed relevant for HF and telemedicine. The HF registry data underlying this article were provided by the NHR by the permission of the participating hospitals. Data are available upon reasonable request to the corresponding author and with permission of the NHR. The hospital specific telemedicine characteristics, which are added to the HF registry data will also only be available upon a reasonable request to the corresponding author and with permission of the participating hospitals.

For peer review only - http://bmjopen.bmj.com/site/about/guidelines.xhtml

REFERENCES

1. James SL, Abate D, Abate KH, et al. Global, regional, and national incidence, prevalence, and years lived with disability for 354 Diseases and Injuries for 195 countries and territories, 1990-2017: A systematic analysis for the Global Burden of Disease Study 2017. *The Lancet* 2018;392(10159):1789-858. doi: 10.1016/S0140-6736(18)32279-7.
2. McDonagh TA, Metra M, Adamo M, et al. 2021 ESC Guidelines for the diagnosis and treatment of acute and chronic heart failure. *Eur Heart J* 2021;42(36):3599-726. doi: 10.1093/eurheartj/ehab368.
3. Groenewegen A, Rutten FH, Mosterd A, et al. Epidemiology of heart failure. *Eur J Heart Fail* 2020;22(8):1342-56. doi: 10.1002/ejhf.1858.
4. Cook C, Cole G, Asaria P, et al. The annual global economic burden of heart failure. *Int J Cardiol* 2014;171(3):368-76. doi: 10.1016/j.ijcard.2013.12.028.
5. Tsao CW, Lyass A, Enserro D, et al. Temporal Trends in the Incidence of and Mortality Associated With Heart Failure With Preserved and Reduced Ejection Fraction. *JACC Heart Fail* 2018;6(8):678-85. doi: 10.1016/j.jchf.2018.03.006.
6. Volksgezondheid. Hartfalen [cited 2022 April 25]. Available from: https://www.vzinfo.nl/hartfalen accessed April 25 2022.
7. Anker SD, Koehler F, Abraham WT. Telemedicine and remote management of patients with heart failure. *Lancet* 2011;378(9792):731-9. doi: 10.1016/S0140-6736(11)61229-4.
8. Gensini GF, Alderighi C, Rasoini R, et al. Value of Telemonitoring and Telemedicine in Heart Failure Management. *Card Fail Rev* 2017;3(2):116-21. doi: 10.15420/cfr.2017:6:2.
9. Rosen D, McCall JD, Primack BA. Telehealth Protocol to Prevent Readmission Among High-Risk Patients With Congestive Heart Failure. *Am J Med* 2017;130(11):1326-30. doi: 10.1016/j.amjmed.2017.07.007.
10. Wagenaar KP, Broekhuizen BDL, Jaarsma T, et al. Effectiveness of the European Society of Cardiology/Heart Failure Association website 'heartfailurematters.org' and an e-health adjusted care pathway in patients with stable heart failure: results of the 'e-Vita HF' randomized controlled trial. *Eur J Heart Fail* 2019;21(2):238-46. doi: 10.1002/ejhf.1354.
11. Ross J, Stevenson F, Lau R, et al. Factors that influence the implementation of e-health: a systematic review of systematic reviews (an update). *Implement Sci* 2016;11(1):146. doi: 10.1186/s13012-016-0510-7.
12. Ayuso P. REPORT on the eHealth Action Plan 2012-2020 – Innovative healthcare for the 21st century, 2013.
13. Colucci M, Baldo V, Baldovin T, et al. A "matter of communication": A new classification to compare and evaluate telehealth and telemedicine interventions and understand their effectiveness as a communication process. *Health Informatics J* 2019;25(2):446-60. doi: 10.1177/1460458217747109.
14. Ding H, Chen SH, Edwards I, et al. Effects of Different Telemonitoring Strategies on Chronic Heart Failure Care: Systematic Review and Subgroup Meta-Analysis. *J Med Internet Res* 2020;22(11):e20032. doi: 10.2196/20032.
15. Hafkamp FJ, Tio RA, Otterspoor LC, et al. Optimal effectiveness of heart failure management - an umbrella review of meta-analyses examining the effectiveness of interventions to reduce (re)hospitalizations in heart failure. *Heart Fail Rev* 2022 doi: 10.1007/s10741-021-10212-8.
16. Inglis SC, Clark RA, Dierckx R, et al. Structured telephone support or non-invasive telemonitoring for patients with heart failure. *Cochrane Database Syst Rev* 2015(10):CD007228. doi: 10.1002/14651858.CD007228.pub3.
17. Kotb A, Cameron C, Hsieh S, et al. Comparative effectiveness of different forms of telemedicine for individuals with heart failure (HF): a systematic review and network meta-analysis. *PLoS One* 2015;10(2):e0118681. doi: 10.1371/journal.pone.0118681.

For peer review only - http://bmjopen.bmj.com/site/about/guidelines.xhtml

18. Kruse CS, Soma M, Pulluri D, et al. The effectiveness of telemedicine in the management of chronic heart disease - a systematic review. *JRSM Open* 2017;8(3):2054270416681747. doi: 10.1177/2054270416681747.
19. Lin MH, Yuan WL, Huang TC, et al. Clinical effectiveness of telemedicine for chronic heart failure: a systematic review and meta-analysis. *J Investig Med* 2017;65(5):899-911. doi: 10.1136/jim-2016-000199.
20. Pandor A, Thokala P, Gomersall T, et al. Home telemonitoring or structured telephone support programmes after recent discharge in patients with heart failure: systematic review and economic evaluation. *Health Technol Assess* 2013;17(32):1-207, v-vi. doi: 10.3310/hta17320.
21. Peters GM, Kooij L, Lenferink A, et al. The Effect of Telehealth on Hospital Services Use: Systematic Review and Meta-analysis. *J Med Internet Res* 2021;23(9):e25195. doi: 10.2196/25195.
22. Yun JE, Park JE, Park HY, et al. Comparative Effectiveness of Telemonitoring Versus Usual Care for Heart Failure: A Systematic Review and Meta-analysis. *J Card Fail* 2018;24(1):19-28. doi: 10.1016/j.cardfail.2017.09.006.
23. Driscoll A, Gao L, Watts JJ. Clinical effectiveness and cost-effectiveness of ambulatory heart failure nurse-led services: an integrated review. *BMC Cardiovasc Disord* 2022;22(1):64. doi: 10.1186/s12872-022-02509-9.
24. Pekmezaris R, Tortez L, Williams M, et al. Home telemonitoring in heart failure: a systematic review and meta-analysis. *Health Affairs* 2018;37(12):1983-89.
25. Brugts JJ, Radhoe SP, Clephas PRD, et al. Remote haemodynamic monitoring of pulmonary artery pressures in patients with chronic heart failure (MONITOR-HF): a randomised clinical trial. *Lancet* 2023;401(10394):2113-23. doi: 10.1016/S0140-6736(23)00923-6.
26. Scholte NT, Gürgöze MT, Aydin D, et al. Telemonitoring for heart failure: a meta-analysis. *European Heart Journal* 2023:ehad280.
27. Boyne JJ, Vrijhoef HJ, Crijns HJ, et al. Tailored telemonitoring in patients with heart failure: results of a multicentre randomized controlled trial. *Eur J Heart Fail* 2012;14(7):791-801. doi: 10.1093/eurjhf/hfs058.
28. Koehler F, Winkler S, Schieber M, et al. Telemedicine in heart failure: pre-specified and exploratory subgroup analyses from the TIM-HF trial. *Int J Cardiol* 2012;161(3):143-50. doi: 10.1016/j.ijcard.2011.09.007.
29. Koehler J, Stengel A, Hofmann T, et al. Telemonitoring in patients with chronic heart failure and moderate depressed symptoms: results of the Telemedical Interventional Monitoring in Heart Failure (TIM-HF) study. *Eur J Heart Fail* 2021;23(1):186-94. doi: 10.1002/ejhf.2025.
30. Galinier M, Roubille F, Berdague P, et al. Telemonitoring versus standard care in heart failure: a randomised multicentre trial. *Eur J Heart Fail* 2020;22(6):985-94. doi: 10.1002/ejhf.1906.
31. Krzesinski P, Jankowska EA, Siebert J, et al. Effects of an outpatient intervention comprising nurse-led non-invasive assessments, telemedicine support and remote cardiologists' decisions in patients with heart failure (AMULET study): a randomised controlled trial. *Eur J Heart Fail* 2022;24(3):565-77. doi: 10.1002/ejhf.2358.
32. Auener SL, Remers TEP, van Dulmen SA, et al. The Effect of Noninvasive Telemonitoring for Chronic Heart Failure on Health Care Utilization: Systematic Review. *J Med Internet Res* 2021;23(9):e26744. doi: 10.2196/26744.
33. Asselbergs FW, Baars F, Boyne JJJ, et al. Samenwerkingsafspraken en kwaliteitscriteria bij invoeren telebegeleiding bij hartfalen in Nederland 2016.
34. ClinicalTrials.gov. Identifier NCT05654961, REsponsible roLl-out of E-heAlth Through Systematic Evaluation - Heart Failure Study (RELEASE-HF) National Library of Medicine (US): Clinicaltrials.gov; 2022 [cited 2022 December 16]. Available from: https://classic.clinicaltrials.gov/ct2/show/NCT05654961?term=RELEASE-HF&draw=2&rank=1 accessed July 10 2023.
35. DCVA. Heart4Data [cited 2023 30 January]. Available from: https://dcvalliance.nl/heart4data accessed 30 January 2023.

For peer review only - http://bmjopen.bmj.com/site/about/guidelines.xhtml

36. NHR. Registratie [cited 2023 May 10]. Available from: https://nederlandsehartregistratie.nl/registratie/ accessed April 25 2022.
37. Timmermans MJ, Houterman S, Daeter ED, et al. Using real-world data to monitor and improve quality of care in coronary artery disease: results from the Netherlands Heart Registration. *Netherlands Heart Journal* 2022:1-9.
38. NHR. Handboek Nederlandse Hart Registratie | Hartfalen. 22.1.3 ed: Nederlandse Hart Registratie, 2022.
39. Aaronson NK, Muller M, Cohen PD, et al. Translation, validation, and norming of the Dutch language version of the SF-36 Health Survey in community and chronic disease populations. *J Clin Epidemiol* 1998;51(11):1055-68. doi: 10.1016/s0895-4356(98)00097-3.
40. Brazier JE, Roberts J. The estimation of a preference-based measure of health from the SF-12. *Med Care* 2004;42(9):851-9. doi: 10.1097/01.mlr.0000135827.18610.0d.
41. Bashshur R, Shannon G, Krupinski E, et al. The taxonomy of telemedicine. *Telemed J E Health* 2011;17(6):484-94. doi: 10.1089/tmj.2011.0103.
42. Edmunds M, Tuckson R, Lewis J, et al. An Emergent Research and Policy Framework for Telehealth. *EGEMS (Wash DC)* 2017;5(2):1303. doi: 10.13063/2327-9214.1303.
43. Tulu B, Chatterjee S, Maheshwari M. Telemedicine taxonomy: a classification tool. *Telemed J E Health* 2007;13(3):349-58. doi: 10.1089/tmj.2006.0055.
44. Houterman S, van Dullemen A, Versteegh M, et al. Data quality and auditing within the Netherlands Heart Registration: using the PCI registry as an example. *Neth Heart J* 2023 doi: 10.1007/s12471-022-01752-1.
45. Nederland Z. Richtlijnen voor economische evaluatie [cited 2023 May 3]. Available from: https://www.zorginstituutnederland.nl/over-ons/werkwijzen-en-procedures/adviseren-over-en-verduidelijken-van-het-basispakket-aan-zorg/beoordeling-van-geneesmiddelen/richtlijnen-voor-economische-evaluatie2023.
46. Austin PC. Absolute risk reductions, relative risks, relative risk reductions, and numbers needed to treat can be obtained from a logistic regression model. *J Clin Epidemiol* 2010;63(1):2-6. doi: 10.1016/j.jclinepi.2008.11.004.
47. Greenland S. Model-based estimation of relative risks and other epidemiologic measures in studies of common outcomes and in case-control studies. *Am J Epidemiol* 2004;160(4):301-5. doi: 10.1093/aje/kwh221.
48. Localio AR, Margolis DJ, Berlin JA. Relative risks and confidence intervals were easily computed indirectly from multivariable logistic regression. *J Clin Epidemiol* 2007;60(9):874-82. doi: 10.1016/j.jclinepi.2006.12.001.
49. Chaudhry SI, Mattera JA, Curtis JP, et al. Telemonitoring in patients with heart failure. *N Engl J Med* 2010;363(24):2301-9. doi: 10.1056/NEJMoa1010029.
50. Cleland JG, Louis AA, Rigby AS, et al. Noninvasive home telemonitoring for patients with heart failure at high risk of recurrent admission and death: the Trans-European Network-Home-Care Management System (TEN-HMS) study. *J Am Coll Cardiol* 2005;45(10):1654-64. doi: 10.1016/j.jacc.2005.01.050.
51. Dendale P, De Keulenaer G, Troisfontaines P, et al. Effect of a telemonitoring-facilitated collaboration between general practitioner and heart failure clinic on mortality and rehospitalization rates in severe heart failure: the TEMA-HF 1 (TElemonitoring in the MAnagement of Heart Failure) study. *Eur J Heart Fail* 2012;14(3):333-40. doi: 10.1093/eurjhf/hfr144.
52. Koehler F, Koehler K, Deckwart O, et al. Efficacy of telemedical interventional management in patients with heart failure (TIM-HF2): a randomised, controlled, parallel-group, unmasked trial. *The Lancet* 2018;392(10152):1047-57.
53. Lynga P, Persson H, Hagg-Martinell A, et al. Weight monitoring in patients with severe heart failure (WISH). A randomized controlled trial. *Eur J Heart Fail* 2012;14(4):438-44. doi: 10.1093/eurjhf/hfs023.

For peer review only - http://bmjopen.bmj.com/site/about/guidelines.xhtml

54. Ong MK, Romano PS, Edgington S, et al. Effectiveness of Remote Patient Monitoring After Discharge of Hospitalized Patients With Heart Failure: The Better Effectiveness After Transition -- Heart Failure (BEAT-HF) Randomized Clinical Trial. *JAMA Intern Med* 2016;176(3):310-8. doi: 10.1001/jamainternmed.2015.7712.
55. Piotrowicz E, Pencina MJ, Opolski G, et al. Effects of a 9-Week Hybrid Comprehensive Telerehabilitation Program on Long-term Outcomes in Patients With Heart Failure: The Telerehabilitation in Heart Failure Patients (TELEREH-HF) Randomized Clinical Trial. *JAMA Cardiol* 2020;5(3):300-08. doi: 10.1001/jamacardio.2019.5006.
56. Szalewska D, Glowczynska R, Piotrowicz R, et al. An aetiology-based subanalysis of the Telerehabilitation in Heart Failure Patients (TELEREH-HF) trial. *ESC Heart Fail* 2021;8(2):1263-73. doi: 10.1002/ehf2.13189.
57. Pekmezaris R, Nouryan CN, Schwartz R, et al. A randomized controlled trial comparing telehealth self-management to standard outpatient management in underserved black and Hispanic patients living with heart failure. *Telemedicine and e-Health* 2019;25(10):917-25.
58. Brookes ST, Whitely E, Egger M, et al. Subgroup analyses in randomized trials: risks of subgroup-specific analyses; power and sample size for the interaction test. *J Clin Epidemiol* 2004;57(3):229-36. doi: 10.1016/j.jclinepi.2003.08.009.
59. Burke JF, Sussman JB, Kent DM, et al. Three simple rules to ensure reasonably credible subgroup analyses. *BMJ* 2015;351:h5651. doi: 10.1136/bmj.h5651.
60. Brugts JJ, Linssen GCM, Hoes AW, et al. Real-world heart failure management in 10,910 patients with chronic heart failure in the Netherlands : Design and rationale of the Chronic Heart failure ESC guideline-based Cardiology practice Quality project (CHECK-HF) registry. *Neth Heart J* 2018;26(5):272-79. doi: 10.1007/s12471-018-1103-7.

For peer review only

Acknowledgements

We would like to acknowledge all the study participants, cardiologists and nurses, from all participating hospitals for setting up the RELEASE-HF study and data collection in their hospital.

Study participants RELEASE-HF

John Younge, Marco van Gent (Albert Schweitzer Hospital); Jeroen Schaap (Amphia Hospital); M Louis Handoko (Amsterdam University Medical Centers); Ayten Erol-Yilmaz (Bernhoven hospital); Frederique Hafkamp (Netherlands Heart Network (NHN); Anna Hospital, Catharina hospital, Elkerliek, Máxima medical center); Wouter W Jansen Klomp (Deventer Hospital); Jaco Houtgraaf (Diakonessenhuis); Eric Wierda (Dijklander Hospital); Balázs Szabo (Elisabeth-TweeSteden Hospital); Rudolf A de Boer, Jasper J Brugts (Erasmus Medical Center); Stefan Koudstaal (Groene Hart Hospital); Jan Willem Borleffs (Haga Hospital); Gerard CM Linssen (Hospital Group Twente, Almelo & Hengelo); Dirk H van Dalen (Jeroen Bosch Hospital); Hans Peter Brunner-La Rocca (Maastricht University Medical Center); Jan van Ramshorst (Noord-West Hospital); Loek van Heerebeek (Onze Lieve Vrouwe Gasthuis); Stijn Wouters (Pantein Hospital); Roland van Kimmenade (Radboud University Medical Center); Gerardus PJ van Hout, Marco C Post (St. Antonius Hospital); Lennaert Kleijn (Treant Hospital); Manon G van der Meer (University Medical Center Utrecht); Sandra Sanders-van Wijk (Zuyderland Medical Center).

Contributors

JE contributed to protocol conception and design, development of statistical analyses, obtained ethics approval, writing the protocol manuscript, and critical revision of the protocol manuscript. KL contributed to protocol conception and design, development of statistical analyses, provided statistical and methodological support, writing the statistical analyses paragraph, and critical revision of the protocol manuscript. HR contributed to protocol conception and design, obtaining funding, development of statistical analyses, provided statistical and methodological support, and critically revised the manuscript. ES contributed to the development of statistical analyses, provided statistical and methodological support, and critically revised the manuscript. GF contributed to protocol conception and design, obtaining funding, writing the economic evaluation paragraph, and critically revised the manuscript. LD contributed to protocol conception for data collection combined with the use of the HF registry data, and critically revised the manuscript. JWB, DHD, AEY, MLH, GPH, JH, WJK, SK, GCL, MGM, MCP, SSW, EW, CWW, JS, JJB assisted with the data collection and critically revised the manuscript. TJ, JS, JJB, FHR, RAB contributed to protocol conception and design, obtaining funding, and critically revised the manuscript. FWA and JCT contributed to protocol conception and design, secured funding for the project, and critically revised the manuscript. Contributed equally to this paper. All authors approved the final version of the manuscript.

Funding

This study is supported by The Netherlands Organization for Health Research and Development (ZonMw grant 852002141). Additional support has been received from the Dutch CardioVascular Alliance (DCVA).

For peer review only - http://bmjopen.bmj.com/site/about/guidelines.xhtml

Competing interests

Dr. De Boer has received research grants and/or fees from AstraZeneca, Abbott, Boehringer Ingelheim, Cardior Pharmaceuticals GmbH, Ionis Pharmaceuticals, Inc., Novo Nordisk, and Roche; and has had speaker engagements with Abbott, AstraZeneca, Bayer, Bristol Myers Squibb, Novartis, and Roche.

Dr. Borleffs has received speaker fees from AstraZeneca, Boehringer Ingelheim, Novartis.

Dr. Handoko is supported by the Dutch Heart Foundation (dr. E. Dekker Senior Clinical Scientist Grant 2020T058) and CVON (2020B008 RECONNEXT). MLH received an investigator-initiated research grant of Vifor Pharma, an educational grant of Boehringer Ingelheim and Novartis, and speaker/consultancy fees of Abbott, AstraZeneca, Bayer, Boehringer Ingelheim, MSD, Novartis, Sankyo, Dayichi, Quin, Vifor Pharma; all not related to this study.

Dr. Brugts received an independent research grant from Abbott for ISS paid to institution, and has had speaker engagements or advisory boards to Astra Zeneca, Abbott, Bayer, Boehringer, Danchii-Sankyo, Novartis and Vifor in the past 5 years.

For peer review only

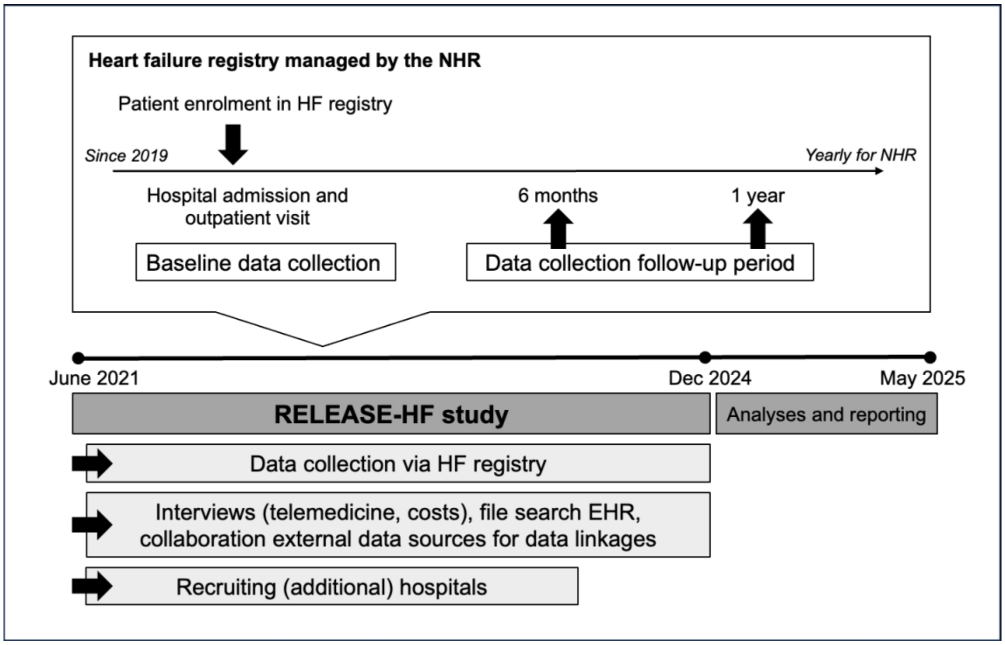

Figure 1 Overview time plan RELEASE-HF study.
HF: Heart Failure; RELEASE-HF: REsponsible roLl-out of E-heAlth through Systematic Evaluation – Heart Failure; NHR: Netherlands Heart Registration; EHR: Electronic Health Record.

120x77mm (300 x 300 DPI)

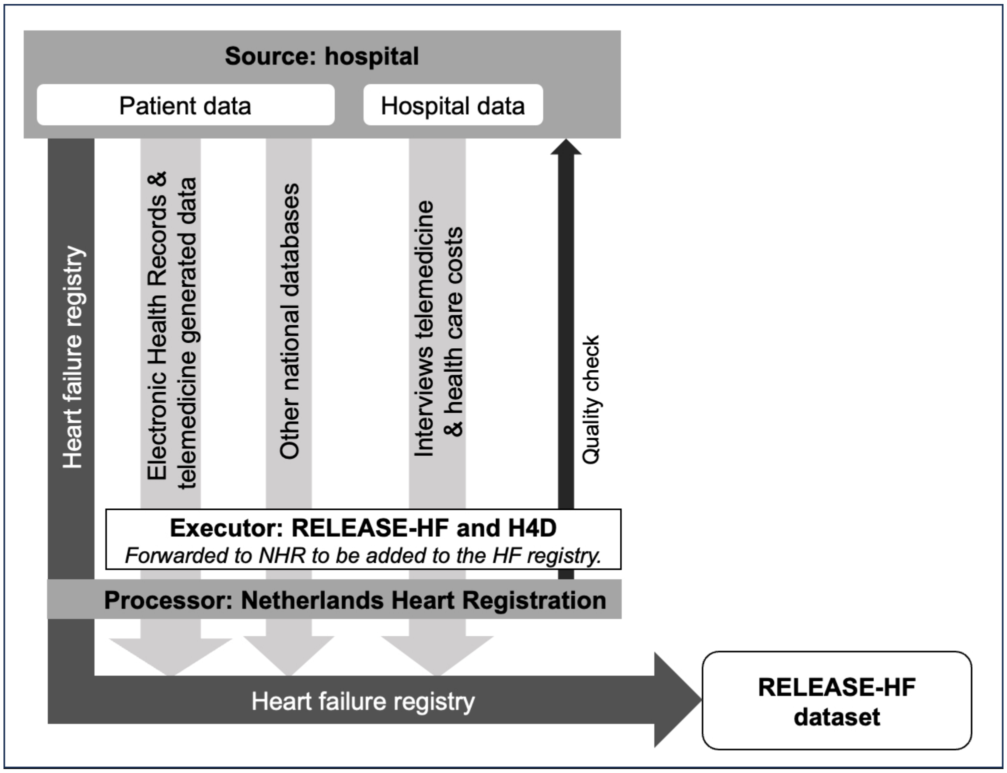

Figure 2 Data sources and flow to set up the final RELEASE-HF dataset. *RELEASE-HF: REsponsible roLl-out of E-heAlth through Systematic Evaluation – Heart Failure; H4D: Heart for Data Consortium; HF: Heart Failure.*

108x83mm (300 x 300 DPI)

Table S1 Variables of the heart failure registry

NOTE: Based on the dynamic data dictionary version 22.1.4 September 9, 2022. Definitions of variables and included variables are subject to change, since data dictionary depends on the current HF guidelines and is compiled by a committee consisting of delegated cardiologists from Dutch hospitals.

	Variables	Answer possibilities	Baseline (T0)	6 months (T1)	12 months (T2)
Identifying variables	Diagnosis number *	Unique number set up by the hospital	X		
	Date of diagnosis *	Date (DD-MM-YYYY)	X		
	Patient number*	Identification number in the hospital	X		
	Maiden name*	Name	X		
	Surname*	Name	X		
	Date of birth[$]	Date (DD-MM-YYYY)	X		
	Sex	Male<br>Female	X		
	Zip code*	1000 AA – 9999 ZZ= the Netherlands<br>0000= Abroad<br>-1= Unknown	X		
Diagnostic variables	Setting of inclusion	10= Outpatient clinic<br>20= Hospital, novo patient<br>30= Hospital, acute/chronic HF patient<br>-1 = Unknown	X		
	Previous HF diagnosis	0= No<br>1= Yes<br>-1= Unknown	X		
	Date of previous diagnosis (if applicable)	Date (DD-MM-YYYY)	X		
	Location diagnosis HF	10= Not earlier diagnosed with HF<br>10= Primary care<br>20= Secondary care<br>30= Tertiary care<br>-1= Unknown	X		
	Type of HF	10= HFrEF<br>20= HFmrEF<br>30= HFpEF<br>-1= Unknown	X		
	Etiology HFrEF	0= No HFrEF<br>10= Ischemic cardiomyopathy<br>20= Non-ischemic, hypertensive cardiomyopathy<br>50= Non-ischemic, arrhythmia/tachycardia mediated<br>60= Non-ischemic, hypertrophic cardiomyopathy<br>70= Non-ischemic, dilated cardiomyopathy (eci)<br>80= Non-ischemic, inflammation/infection (i.e., myocarditis)<br>90= Nonischemic, restrictive cardiomyopathy and/or accumulation<br>111= Nonischemic, familiar cq. genetic cardiomyopathy with proven DNA mutation	X		

For peer review only - http://bmjopen.bmj.com/site/about/guidelines.xhtml

		120= Non-ischemic, pericardial<br>135= Non-ischemic, toxic cardiomyopathy due to alcohol/drugs<br>136= Non-ischemic, toxic cardiomyopathy due to chemotherapy/radiation<br>139= Non-ischemic, toxic cardiomyopathy due to other<br>140= Non-ischemic, eci or (as yet) undetermined<br>900= Other (i.e., valve disease, metabolic, peripartum)<br>-1= Unknown			
	Etiology HFpEF	0= No HFpEF<br>10= Secondary HFpEF<br>20= Isolated hypertensive heart disease<br>30= Genetic HCM<br>40= Amyloidosis<br>50= Sarcoidosis<br>60= Hemochromatosis<br>70= Immune/inflammatory<br>80= Aortic valve stenosis<br>90= Pericardial diseases<br>900= Other<br>-1= Unknown	X		
**Patient characteristics**	***Identifying variables***				
	Follow-up moment	0 = T0<br>6 = T1<br>12 = T2<br>24 = T3<br>36 = T4<br>48 = T5	X	X	X
	Comorbidities				
	Hypertension	0= No<br>1= Yes<br>-1= Unknown	X	X	X
	Cardiovascular accident	0= No<br>1= Yes<br>-1= Unknown	X	X	X
	Extracardiac arterial vascular pathology	0= No<br>1= Yes<br>-1= Unknown	X	X	X
	Chronic respiratory disease	0= No<br>1= Yes<br>-1= Unknown	X	X	X
	Obstructive Sleep Apnea Syndrome	0= No<br>1= Yes<br>-1= Unknown	X	X	X
	Diabetes Mellitus	0= No<br>1= Diabetes, treatment unknown<br>2= Diabetes, no treatment<br>10= Diabetes, diet<br>20= Diabetes, oral medication<br>30= Diabetes, insulin<br>90= Diabetes, other<br>-1= Unknown	X	X	X
	Thyroid disorder	0= No<br>10= Yes, hypothyroidism<br>20= Yes, hyperthyroidism	X	X	X

For peer review only

		-1= Unknown			
	Malignancy	0= No<br>10= Yes, in the past<br>20= Yes, currently<br>-1= Unknown	X	X	X
	Measurements				
	Length	Measurement in centimeters	X	X	X
	Weight	Measurement in kilogram	X	X	X
	Systolic blood pressure	Measurement in mmHg	X	X	X
	Diastolic blood pressure	Measurement in mmHg	X	X	X
	Heart rhythm	10= Sinus rhythm<br>20= Atrium fibrillation<br>30= Atrial pacing<br>90= Other<br>-1= Unknown	X	X	X
	Heartrate	Measurement in beats per minute	X	X	X
	Atrial heart rhythm disorder	0= No<br>1= Yes<br>-1= Unknown	X	X	X
	Left bundle branch block	0= No<br>10= Yes, own heart's electrical conduction<br>20= Yes, pacing rhythm<br>-1= Unknown	X	X	X
	QRS complex duration	Measurement in milliseconds	X	X	X
	Left ventricle ejection fraction, NYHA classification and laboratory parameters				
	Left ventricle ejection fraction	Measurement in percentages	X	X	X
	NYHA class	1= Class I<br>2= Class II<br>3= Class III<br>4= Class IV<br>-1= Unknown	X	X	X
	NT-proBNP	1 t/m 8000= Value in pmol/l<br>0= No NT-proBNP measurement<br>-1= Unknown	X	X	X
	Date measurement NT-proBNP$	Date (DD-MM-YYYY)	X	X	X
	Serum creatinine	Value in µmol/l	X	X	X
	Iron deficiency	1= No<br>2= Yes, absolute ferritin < 100 µg/L<br>3= Yes, relative ferritin 100-300 µg/L with transferrin saturation < 20%<br>-1= Unknown	X	X	X
	Anemia	0= No<br>1= Yes<br>-1= Unknown	X	X	X
**Medication**	***Medication with target dose***				
	Type ACE inhibitor	0= No ACE inhibitor prescribed at Tn<br>10= Captopril (td in ESC 2021 = 150 mg)<br>20= Enalapril (td in ESC 2021= 20-40 mg)<br>3-= Lisinopril (td in ESC 2021 = 20-35 mg)<br>40= Ramipril (td in ESC 2021 = 10 mg)<br>50= Perindopril (td in ESC 2021 = 8 mg)	(X)	X	X

For peer review only - http://bmjopen.bmj.com/site/about/guidelines.xhtml

	60= Quinapril (td in ESC 2021 = 20 mg)<br>70= Fosinopril (td in ESC 2021 = 20-40 mg)<br>90= Other<br>-1= Unknown			
Target dose ACE inhibitor	0= No ACE inhibitor prescribed at Tn<br>1= No, target dose in ESC 2021 not reached due to contraindication or otherwise<br>2= No, target dose in ESC 2021 not reached due to titration phase<br>10= Yes, target dose in ESC 2021 reached or maximally tolerable for this patient<br>-1= Unknown	**(X)**	**X**	**X**
Type ARB	0= No ARB prescribed at Tn<br>10= Candesartan (td in ESC 2021 = 32 mg)<br>20= Losartan (td in ESC 2021 = 150 mg)<br>30= Valsartan (td in ESC 2021 = 320 mg)<br>40= Telmisartan (td in ESC 2021 = 80 mg)<br>50= Irbesartan (td in ESC 2021 = 300 mg)<br>90= Other<br>-1= Unknown	**(X)**	**X**	**X**
Target dose ARB	0= No ARB prescribed at Tn<br>1= No, target dose in ESC 2021 not reached due to contraindication or otherwise<br>2= No, target dose in ESC 2021 not reached due to titration phase<br>10= Yes, target dose in ESC 2021 reached or maximally tolerable for this patient<br>-1= Unknown	**(X)**	**X**	**X**
ARNI	0= No<br>1= Yes (td in ESC 2021 = 194-206 mg)<br>-1= Unknown	**(X)**	**X**	**X**
Target dose ARNI	0= No ARNI prescribed at Tn<br>1= No, target dose in ESC 2021 not reached due to contraindication or otherwise<br>2= No, target dose in ESC 2021 not reached due to titration phase<br>10= Yes, target dose in ESC 2021 reached or maximally tolerable for this patient<br>-1= Unknown	**(X)**	**X**	**X**
Type beta-blocker	0 = No beta blocker prescribed at Tn<br>10= Bisoprolol (td in ESC 2021 = 10 mg)<br>20= Metoprolol (td in ESC 2021 = 200 mg)<br>30= Carvedilol (td in ESC 2021 = 50 mg)	**(X)**	**X**	**X**

For peer review only - http://bmjopen.bmj.com/site/about/guidelines.xhtml

	40= Nebivolol (td in ESC 2021 = 10 mg)<br>50= Atenolol (td in ESC 2021 = 100 mg)<br>60= Pindolol (td in ESC 2021 = 20 mg)<br>70= Propranolol (td in ESC 2021 = 160 mg)<br>90= Other<br>-1= Unknown			
Target dose beta-blocker	0= No beta blocker prescribed at Tn<br>1= No, target dose in ESC 2021 not reached due to contraindication or otherwise<br>2= No, target dose in ESC 2021 not reached due to titration phase<br>10= Yes, target dose in ESC 2021 reached or maximally tolerable for this patient<br>-1= Unknown	**(X)**	**X**	**X**
Type MRA	0= No MRA prescribed at Tn<br>10= Eplerenone (td in ESC 2021= 50 mg)<br>20= Spironolactone (td in ESC 2021= 50 mg)<br>90= Other<br>-1= Unknown	**(X)**	**X**	**X**
Target dose MRA	0= No MRA prescribed at Tn<br>1= No, target dose in ESC 2021 not reached due to contraindication or otherwise<br>2= No, target dose in ESC 2021 not reached due to titration phase<br>10= Yes, target dose in ESC 2021 reached or maximally tolerable for this patient<br>-1= Unknown	**(X)**	**X**	**X**
Type SGLT2 inhibitor	0= No SGLT2 inhibitor prescribed at Tn<br>10= Canagliflozin (td in ESC 2021 = 10 mg)<br>20= Dapagliflozin (td in ESC 2021 = 10 mg)<br>30= Empagliflozin (td in ESC 2021 = 10 mg)<br>90= Other<br>-1= Unknown	**(X)**	**X**	**X**
Target dose SGLT2 inhibitor	0= No SGLT2 inhibitor prescribed at Tn<br>1= No, target dose in ESC 2021 not reached due to contraindication or otherwise<br>2= No, target dose in ESC 2021 not reached due to titration phase<br>10= Yes, target dose in ESC 2021 reached or maximally tolerable for this patient<br>-1= Unknown	**(X)**	**X**	**X**
Ivabradine	0= No<br>1= Yes	**(X)**	**X**	**X**

For peer review only - http://bmjopen.bmj.com/site/about/guidelines.xhtml

		-1= Unknown			
	Target dose Ivabradine	0= No ivabradine prescribed at Tn<br>1= No, target dose in ESC 2021 not reached due to contraindication or otherwise<br>2= No, target dose in ESC 2021 not reached due to titration phase<br>10= Yes, target dose in ESC 2021 reached or maximally tolerable for this patient<br>-1= Unknown	**(X)**	**X**	**X**
	Additional medication				
	Diuretic	0= No<br>1= Yes (target dose in ESC 2021 = 15mg)<br>-1= Unknown	**(X)**	**X**	**X**
	Vericiguat	0= No<br>1= Yes<br>-1= Unknown	**(X)**	**X**	**X**
	Digoxin	0= No<br>1= Yes<br>-1= Unknown	**(X)**	**X**	**X**
	Amiodarone	0= No<br>1= Yes<br>-1= Unknown	**(X)**	**X**	**X**
	Sotalol	0= No<br>1= Yes<br>-1= Unknown	**(X)**	**X**	**X**
	Hydralazine / Isosorbide dinitrate	0= No<br>1= Yes<br>-1= Unknown	**(X)**	**X**	**X**
**Outcome variables**	***Cardiac intervention and implantable***				
	Pacemaker – VVI	0= No<br>1= Yes<br>-1= Unknown	**(X)**	**X**	**X**
	Pacemaker – VVI date$	Date (DD-MM-YYYY)	**(X)**	**X**	**X**
	Pacemaker – DDD	0= No<br>1= Yes<br>-1= Unknown	**(X)**	**X**	**X**
	Pacemaker – DDD date$	Date (DD-MM-YYYY)	**(X)**	**X**	**X**
	CRT-P	0= No<br>1= Yes<br>-1= Unknown	**(X)**	**X**	**X**
	CRT-P date$	Date (DD-MM-YYYY)	**(X)**	**X**	**X**
	CRT-D	0= No<br>1= No, because of medical condition<br>2= No, because of patients' wish<br>10= Yes<br>-1= Unknown	**(X)**	**X**	**X**
	CRT-D date$	Date (DD-MM-YYYY)	**(X)**	**X**	**X**
	ICD – VVI	0= No<br>1= No, because of medical condition<br>2= No, because of patients' wish<br>10= Yes<br>-1= Unknown	**(X)**	**X**	**X**
	ICD – VVI date$	Date (DD-MM-YYYY)	**(X)**	**X**	**X**
	ICD – DDD	0= No<br>1= No, because of medical condition<br>2= No, because of patients' wish	**(X)**	**X**	**X**

For peer review only

		10= Yes<br>-1= Unknown			
	ICD – DDD date$	Date (DD-MM-YYYY)	**(X)**	**X**	**X**
	PCI	0= No<br>1= Yes<br>-1= Unknown	**(X)**	**X**	**X**
	PCI date$	Date (DD-MM-YYYY)	**(X)**	**X**	**X**
	CABG	0= No<br>1= Yes<br>-1= Unknown	**(X)**	**X**	**X**
	CABG date$	Date (DD-MM-YYYY)	**(X)**	**X**	**X**
	LVAD	0= No<br>1= Yes<br>-1= Unknown	**(X)**	**X**	**X**
	LVAD date$	Date (DD-MM-YYYY)	**(X)**	**X**	**X**
	Heart transplantation	0= No<br>1= Yes<br>-1= Unknown	**(X)**	**X**	**X**
	Heart transplantation date$	Date (DD-MM-YYYY)	**(X)**	**X**	**X**
	Heart revalidation	0= No<br>1= Yes<br>-1= Unknown	**(X)**	**X**	**X**
	Telemonitoring	0= No telemonitoring<br>10= Telemonitoring by telephone<br>20= Telemonitoring, non-invasive based on traditional parameters (e.g., blood pressure, ECG)<br>30= Telemonitoring, using ICD based on HF parameters<br>40= Telemonitoring, invasive by sensors in the blood stream or heart<br>-1= Unknown	**(X)**	**X**	**X**
	Patient status and quality of life				
	Mortality	0= Alive<br>1= Deceased<br>-1= Unknown		**X**	**X**
	Date mortality$	Date (DD-MM-YYYY)		**X**	**X**
	Number of hospitalizations	Number		**X**	**X**
	Date hospitalization$	Date (DD-MM-YYYY)		**X**	**X**
	Number of hospitalization days	Number		**X**	**X**
	Location follow-up	0= No follow-up, because of mortality<br>10= Patient stays in secondary care, outpatient HF clinic<br>20= Patient stays in tertiary care, outpatient HF clinic<br>30= Patient transferred to primary care<br>40= Patient transferred to the cardiology outpatient clinic<br>90= Other<br>-1= Unknown		**X**	**X**
	Type quality of life questionnaire	0= Not measured<br>10= SF12-2<br>20= SF36-2<br>30= SF36-1<br>-1= Unknown	**X**	**X**	**X**
	Quality of life	Coding depends on questionnaire	**X**	**X**	**X**

*Variables are collected in the Heart failure registry, but the data is not distributed and available for research.

For peer review only

$All variables which consist of a date will be transformed to number of days since diagnosed with HF. The actual date is not available in research.

HF: Heart Failure; HFrEF: Heart Failure with reduced Ejection Fraction; HFmrEF: Heart Failure with mildly reduced Ejection Fraction; HFpEF: Heart failure with preserved Ejection Fraction; NYHA: New York Heart Association; eci: e causa ignota (unknown cause); NT-proBNP: N-Terminal pro B-type Natriuretic Peptide; ACE: Angiotensin Converting Enzyme; td: target dose; ARB: Angiotensin Receptor Blocker; ARNI: Angiotensin Receptor-Neprilysin Inhibitor; MRA: Mineralocorticoid Receptor Antagonists; SGLT2: Sodium-glucose co-transporter-2; VVI: Ventricular pacing; DDD: dual-chamber antibradycardia pacing; CRT-P: Cardiac Resynchronization Therapy with a Pacemaker; CRT-D: Cardiac Resynchronization Therapy with a pacemaker and an ICD; ICD: Implantable Cardioverter-Defibrillator; PCI: Percutaneous Coronary Intervention; CABG: Coronary Artery Bypass Graft; LVAD: Left Ventricular Assist Device; ECG: Electro Cardio Gram; SF12/SF36: Short Form Health Survey with 12 questions or 36 questions.

For peer review only

BMJ Open

RELEASE-HF study protocol: An observational, registry-based study on the effectiveness of telemedicine in heart failure in the Netherlands

Journal:	*BMJ Open*
Manuscript ID	bmjopen-2023-078021.R1
Article Type:	Protocol
Date Submitted by the Author:	11-Oct-2023
Complete List of Authors:	van Eijk, Jorna; University Medical Centre Utrecht, General Practice & Nursing Science, Julius Center for Health Sciences and Primary Care<br>Luijken, Kim; University Medical Centre Utrecht, Epidemiology & Health Economics, Julius Center for Health Sciences and Primary Care<br>Jaarsma, Tiny; University Medical Centre Utrecht, General Practice & Nursing Science, Julius Center for Health Sciences and Primary Care<br>Reitsma, Johannes; University Medical Centre Utrecht, Epidemiology & Health Economics, Julius Center for Health Sciences and Primary Care<br>Schuit, Ewoud; University Medical Centre Utrecht, Epidemiology & Health Economics, Julius Center for Health Sciences and Primary Care<br>Frederix, Geert; Universitair Medisch Centrum Utrecht, Epidemiology & Health Economics, Julius Center for Health Sciences and Primary Care<br>Derks, Lineke; Netherlands Heart Registration<br>Schaap, Jeroen; Amphia Hospital, Cardiology; WCN, Dutch Network for Cardiovascular Research<br>Rutten, Frans; University Medical Centre Utrecht, General Practice & Nursing Science, Julius Center for Health Sciences and Primary Care<br>Brugts, Jasper; Erasmus Medical Center, Cardiology<br>de Boer, Rudolf; Erasmus Medical Center, Cardiology<br>Asselbergs, Folkert; Amsterdam University Medical Centres, Department of Cardiology; University College London, Health Data Research UK and Institute of Health Informatics<br>Trappenburg, Jaap; University Medical Centre Utrecht, The Healthcare Innovation Center, Julius Center for Health Sciences and Primary Care<br>investigators, RELEASE-HF ; University Medical Centre Utrecht
**Primary Subject Heading**:	Cardiovascular medicine
Secondary Subject Heading:	Patient-centred medicine, Health economics, Nursing
Keywords:	Heart failure < CARDIOLOGY, Telemedicine < BIOTECHNOLOGY & BIOINFORMATICS, REGISTRIES, HEALTH ECONOMICS, Protocols & guidelines < HEALTH SERVICES ADMINISTRATION & MANAGEMENT, Clinical Decision-Making

SCHOLARONE™
Manuscripts

RELEASE-HF study protocol: An observational, registry-based study on the effectiveness of telemedicine in heart failure in the Netherlands

Authors:

Jorna van Eijk, MSc RN[1]*
Kim Luijken, PhD[2]
Tiny Jaarsma, PhD RN[1]
Johannes B Reitsma, PhD[2]
Ewoud Schuit, PhD[2]
Geert WJ Frederix, PhD[2]
Lineke Derks, MSc[3]
Jeroen Schaap, MD PhD[4, 5]
Frans H Rutten, MD PHD[1]
Jasper J Brugts, MD PhD MSc[6]
Rudolf A de Boer, MD PhD[6]
Folkert W Asselbergs, MD PhD[7, 8]†
Jaap CA Trappenburg, PhD[9] †
RELEASE-HF INVESTIGATORS

RELEASE-HF INVESTIGATORS (group authorship/collaborator):

C Jan Willem Borleffs, MD PhD[10]
Dirk H van Dalen, MSc RN[11]
Ayten Erol-Yilmaz, MD PhD[12]
M Louis Handoko, MD PhD MSc[7, 13]
Gerardus PJ van Hout, MD[14, 15]
Jaco Houtgraaf, MD PhD[16]
Wouter W Jansen Klomp, MD MSc[17]
Stefan Koudstaal, MD PHD MSc FESC FHFA[18]
Gerard CM Linssen, MD PhD FESC FHFA[19]
Manon G van der Meer, MD PhD[15]
Marco C Post, MD, PhD[14, 15]
Sandra Sanders-van Wijk, MD PhD[20]
Eric Wierda, MD PhD[21]
Stijn CW Wouters, MD PhD[22]

For peer review only - http://bmjopen.bmj.com/site/about/guidelines.xhtml

1 Department of General Practice & Nursing Science, Julius Center for Health Sciences and Primary Care, University Medical Center Utrecht, Utrecht University, Utrecht, the Netherlands
2 Department of Epidemiology & Health Economics, Julius Center for Health Sciences and Primary Care, University Medical Center Utrecht, Utrecht University, Utrecht, The Netherlands
3 Netherlands Heart Registration, Utrecht, The Netherlands
4 Department of Cardiology, Amphia Hospital, Breda, The Netherlands
5 Dutch Network for Cardiovascular Research (WCN), Utrecht, The Netherlands
6 Department of Cardiology, Erasmus Medical Center, Rotterdam, the Netherlands
7 Department of Cardiology, Amsterdam University Medical Centers, VU University, Amsterdam, The Netherlands
8 Health Data Research UK and Institute of Health Informatics, University College London, London, United Kingdom
9 The Healthcare Innovation Center, Julius Center for Health Sciences and Primary Care, University Medical Center Utrecht, Utrecht University, Utrecht, the Netherlands
10 Department of Cardiology, Haga Teaching Hospital, Den Haag, the Netherlands
11 Department of Cardiology, Jeroen Bosch Hospital, 's-Hertogenbosch, the Netherlands
12 Department of Cardiology, Bernhoven Hospital, Uden, the Netherlands
13 Amsterdam Cardiovascular Sciences, Heart Failure and Arrhythmias, Amsterdam, The Netherlands
14 Department of Cardiology, St. Antonius Hospital, Nieuwegein/Utrecht, the Netherlands
15 Department of Cardiology, University Medical Center Utrecht, Utrecht, the Netherlands
16 Department of Cardiology, Diakonessenhuis, Utrecht, the Netherlands
17 Department of Cardiology, Deventer hospital, Deventer, the Netherlands
18 Department of Cardiology, Groene Hart Ziekenhuis Gouda, Gouda, the Netherlands
19 Department of Cardiology, Hospital Group Twente, Almelo & Hengelo, the Netherlands
20 Department of Cardiology, Zuyderland Medical Center, Heerlen & Sittard-Geleen, the Netherlands
21 Department of Cardiology, Dijklander hospital, Hoorn, The Netherlands
22 Department of Cardiology, Maasziekenhuis Pantein, Beugen, the Netherlands

***Corresponding author.** Department of General Practice & Nursing Science, Julius Center for Health Sciences and Primary Care, University Medical Center Utrecht, Utrecht University, Heidelberglaan 100, 3584 CX Utrecht, the Netherlands. Email: j.vaneijk-4@umcutrecht.nl

† **Contributed equally**

Word count:
4467

For peer review only - http://bmjopen.bmj.com/site/about/guidelines.xhtml

ABSTRACT

Introduction

Meta-analyses show positive effects of telemedicine in heart failure (HF) management on hospitalization, mortality, and costs. However, these effects are heterogeneous due to variation in the included HF population, telemedicine components, and the quality of the comparator usual care. Still, telemedicine is gaining acceptance in HF management. The current nationwide study aims to identify (i) in which subgroup(s) of HF patients telemedicine is (cost-)effective, and (ii) which components of telemedicine are most (cost-)effective.

Methods and analysis

The 'REsponsible roLl-out of E-heAlth through Systematic Evaluation – Heart Failure' study (RELEASE-HF) is a multi-center, observational, registry-based cohort study planning to enroll 6,480 HF patients using data from the HF registry facilitated by the Netherlands Heart Registration. Collected data include patient characteristics, treatment information, and clinical outcomes and are measured at HF diagnosis, 6 and 12 months afterwards. Components of telemedicine are described at hospital level based on close-ended interviews with clinicians and at patient level based on additional data extracted from Electronic Health Records and telemedicine generated data. Costs of telemedicine are calculated using registration data and interviews with clinicians and finance department staff. To overcome missing data additional national databases will be linked to the HF registry if feasible. Heterogeneity of effects of offering telemedicine compared to not offering on days alive without unplanned hospitalizations in 1 year are assessed across predefined patient characteristics using exploratory stratified analyses. Effects of telemedicine components are assessed by fitting separate models for component contrasts.

Ethics and dissemination

The study has been approved by the medical ethics committee 2021 at University Medical Center Utrecht (The Netherlands). Results will be published in peer-reviewed journals and presented at (inter)national conferences. Effective telemedicine scenarios will be proposed amongst hospitals, throughout the country and abroad, if applicable and feasible.

Trial registration number

NCT05654961

Key words

Study protocol
Heart failure
Telemedicine
Registry
Clinical effectiveness
Cost effectiveness

For peer review only

STRENGHTS AND LIMITATIONS OF THIS STUDY

- A strength of this study is using a nationwide registry-based cohort linked to national registries and databases to capture real world data on telemedicine use, heart failure patient characteristics, treatments, and clinically relevant outcomes with a 1-year follow-up.
- As another strength, data of the Heart failure registry in the Netherlands and a separate data collection on telemedicine features by additional questionnaires allow consideration of heterogeneous treatment effects across the heart failure population and telemedicine components.
- As a limitation, most data on telemedicine characteristics will be collected on a hospital level rather than on the individual patient level.
- As another limitation, cost-effectivity analyses are specific for the Dutch healthcare system, which may hamper generalizability of findings to other settings or countries.
- A finial limitation is that selection bias can occur when healthcare providers do not include all patients in the outpatient clinic in the Heart failure registry, because the Heart failure registry is not incorporated in care pathways in Electronic Health Record systems.”

For peer review only

INTRODUCTION

Heart failure (HF) poses a major socio-economic and patient burden. Healthcare systems are seeking innovative health models to support care. Optimized guideline-directed medical therapy (GDMT), self-care (i.e., healthy diet, medication adherence, exercise), and adequate monitoring of vital signs and symptoms may all help to reduce HF-related morbidity.[1-3]. Health models therefore use telemedicine as a tool to support patients in optimizing HF management, self-care support and symptom monitoring to improve care and prevent (re-)hospitalization.[2-4] "Telemedicine" is an heterogeneous intervention that includes a wide range of digital technologies (smartphones, mobile wireless devices/sensors, video connections, implantable devices, etc.) exchanging digital health information between patient and clinician to support and optimize the care process remotely.[5, 6]

Many meta-analyses have evaluated the (cost-)effectiveness of telemedicine for HF patients.[7-19] Overall, these meta-analyses point towards a positive effect of telemedicine on hospital admission, length of hospital stay, mortality, and costs.[2, 7-19] However, effects are heterogeneous across subgroups [20-24], and vary with telemedicine components, such as type of monitored functions, number of alerts and risk of alert fatigue, contact with health care professional, type of device, and different quality of usual care.[7, 14, 15, 19, 25] This can lead to problems in comparability of studies. Understanding of the cost-effectiveness of telemedicine is fragmented because studies do not evaluate the same costs, with the focus shifting between the purchase costs of telemedicine, personnel costs, and the variation in the costs of telemedicine components.[4, 12, 16]

As a result, HF guidelines lack specific recommendations on *how, when and in whom* telemedicine should be provided. Despite the lack of solid evidence and advice, payers (e.g., procurement officers, insurance companies) and patient organizations are advocating to accelerate implementation of telemedicine for HF patients in outpatient clinics.[26] Consequently, telemedicine is implemented in different formats with varying objectives, intervention components and implementation strategies. To reveal the real potential of telemedicine for HF patients and its implementation in everyday practice, clarity on *how, when and in whom,* and which telemedicine components are (cost-)effective, is essential.

Here, we present the protocol for the 'REsponsible roLl-out of E-heAlth through Systematic Evaluation – Heart Failure' study (RELEASE-HF). In this study, we focus on both patient-related uncertainties in telemedicine use and telemedicine intervention-related uncertainties, from a clinical as well as an economical perspective. Our specific study objectives are to examine: (1) which patient characteristics are related to an increase in number of days spent alive without unplanned hospitalizations within one year when telemedicine is part of HF care compared to regular HF care; (2) which components of telemedicine as part of HF care lead to an increased number of days alive without unplanned hospitalizations within one year; (3) which HF patient characteristics are related to cost-effectiveness when telemedicine is part of HF care compared to regular HF care; and (4) which components of telemedicine as part of HF care are cost-effective.

For peer review only - http://bmjopen.bmj.com/site/about/guidelines.xhtml

METHODS AND ANALYSIS

Study design

RELEASE-HF is a nationwide, observational, registry-based cohort study across multiple hospitals in the Netherlands.[27] The study collects routine data longitudinally. We record the health status of and cardiac interventions in HF patients, and simultaneously observe the natural (de)implementation of telemedicine. RELEASE-HF is linked to the Heart4Data consortium that started in 2022 to create a national and sustainable infrastructure for cardiovascular registry-based research in the Netherlands.[28] This infrastructure will develop a framework for the governance, ethical, legal, financial, information technology and methodological factors necessary for registry-based research.

Progress and time plan

RELEASE-HF started in June 2021 and will last four years (Figure 1). This study includes a collaboration with at least 29 hospitals that signed a letter of commitment. Additional hospitals may enter the study later if they can complete a 12-month follow-up per included patient through the HF registry.

Data sources

RELEASE-HF will combine various sources of data: the Netherlands Heart Registration (NHR) HF registry, other national databases, interviews, electronic health records (EHR), and telemedicine generated data (Figure 2). A detailed description of each data source is provided below.

<u>Heart failure registry</u>

The HF registry is an ongoing quality registry in the Netherlands, facilitated by the NHR; a non-profit organization that aims to contribute to quality improvement and safety in cardiac care.[29, 30] The HF registry serves as an ongoing learning healthcare system through benchmarking and quality control. Since 2019, it has been introduced as a voluntary, nationwide HF registration. Patient data is collected nonconsecutively and for the registry no informed consent is asked. Data of included patients are collected at three timepoints: at baseline, 6 months, and 12 months from time since diagnosis. The HF registry includes variables on three levels and are defined in a data dictionary: patient characteristics, treatment characteristics and clinically relevant outcomes. The data dictionary is a dynamic document (i.e., included variables and definitions may change over time) because it is based on current HF guidelines and compiled by a committee consisting of delegated cardiologists from Dutch hospitals. A detailed up to date data dictionary with an overview of the variables collected within the HF registry is always published at the NHR website (Supplementary material 1 provides an overview of the data dictionary of the HF registry, version September 2022. The content is subject to change).[31]

<u>National registries and databases</u>

If legally and technically feasible, we plan to link the HF registry data, specific for RELEASE-HF, to external data sources to (1) complement missing variables, (2) enrich and validate the HF registry, and (3) reduce registration burden for health care professionals/data managers. The external data sources considered are Statistics Netherlands (CBS), national health insurance database (VEKTIS),

For peer review only - http://bmjopen.bmj.com/site/about/guidelines.xhtml

national registry for hospital care (Dutch Hospital Data (DHD)) and national database for drug utilization and drug safety (PHARMO). Variables of interests are visits to the outpatient clinic, to the emergency room, and to the general practitioner related to HF, admission days at an intensive care unit, and treatment characteristics specific to HF (i.e., medication as class of diuretics).

Interviews on the use of telemedicine

Separate data collection will be performed using semi-structured interviews among clinicians and finance department staff on features and costs of telemedicine on hospital level (Supplementary material 2 provides the interview guide).

Electronic Health Records and telemedicine generated data

File search in EHR of patients and telemedicine generated data will be used to identify telemedicine components on patient level.

Study population

The HF registry comprises patients admitted to a Dutch hospital or outpatient clinic for HF irrespective of telemedicine use (figure 1). All patients who meet the diagnostic criteria for HF and its phenotype according to the ESC 2021 guidelines on HF are included.[1] If a patient has been diagnosed with HF in a setting other than the one where the patient currently presents (primary, secondary or tertiary care), the patient will also be included.[31] Current HF registry is not yet incorporated and implemented in care pathways in EHR systems, therefore selected patients fulfilling the NHR criteria are included in the HF registry and not on a consecutive basis or full coverage of all patients at the outpatient HF clinic. This may lead to selection bias. To prevent this selection bias, a researcher of RELEASE-HF is alerting health care professionals regularly to include all patients with HF in the HF registry, regardless of their telemedicine use, treatment and disease severity. Additionally, national working groups are progressing to fully incorporate the HF registry into the EHR.

Outcome measures

Primary outcome

Number of days alive without unplanned hospitalization within one year of follow-up is derived from the number of unplanned hospital days as collected in the HF registry (Table 1).

Table 1 Operationalization of exposure variable and study outcomes

Variable	Source	Time of collection	Operationalization
**Exposure**			
**Telemedicine**	HF registry, telemedicine generated data	T0, T1[a], T2	Whether a patient receives telemedicine. Five categories: 1. No telemonitoring[b], 2. Telemonitoring by telephone, 3. Telemonitoring, non-invasive based on traditional parameters (e.g., blood pressure, ECG), 4.

For peer review only - http://bmjopen.bmj.com/site/about/guidelines.xhtml

			Telemonitoring, using ICD based on HF parameters, and 5. Telemonitoring, invasive by sensors in the blood stream or heart.
**Primary outcome**			
**Number of days spent alive without unplanned hospitalizations within one year of follow-up**	HF registry	T1[a], T2	Number of days directly related to unplanned cardiac hospital admission due to HF. Number of admission days will be summed up over a period between follow-up moments and subtracted from 365 days.
**Secondary outcomes**			
**Costs**	Derived from HF registry, external data source, EHR, interviews	T0, T1[a], T2	Costs estimated from patient, disease, and treatment characteristics. Information taken into account includes medication use, whether the patient underwent cardiac interventions (e.g., pacemaker implantation, percutaneous coronary intervention), use of telemedicine, hospital admission days, visits to the outpatient clinic, visits to the emergency room, admission days at an intensive care unit, visits at the GP related to HF.
**All-cause mortality**	HF registry, external data source	T1[a], T2	Mortality status, determined after verification at the Personal Records Database (in Dutch: Basisregistratie Personen). Mortality is independent of HF (all-cause).
**Functional status**	HF registry	T0, T1[a], T2	New York Heart Association (NYHA) classification: a functional classification of patients, based on severity of symptoms and physical activity, with specific attention to fatigue, palpitation, and dyspnea. Scores are linked to one of four NYHA classes. Class I: no limitation, class II: slight limitation, class III: marked limitation, class IV: unable to carry on any physical activity without discomfort.[1]
**Health status**	HF registry	T0, T1[a], T2	SF-36 or SF-12 questionnaire (subset of SF-36)[32]: a validated patient-reported survey of patient health. Both questionnaires consist of eight sections with scores: vitality, physical functioning, bodily pain, general health perceptions, physical role functioning, emotional role functioning, social role functioning, and mental health. Each score is transformed into a 0-100 scale on the assumption that each question carries equal weight. The lower the score the more disability.
**Health related Quality of Life**	HF registry	T0, T1[a], T2	SF-36 or SF-12 questionnaire. Quality Adjusted Life Years (QALY) will be calculated based on the SF-6D: a model in which directly a single, preference-based score can be calculated for the SF-36 and SF-12.[33] Scores range from 0.0 (worst health state) to 1.0 (best health state).
**Healthcare utilization**	External data source, EHR	T1, T2	Healthcare utilization based on: 1) number of outpatient visits, plus 2) number of visits at the general practice related to HF.

[a] The HF registry collects data standard at baseline (T0), after 6 months (T1), after 12 months (T2). RELEASE-HF study is conform the timeframes of the HF registry. Therefore, data will be collected at 6 months, although the outcome measurements are after 12 months.
[b] The HF registry defines telemedicine as *telemonitoring*.
HF: Heart Failure; EHR: Electronic Health Record; ECG: Electrocardiogram; ICD: Implantable Cardioverter Defibrillator; GP: General Practitioner.

<u>Secondary outcomes</u>

For peer review only - http://bmjopen.bmj.com/site/about/guidelines.xhtml

Secondary outcomes are all-cause mortality, functional status, health status, hrQoL, healthcare utilization within one year of follow-up, and costs of care (Table 1).

Exposure measurement

Exposure to telemedicine is measured in the HF registry at 6 months and 12 months follow-up as non-use of telemedicine, telephone only, non-invasive telemedicine, implantable-cardioverter-defibrillator-based telemedicine, or invasive telemedicine (Table 1). Telemonitoring output can be different from the close-ended interviews that correspond to all outpatient clinic contacts in general. Components of the telemedicine intervention will be assessed on hospital level at one time during follow-up period. Table 2 provides an overview and operationalization of main components of the telemedicine intervention that have been described in existing literature or at local (e.g., hospital) level.[7, 34-36] The components will be validated and possibly further refined based on the interviews.

Table 2 Overview and operationalization telemedicine variables that are collected in the qualitative study

Component	Definition	Operationalization (examples)
**Supplier**	(External) Supplier of telemedicine for HF patients	Sanacoach, Luscii, Motiva/Philips, linked in personal environment in EHR
**Purpose of telemedicine**	The intention/motives for which the telemedicine intervention is administered: patient level and/or hospital level	Monitoring, prevent exacerbation, reduce workload, reduce costs, patient centered care
**Considering telemedicine**	First time a clinician considered telemedicine in HF management.	After diagnosis, (re)hospitalization, titration phase
**Structured telephone support**	Structured monitoring by telephone without using applications and devices specific to telemedicine and monitoring HF.	Present or not present
**Applications**	Technologies or platforms on which the patient could receive telemedicine	Smartphone, tablet, laptop, television, smartwatch
**Devices**	Accessory a patient could use to perform telemedicine	Blood pressure device (with or without Bluetooth), weight scale (with or without Bluetooth), smartwatch
**Involved health care workers**	Involved health care workers and their role in considering and executing telemedicine	HF nurse, nurse specialist, cardiologist
**Control centrum**	The presence or absence of a(n) (extern) control centrum to check the submitted measurements and questions	Present or not present
**Use of telemedicine**	Moment a patient could use telemedicine and have contact with clinicians	24/7, office hours, during weekend, at night
**Type of contact**	The manner of contact between the clinician and patient	Direct or indirect (store-and-forward) contact with a clinician
**Measurements**	Type of measurements: vital functions and HF related complaints, used to detect deterioration of and/or to monitor HF	Blood pressure, heart rate, weight, temperature, oxygen level, HF complaints as swelling ankles, nycturia, shortness of breath, tiredness, loss of appetite, coughing/wheezing, dizziness
**Notifications**	Messages from the telemedicine intervention. Notifications can be two-sided: from patient to clinician and vice versa	Automatic or non-automatic generated messages. Notifications present or not present.
**Modifiable aspects**	The presence or absence to set up *thresholds* of monitored vital function, and the possibility to tailor these thresholds per patient, severity, type of HF or	Available or not available

For peer review only - http://bmjopen.bmj.com/site/about/guidelines.xhtml

	other aspects of the telemedicine intervention (e.g., set up a tele-education environment).	
**Connection with EHR**	Feature of telemedicine if the intervention is integrated with the EHR. This means that the data entered by the patient through the telemedicine intervention is visible to the clinician in the EHR without using other applications.	Connected or not connected with EHR
**Education**	The presence or absence of an educational environment and the manner how this educational environment is shaped	Present or not present
**Educational topics**	The covered topics in the educational environment of telemedicine	Nutrition, behavior, exercise, medication
**Self-care**	The presence or absence of self-care modules and actions taken by the patient based on a digital advice or measurement	Available or not available
**Protocol**	Local (hospital) protocol which consists definitions on patient level about how often a patient should use telemedicine/monitor the vital signs and HF complaints, depending on the HF complaints or phase (i.e., titration, stable monitoring)	Low-intensity protocol defined as <2 times per week a measurement of vital signs. High-intensity protocol defined as ≥2 times per week a measurement of vital signs.
HF: Heart Failure; EHR: Electronic Health Record		

Additional variables

Additional information includes: age at HF diagnosis, sex, social economic status (SES), body mass index (BMI), New York Heart Association classification (NYHA), left ventricular ejection fraction (LVEF), etiology of HF, systolic and diastolic blood pressure, heart rhythm and rate. Comorbidities are chronic respiratory disorders, obstructive sleep apnea syndrome (OSAS), stroke, extra cardiac arterial vascular pathology, diabetes mellitus (DM), hypertension, anemia, chronic kidney disease (CKD), malignancy, heart rhythm disorders, depression, and thyroid disease. Overview of these variables can be found in table S1 and are published in detail at the NHR website.[31] Table 3 provides an overview and operationalization of variables derived from this HF registry.

Table 3 Operationalization of variables calculated from included variables in the HF registry

Variable	Operationalization
**Social economic status**	Score for socioeconomic status (including degree of education, wealth and employment history (WOA)) by zip code area according to 2019 data from the Social Cultural Planning Agency. Divided into quartiles or quintiles, where a 1 is a low SES-WOA score.
**Body mass index**	Derived from weight in kilogram and length in centimeters; calculated as weight / (length*length)
**Chronic kidney disease**	Based on cut-off values of eGFR calculated from serum creatinine level (collected by HF registry). The HF registry calculates eGFR as:<br>Male: (175*((creatinine level/88,4)^- 1,154)*(age Tn^-0,203)<br>Female: (175*((creatinine level/88.4)^- 1,154)*(age Tn^-0,203))*0,742<br><br>CKD = eGFR ≥60 mL/min/1.73 m^2<br>No CKD = eGFR <60 mL/min/1.73 m^2
**Depression**	Health Related QoL derived from the SF-12 or SF-36 questionnaire.

For peer review only - http://bmjopen.bmj.com/site/about/guidelines.xhtml

For peer review only

	Scoring:<br>0-100 = The lower the score the more disability. The higher the score the less disability i.e., a score of zero is equivalent to maximum disability and a score of 100 is equivalent to no disability.
SES: Social Economic Status; WOA: Wealth, educational level and employment history (in Dutch: welvaart, opleidingsniveau, arbeidsverleden); HF: Heart Failure; CKD: Chronic Kidney Disease; QoL: Quality of Life; SF-12 or SF-36: type of Short Form questionnaire	

Data collection and quality control

Data of the HF registry will be remotely monitored for major outliers and missing variables by the NHR, these processes have been described elsewhere.[30, 37] The NHR uses a unique individual patient identifier (NHRpersonID) to deduplicate registrations of the same patient (at the same or different hospitals) in the HF registry.[38] During the RELEASE-HF study, researchers will perform an additional check on completed data and provide assistance to hospitals to reduce missing values, as well as assistance to reduce the registration burden.

Interviews on telemedicine, using an interview guide with close-ended questions, will be performed by an investigator of RELEASE-HF and conducted with clinicians (e.g., cardiologist, HF nurse, nurse specialist, physician assistant). Interviews will be held during the 36-months period of data collection. All hospitals with telemedicine in HF care will be interviewed once, and the data will be checked with the hospitals once during period of data collection to be aware of changes in the components and status of telemedicine. Per hospital, the six-monthly costs of telemedicine are determined via interviews and registration data (if at hand). The units will be linked to the unit cost guideline provided by the Dutch National Health Care Institute (Dutch: Zorginstituut Nederland).[39] These interviews will be performed by a Health Technology Assessment (HTA) scientist, and conducted with clinicians or finance department staff. All participants provide informed consent.

Patient-level telemedicine data are collected by a clinician, data manager or researcher at the hospital where the patient is being treated, because they have access to the identification log of the HF registry. Collected data will include the start date of telemedicine use, and the frequency in which measurements are sent to the treating hospital, with a high rate typically referred to as a high-intensity (or acute) protocol and a low rate as a low-intensity (or stable) protocol.

The Heart4Data consortium will establish possibilities for data linkage between the HF registry and other real-world data sources, in collaboration with public partners (Figure 2).[28] At the moment of writing, a collaboration between other real-world data sources and the HF registry is not yet established for all external databases.

Statistical analysis

Clinical effectiveness

Descriptives are presented for all variables of the HF registry and telemedicine components. We will present the number of days without unplanned hospitalization for HF during 365 days after baseline, the average duration of unplanned HF-related hospital stays, the number of deaths, and time until death.

For peer review only - http://bmjopen.bmj.com/site/about/guidelines.xhtml

Preparation of the statistical model

As a first step, we describe how the overall effect of offering any type of telemedicine versus not offering telemedicine will be estimated. This analysis will subsequently be used for a comparison of telemedicine effects on days alive without unplanned HF-related hospitalization during one year across subgroups.

The cohort is restricted to individuals with newly diagnosed HF, that is, at most three months prior to baseline. The exposure contrast is defined as offering any type of telemedicine after first presentation with HF at the hospital outpatient clinic versus patients not offering telemedicine. The telemedicine status for the entire follow-up is defined by the 6-months after baseline measurement of telemedicine, used as an observational analog of an "intention to treat" analysis in which stopping of telemedicine is disregarded.

Because telemedicine administration is dependent on patient characteristics, confounding by indication will be accounted for. Based on clinical consensus, the following patient-level confounding variables measured at baseline will be adjusted for: age, sex, SES, BMI, NYHA, LVEF, etiology of HF, systolic blood pressure, diastolic blood pressure, heart rhythm, heart rate, chronic respiratory disorders, OSAS, stroke, extra cardiac arterial vascular pathology, DM, hypertension, anemia, CKD, malignancy, heart rhythm disorders, depression, and thyroid disease (Table 3 and S1).

The primary outcome model is a linear mixed model with a random intercept for participating center. It is specified as follows. The outcome is measured in days and because the final measurement may be less or more than 365 days after baseline, we rescale the number of days of unplanned hospitalization to the number of days under follow-up to 365 days. The outcome is computed by subtracting days of unplanned hospitalization or death from 365. The earlier described confounding variables are added to the primary outcome model to adjust for confounding. Continuous covariates are modelled using splines. Multicollinearity between confounding variables and the telemedicine status is evaluated. The coefficient for telemedicine in the primary outcome model represents the average treatment effect of offering any telemedicine within 3 months after HF diagnosis compared to not offering telemedicine, on days with unplanned HF-related hospitalization during a year in the population of newly diagnosed HF patients. Robust standard errors are estimated to compute confidence intervals

For the secondary outcome functional status, a similar approach is taken to estimate the average treatment effect of offering any telemedicine within 3 months after HF diagnosis compared to not offering telemedicine on functional status after one year in the population of newly diagnosed HF patients. For this analysis, the outcome model is a multinomial regression model and baseline functional status is added as a covariate. The risk difference relative to the reference outcome category is assessed using the predicted outcomes of the primary outcome model under offering of telemedicine versus not offering to obtain the marginal risk difference.[40-42] Similarly, for the secondary outcomes health status and hrQoL, the outcome model is a probit regression model and baseline measurements are added as a covariate. Healthcare utilization will be assessed using a Poisson regression model, and all-cause mortality will be assessed using binary logistic regression.

We plan to perform the following additional analyses. First, we plan to perform the analysis with the start of follow up defined at the T1 measurement and outcome of unplanned hospitalization days during 6 months. This is because telemedicine status can be misclassified when telemedicine is started shortly after baseline but is not registered in the data. Second, we plan to perform a per protocol analysis of telemedicine use if detailed information on starting and stopping of telemedicine

For peer review only - http://bmjopen.bmj.com/site/about/guidelines.xhtml

is available through linkage. Finally, we plan to perform all analyses in the entire cohort, rather than the cohort restricted on HF diagnosis at most 3 months prior to T0.

RQ 1: effectiveness of telemedicine in patient subgroups

Research question 1 investigates the effectiveness of telemedicine across subgroups of HF patients. Patient-related subgroups were identified by a systematic literature review of RCTs of telemedicine: age, severity of HF (NYHA class at baseline), sex, SES, presence of depression, atrial fibrillation (AF), type of HF (HFpEF, HFmrEF, HFrEF) (Table 3).[20-24, 43-51] Stratified analyses of the primary outcome model are performed to estimate the above defined average treatment effect in each of the subgroups. Heterogeneity in telemedicine effect across age is assessed on a continuous scale, where an interaction between age and telemedicine status is added to the primary outcome model and expected number of days out of hospital across age ranges 50 to 90 years are predicted from this model under telemedicine offered versus not offered.

In an additional analysis, heterogeneity across time since diagnosis is explored for the entire cohort without restricting to patients with a maximum of three months since HF diagnosis at baseline (diagnosed ≤3 months before baseline compared to >3 months before baseline).

The secondary outcomes functional status, health status, and hrQoL are assessed using similar approaches but instead using a multinomial, probit, and probit regression model, respectively. Healthcare utilization will be assessed using a Poisson regression model, and all-cause mortality will be assessed using binary logistic regression.

RQ 2: effectiveness of different telemedicine components

Research question 2 investigates the effectiveness of the different forms of telemedicine intervention. We assess this question by performing three separate analyses in which intervention aspects are contrasted in the subset of participants that received telemedicine. The presence or absence of a telemedicine component is determined on a hospital level. Components of telemedicine that are contrasted are: presence versus absence of a service center, presence versus absence of an educational module, high-intensity versus low-intensity protocol (Table 2).

The population of interest are newly diagnosed patients with HF (maximum 3 months prior to baseline) who received telemedicine. Offering of telemedicine is measured at 6-months after baseline.

In addition to the set of confounding variables at the patient level used in the analysis above, confounding variables at the hospital level are considered because indication for telemedicine is expected to differ across hospitals. Included confounding variables are: academic or non-academic hospital, presence of a specialized heart center, presence of an outpatient ward, size of outpatient ward (number of patients/year), staffing of the HF outpatient clinic, and full-time equivalent of outpatient-ward staff.

For each of the three contrasts, the primary outcome model is fitted to estimate the average treatment effect of a component relative to the corresponding reference component on days of unplanned hospitalisation during one year in the population of newly diagnosed HF patients that initiated telemedicine.

For peer review only - http://bmjopen.bmj.com/site/about/guidelines.xhtml

The secondary outcomes functional status, health status, and hrQoL are assessed using similar approaches but instead using a multinomial, probit, and probit regression model, respectively. Healthcare utilization and all-cause mortality will be assessed using a Poisson regression model, and binary logistic regression, respectively.

Cost effectiveness

Costs will be estimated by computing the average costs (using cost guideline provided by the National Health Care Institute) per individual based on the information collected by NHR and in the interviews with hospitals. Since 2023 Dutch hospitals can claim costs of telemedicine using Diagnosis Treatment Combination (DBCs) by the National Health Care Institute. These DBCs can be used to estimate the average costs.[39] A 95% credible interval (CE) will be computed using the percentiles of a Monte-Carlo bootstrap analysis with 5,000 resamplings. This average cost represents a sum of the costs of telemedicine use, inpatient days, days at ICU, HF related hospital procedures, and outpatient visits. In a secondary analysis, we will re-estimate average costs and 95% CE again using data enriched with VEKTIS data (if linking is available), meaning that costs of visits to the GP, pharmacy, and care at home are taken into account as well.

Linkage with CBS may be incomplete for several participants because of missing values or incomplete datasets (for example twins, different available time windows between registries of collected data). If the number of non-linked participants is below 10%, we will perform multiple imputation. If the number of non-linked participants is above 10%, we will perform HTA analysis on the complete subset.

Utility values will be estimated using the average hrQoL score collected in the HF registry and if needed from literature. The difference in average QoL score between the group that uses telemedicine versus the group that does not use telemedicine represents the difference in disutility between the groups under the assumption that the collected hrQoL is a good representation of the utility of the particular health state that individuals were in.

Quality Adjusted Life Years (QALYs) are estimated by multiplying the observed number of follow-up years by the corresponding hrQoL score. Subsequently, the Incremental Cost Effectiveness Ratio (ICER) is computed by taking the ratio between the difference of costs of the average patient in the telemedicine group and those not in the telemedicine group and the difference in QALY of both groups, thereby providing the cost per additional QALY gained. The 95% CEs are obtained using the Monte-Carlo percentile methods described above. Both deterministic and probabilistic sensitivity analyses will be performed to completely outline uncertainty on individual and combined parameters in the model.

In addition, willingness to pay curves will be drawn to draw impact of different threshold on cost-effectiveness outcomes.

RQ 3: cost-effectiveness of telemedicine in patient subgroups

For research question 3, costs, utility, QALYs, ICER, and willingness to pay curves will be computed to estimate the difference in cost-effectiveness between users of telemedicine and non-users of telemedicine in general. Subsequently, the analyses will be repeated to estimate cost-effectiveness in subgroups, similar to the groups defined for research question 2 in the clinical effectiveness analysis.

For peer review only - http://bmjopen.bmj.com/site/about/guidelines.xhtml

For peer review only

RQ 4: cost-effectiveness of different telemedicine components

For research question 4, costs, utility, QALYs, ICER, and willingness to pay curves will be computed to estimate the difference in cost-effectiveness between components of telemedicine, similar to the comparisons defined for research question 3 in the clinical effectiveness analysis.

Sample size calculation

The primary focus in RELEASE-HF is on the clinical effectiveness of telemedicine in HF management. Hence, the sample size calculation is based on parameters relevant to this analysis. The sample size calculation was conducted in three steps: (1) computing required sample size for the main effect of telemedicine on the primary outcome, (2) taking into account that subgroup effects are of main interest and (3) anticipating the accrual rate.

For the first part, we assumed a mean increase in number of days spent alive without unplanned hospitalization of 6.4 (SD 21.1) days based on findings of the TIM-HF2 study.[46] This corresponds to a required sample size of 432 patients in total, based on a type I error probability of 5%, a type II error probability of 20%, and accounting for 20% drop out.

Such a sample size would allow for an overall estimate of the difference between telemedicine and no telemedicine in days spent alive without unplanned hospitalization in the overall patient population, however our interest is in heterogeneous intervention effects. Hence, for the second part, the sample size was inflated to estimate subgroup effects.[52, 53] To be able to detect interaction effects that are 50% to 60% of the size of the overall effect (i.e., increase of more than 3.2 days to 3.8 days spent outside the hospital due to the interaction given the main effect) with a power of 80%, the required sample size is inflated with a factor 12.[52] This results in a required sample size of 5,184 individuals.

Finally, from the HF registry pilot study (CHECK-HF), we know that in view of the estimated average number of patients per hospital the average proportion of included outpatient clinic patients with HF was above 80%.[54] Taking this accrual rate into account we would require the inclusion of 6,480 HF patients.

Patient and public involvement

Patient and public organizations were involved during grant application. Public organizations were involved in recruiting hospitals, and legal support and advice. Health care professional involvement in the study includes participating in an interview about telemedicine and motives about telemedicine choice and use. Patients with HF and patient organizations will be involved in formulating and prioritizing relevant research questions which are in line with the need of the patient and the aim of RELEASE-HF. Results will be disseminated with involvement of patient and public organizations.

For peer review only - http://bmjopen.bmj.com/site/about/guidelines.xhtml

ETHICS AND DISSEMINATION

Management and storage of data

RELEASE-HF is an observational, retrospective multicentre cohort study of prospectively collected data registered within the NHR. A waiver for informed consent for analysis with the data of the NHR data registry was obtained. Data collection and registration is performed by the participating centres in a secured online environment. Detailed information on the process of data acquisition, completeness, data quality and analysis of the NHR has been published previously.[30] To obtain reliable data, the NHR has an advanced, certified data quality control system in place to ensure completeness and quality of data.[37]

Participants of the interview study will sign informed consent. Data will be stored in the secured environment at the UMC Utrecht, the Netherlands. Data will be pseudonymized on participant and setting level. A secured identification log will be used, only accessible by the main researcher of RELEASE-HF. Because the interview data will be linked to the HF registry, pseudonymized data will also be stored at the Medical Informatics Department of University Medical Centers Amsterdam (the Netherlands).

Storage of linked datasets (HF registry with other real-world data sources) will be part of the infrastructure established by the Heart4Data consortium.[28] RELEASE-HF study will follow these principles.

The Medical Ethics Committee of UMC Utrecht (the Netherlands) reviewed the study protocol and confirmed that the study does not fall under the scope of the Medical Research Involving Human Subjects Act (WMO). RELEASE-HF complies with the rules of the General Data Protection Regulation (GDPR).

Dissemination

The results will be published in peer-reviewed journals and presented at (inter)national conferences as deemed relevant for HF and telemedicine. The HF registry data underlying this article were provided by the NHR by the permission of the participating hospitals. Data are available upon reasonable request to the corresponding author and with permission of the NHR. The hospital specific telemedicine characteristics, which are added to the HF registry data will also only be available upon a reasonable request to the corresponding author and with permission of the participating hospitals.

DISCUSSION

The RELEASE-HF study is unique because of its observational study design, used to better understand heterogeneity in clinical effectiveness. To our knowledge, this is the first study using real-world data. The current design is chosen, because telemedicine is already implemented in various ways in health care settings in the Netherlands. Therefore, conducting a randomized controlled trial (RCT) does not fit the current care for HF patients. Additionally, using a national registry instead of a RCT allows us to observe all HF patients reducing selection bias that would otherwise be introduced by a RCT. A Dutch registry study previously showed that routinely collected data lead to a representative sample.[54] However, we have to be aware of confounding bias introduced by physicians or nurses who decide which patients may use telemedicine. We cannot completely avoid this bias, although we perform

For peer review only - http://bmjopen.bmj.com/site/about/guidelines.xhtml

For peer review only

extensive confounding correction and conduct interviews with these health care professionals and asking about their local guidelines to understand the selection of patients in that hospital. Another limitation of registry-based studies is that the data to be collected are predetermined, which can lead to relevant missing variables. To overcome the missing data, such as telemedicine data on patient level or outcome measures, we aim to link other data sources to enrich the RELEASE-HF dataset.

For peer review only

For peer review only - http://bmjopen.bmj.com/site/about/guidelines.xhtml

Acknowledgements

We would like to acknowledge all the study participants, cardiologists and nurses, from all participating hospitals for setting up the RELEASE-HF study and data collection in their hospital.

Study participants RELEASE-HF

John Younge, Marco van Gent (Albert Schweitzer Hospital); Jeroen Schaap (Amphia Hospital); M Louis Handoko (Amsterdam University Medical Centers); Ayten Erol-Yilmaz (Bernhoven hospital); Frederique Hafkamp (Netherlands Heart Network (NHN); Anna Hospital, Catharina hospital, Elkerliek, Máxima medical center); Wouter W Jansen Klomp (Deventer Hospital); Jaco Houtgraaf (Diakonessenhuis); Eric Wierda (Dijklander Hospital); Balázs Szabo (Elisabeth-TweeSteden Hospital); Rudolf A de Boer, Jasper J Brugts (Erasmus Medical Center); Stefan Koudstaal (Groene Hart Hospital); Jan Willem Borleffs (Haga Hospital); Gerard CM Linssen (Hospital Group Twente, Almelo & Hengelo); Dirk H van Dalen (Jeroen Bosch Hospital); Hans Peter Brunner-La Rocca (Maastricht University Medical Center); Jan van Ramshorst (Noord-West Hospital); Loek van Heerebeek (Onze Lieve Vrouwe Gasthuis); Stijn Wouters (Pantein Hospital); Roland van Kimmenade (Radboud University Medical Center); Gerardus PJ van Hout, Marco C Post (St. Antonius Hospital); Lennaert Kleijn (Treant Hospital); Manon G van der Meer (University Medical Center Utrecht); Sandra Sanders-van Wijk (Zuyderland Medical Center).

Contributors

JE contributed to protocol conception and design, development of statistical analyses, obtained ethics approval, writing the protocol manuscript, and critical revision of the protocol manuscript. KL contributed to protocol conception and design, development of statistical analyses, provided statistical and methodological support, writing the statistical analyses paragraph, and critical revision of the protocol manuscript. HR contributed to protocol conception and design, obtaining funding, development of statistical analyses, provided statistical and methodological support, and critically revised the manuscript. ES contributed to the development of statistical analyses, provided statistical and methodological support, and critically revised the manuscript. GF contributed to protocol conception and design, obtaining funding, writing the economic evaluation paragraph, and critically revised the manuscript. LD contributed to protocol conception for data collection combined with the use of the HF registry data, and critically revised the manuscript. JS and JJB are also local investigators and assisted with the data collection. TJ, JS, JJB, FHR, RAB contributed to protocol conception and design, obtaining funding, and critically revised the manuscript. FWA and JCT contributed to protocol conception and design, secured funding for the project, and critically revised the manuscript. Contributed equally to this paper. All authors approved the final version of the manuscript. The RELEASE-HF INVESTIGATORS consists of all local investigators who are responsible for ethical board approval and data collection. They have all read, refined and approved the final version of the manuscript.

For peer review only - http://bmjopen.bmj.com/site/about/guidelines.xhtml

Collaborators

On behalf of the RELEASE-HF INVESTIGATORS: C Jan Willem Borleffs, Dirk H van Dalen, Ayten Erol-Yilmaz, M Louis Handoko, Gerardus PJ van Hout, Jaco Houtgraaf, Wouter W Jansen Klomp, Stefan Koudstaal, Gerard CM Linssen, Manon G van der Meer, Marco C Post, Sandra Sanders-van Wijk, Eric Wierda, Stijn CW Wouters.

Funding

This study is supported by The Netherlands Organization for Health Research and Development (ZonMw grant 852002141). Additional support has been received from the Dutch CardioVascular Alliance (DCVA grant number is N/A). Hospitals receive 40 euros per included patient, paid by the RELEASE-HF study, with a complete one-year follow-up (i.e., data collected at three timepoints: at baseline, 6 months, and 12 months from time since diagnosis). This funding is only related to hospitals participated in the RELEASE-HF study, and will stop after finishing this study. The HF registry do not pay hospitals to participate in the registry.

Competing interests

Dr. De Boer has received research grants and/or fees from AstraZeneca, Abbott, Boehringer Ingelheim, Cardior Pharmaceuticals GmbH, Ionis Pharmaceuticals, Inc., Novo Nordisk, and Roche; and has had speaker engagements with Abbott, AstraZeneca, Bayer, Bristol Myers Squibb, Novartis, and Roche.

Dr. Borleffs has received speaker fees from AstraZeneca, Boehringer Ingelheim, Novartis.

Dr. Handoko is supported by the Dutch Heart Foundation (dr. E. Dekker Senior Clinical Scientist Grant 2020T058) and CVON (2020B008 RECONNEXT). MLH received an investigator-initiated research grant of Vifor Pharma, an educational grant of Boehringer Ingelheim and Novartis, and speaker/consultancy fees of Abbott, AstraZeneca, Bayer, Boehringer Ingelheim, MSD, Novartis, Sankyo, Dayichi, Quin, Vifor Pharma; all not related to this study.

Dr. Brugts received an independent research grant from Abbott for ISS paid to institution, and has had speaker engagements or advisory boards to Astra Zeneca, Abbott, Bayer, Boehringer, Danchii-Sankyo, Novartis and Vifor in the past 5 years.

For peer review only - http://bmjopen.bmj.com/site/about/guidelines.xhtml

REFERENCES

1. McDonagh TA, Metra M, Adamo M, et al. 2021 ESC Guidelines for the diagnosis and treatment of acute and chronic heart failure. *Eur Heart J* 2021;42(36):3599-726. doi: 10.1093/eurheartj/ehab368.
2. Anker SD, Koehler F, Abraham WT. Telemedicine and remote management of patients with heart failure. *Lancet* 2011;378(9792):731-9. doi: 10.1016/S0140-6736(11)61229-4.
3. Gensini GF, Alderighi C, Rasoini R, et al. Value of Telemonitoring and Telemedicine in Heart Failure Management. *Card Fail Rev* 2017;3(2):116-21. doi: 10.15420/cfr.2017:6:2.
4. Rosen D, McCall JD, Primack BA. Telehealth Protocol to Prevent Readmission Among High-Risk Patients With Congestive Heart Failure. *Am J Med* 2017;130(11):1326-30. doi: 10.1016/j.amjmed.2017.07.007.
5. Ayuso P. REPORT on the eHealth Action Plan 2012-2020 – Innovative healthcare for the 21st century, 2013.
6. Colucci M, Baldo V, Baldovin T, et al. A "matter of communication": A new classification to compare and evaluate telehealth and telemedicine interventions and understand their effectiveness as a communication process. *Health Informatics J* 2019;25(2):446-60. doi: 10.1177/1460458217747109.
7. Ding H, Chen SH, Edwards I, et al. Effects of Different Telemonitoring Strategies on Chronic Heart Failure Care: Systematic Review and Subgroup Meta-Analysis. *J Med Internet Res* 2020;22(11):e20032. doi: 10.2196/20032.
8. Hafkamp FJ, Tio RA, Otterspoor LC, et al. Optimal effectiveness of heart failure management - an umbrella review of meta-analyses examining the effectiveness of interventions to reduce (re)hospitalizations in heart failure. *Heart Fail Rev* 2022 doi: 10.1007/s10741-021-10212-8.
9. Inglis SC, Clark RA, Dierckx R, et al. Structured telephone support or non-invasive telemonitoring for patients with heart failure. *Cochrane Database Syst Rev* 2015(10):CD007228. doi: 10.1002/14651858.CD007228.pub3.
10. Kotb A, Cameron C, Hsieh S, et al. Comparative effectiveness of different forms of telemedicine for individuals with heart failure (HF): a systematic review and network meta-analysis. *PLoS One* 2015;10(2):e0118681. doi: 10.1371/journal.pone.0118681.
11. Kruse CS, Soma M, Pulluri D, et al. The effectiveness of telemedicine in the management of chronic heart disease - a systematic review. *JRSM Open* 2017;8(3):2054270416681747. doi: 10.1177/2054270416681747.
12. Lin MH, Yuan WL, Huang TC, et al. Clinical effectiveness of telemedicine for chronic heart failure: a systematic review and meta-analysis. *J Investig Med* 2017;65(5):899-911. doi: 10.1136/jim-2016-000199.
13. Pandor A, Thokala P, Gomersall T, et al. Home telemonitoring or structured telephone support programmes after recent discharge in patients with heart failure: systematic review and economic evaluation. *Health Technol Assess* 2013;17(32):1-207, v-vi. doi: 10.3310/hta17320.
14. Peters GM, Kooij L, Lenferink A, et al. The Effect of Telehealth on Hospital Services Use: Systematic Review and Meta-analysis. *J Med Internet Res* 2021;23(9):e25195. doi: 10.2196/25195.
15. Yun JE, Park JE, Park HY, et al. Comparative Effectiveness of Telemonitoring Versus Usual Care for Heart Failure: A Systematic Review and Meta-analysis. *J Card Fail* 2018;24(1):19-28. doi: 10.1016/j.cardfail.2017.09.006.
16. Driscoll A, Gao L, Watts JJ. Clinical effectiveness and cost-effectiveness of ambulatory heart failure nurse-led services: an integrated review. *BMC Cardiovasc Disord* 2022;22(1):64. doi: 10.1186/s12872-022-02509-9.
17. Pekmezaris R, Tortez L, Williams M, et al. Home telemonitoring in heart failure: a systematic review and meta-analysis. *Health Affairs* 2018;37(12):1983-89.

For peer review only - http://bmjopen.bmj.com/site/about/guidelines.xhtml

18. Brugts JJ, Radhoe SP, Clephas PRD, et al. Remote haemodynamic monitoring of pulmonary artery pressures in patients with chronic heart failure (MONITOR-HF): a randomised clinical trial. *Lancet* 2023;401(10394):2113-23. doi: 10.1016/S0140-6736(23)00923-6.
19. Scholte NT, Gürgöze MT, Aydin D, et al. Telemonitoring for heart failure: a meta-analysis. *European Heart Journal* 2023:ehad280.
20. Boyne JJ, Vrijhoef HJ, Crijns HJ, et al. Tailored telemonitoring in patients with heart failure: results of a multicentre randomized controlled trial. *Eur J Heart Fail* 2012;14(7):791-801. doi: 10.1093/eurjhf/hfs058.
21. Koehler F, Winkler S, Schieber M, et al. Telemedicine in heart failure: pre-specified and exploratory subgroup analyses from the TIM-HF trial. *Int J Cardiol* 2012;161(3):143-50. doi: 10.1016/j.ijcard.2011.09.007.
22. Koehler J, Stengel A, Hofmann T, et al. Telemonitoring in patients with chronic heart failure and moderate depressed symptoms: results of the Telemedical Interventional Monitoring in Heart Failure (TIM-HF) study. *Eur J Heart Fail* 2021;23(1):186-94. doi: 10.1002/ejhf.2025.
23. Galinier M, Roubille F, Berdague P, et al. Telemonitoring versus standard care in heart failure: a randomised multicentre trial. *Eur J Heart Fail* 2020;22(6):985-94. doi: 10.1002/ejhf.1906.
24. Krzesinski P, Jankowska EA, Siebert J, et al. Effects of an outpatient intervention comprising nurse-led non-invasive assessments, telemedicine support and remote cardiologists' decisions in patients with heart failure (AMULET study): a randomised controlled trial. *Eur J Heart Fail* 2022;24(3):565-77. doi: 10.1002/ejhf.2358.
25. Auener SL, Remers TEP, van Dulmen SA, et al. The Effect of Noninvasive Telemonitoring for Chronic Heart Failure on Health Care Utilization: Systematic Review. *J Med Internet Res* 2021;23(9):e26744. doi: 10.2196/26744.
26. Asselbergs FW, Baars F, Boyne JJJ, et al. Samenwerkingsafspraken en kwaliteitscriteria bij invoeren telebegeleiding bij hartfalen in Nederland 2016.
27. ClinicalTrials.gov. Identifier NCT05654961, REsponsible roLl-out of E-heAlth Through Systematic Evaluation - Heart Failure Study (RELEASE-HF) National Library of Medicine (US): Clinicaltrials.gov; 2022 [cited 2022 December 16]. Available from: https://classic.clinicaltrials.gov/ct2/show/NCT05654961?term=RELEASE-HF&draw=2&rank=1 accessed July 10 2023.
28. DCVA. Heart4Data [cited 2023 30 January]. Available from: https://dcvalliance.nl/heart4data accessed 30 January 2023.
29. NHR. Registratie [cited 2023 May 10]. Available from: https://nederlandsehartregistratie.nl/registratie/ accessed April 25 2022.
30. Timmermans MJ, Houterman S, Daeter ED, et al. Using real-world data to monitor and improve quality of care in coronary artery disease: results from the Netherlands Heart Registration. *Netherlands Heart Journal* 2022:1-9.
31. NHR. Handboek Nederlandse Hart Registratie | Hartfalen. 22.1.3 ed: Nederlandse Hart Registratie, 2022.
32. Aaronson NK, Muller M, Cohen PD, et al. Translation, validation, and norming of the Dutch language version of the SF-36 Health Survey in community and chronic disease populations. *J Clin Epidemiol* 1998;51(11):1055-68. doi: 10.1016/s0895-4356(98)00097-3.
33. Brazier JE, Roberts J. The estimation of a preference-based measure of health from the SF-12. *Med Care* 2004;42(9):851-9. doi: 10.1097/01.mlr.0000135827.18610.0d.
34. Bashshur R, Shannon G, Krupinski E, et al. The taxonomy of telemedicine. *Telemed J E Health* 2011;17(6):484-94. doi: 10.1089/tmj.2011.0103.
35. Edmunds M, Tuckson R, Lewis J, et al. An Emergent Research and Policy Framework for Telehealth. *EGEMS (Wash DC)* 2017;5(2):1303. doi: 10.13063/2327-9214.1303.
36. Tulu B, Chatterjee S, Maheshwari M. Telemedicine taxonomy: a classification tool. *Telemed J E Health* 2007;13(3):349-58. doi: 10.1089/tmj.2006.0055.

For peer review only - http://bmjopen.bmj.com/site/about/guidelines.xhtml

37. Houterman S, van Dullemen A, Versteegh M, et al. Data quality and auditing within the Netherlands Heart Registration: using the PCI registry as an example. *Neth Heart J* 2023 doi: 10.1007/s12471-022-01752-1.
38. NHR. Overzicht berekende velden - Nederlandse Hart Registratie [cited 2023 5 october]. Versie 23.1.0:[Available from: https://nhr.nl/wp-content/uploads/2022/11/NHR-Handboek-berekende-velden.pdf accessed 2 March 2023.
39. Nederland Z. Richtlijnen voor economische evaluatie [cited 2023 May 3]. Available from: https://www.zorginstituutnederland.nl/over-ons/werkwijzen-en-procedures/adviseren-over-en-verduidelijken-van-het-basispakket-aan-zorg/beoordeling-van-geneesmiddelen/richtlijnen-voor-economische-evaluatie2023.
40. Austin PC. Absolute risk reductions, relative risks, relative risk reductions, and numbers needed to treat can be obtained from a logistic regression model. *J Clin Epidemiol* 2010;63(1):2-6. doi: 10.1016/j.jclinepi.2008.11.004.
41. Greenland S. Model-based estimation of relative risks and other epidemiologic measures in studies of common outcomes and in case-control studies. *Am J Epidemiol* 2004;160(4):301-5. doi: 10.1093/aje/kwh221.
42. Localio AR, Margolis DJ, Berlin JA. Relative risks and confidence intervals were easily computed indirectly from multivariable logistic regression. *J Clin Epidemiol* 2007;60(9):874-82. doi: 10.1016/j.jclinepi.2006.12.001.
43. Chaudhry SI, Mattera JA, Curtis JP, et al. Telemonitoring in patients with heart failure. *N Engl J Med* 2010;363(24):2301-9. doi: 10.1056/NEJMoa1010029.
44. Cleland JG, Louis AA, Rigby AS, et al. Noninvasive home telemonitoring for patients with heart failure at high risk of recurrent admission and death: the Trans-European Network-Home-Care Management System (TEN-HMS) study. *J Am Coll Cardiol* 2005;45(10):1654-64. doi: 10.1016/j.jacc.2005.01.050.
45. Dendale P, De Keulenaer G, Troisfontaines P, et al. Effect of a telemonitoring-facilitated collaboration between general practitioner and heart failure clinic on mortality and rehospitalization rates in severe heart failure: the TEMA-HF 1 (TElemonitoring in the MAnagement of Heart Failure) study. *Eur J Heart Fail* 2012;14(3):333-40. doi: 10.1093/eurjhf/hfr144.
46. Koehler F, Koehler K, Deckwart O, et al. Efficacy of telemedical interventional management in patients with heart failure (TIM-HF2): a randomised, controlled, parallel-group, unmasked trial. *The Lancet* 2018;392(10152):1047-57.
47. Lynga P, Persson H, Hagg-Martinell A, et al. Weight monitoring in patients with severe heart failure (WISH). A randomized controlled trial. *Eur J Heart Fail* 2012;14(4):438-44. doi: 10.1093/eurjhf/hfs023.
48. Ong MK, Romano PS, Edgington S, et al. Effectiveness of Remote Patient Monitoring After Discharge of Hospitalized Patients With Heart Failure: The Better Effectiveness After Transition -- Heart Failure (BEAT-HF) Randomized Clinical Trial. *JAMA Intern Med* 2016;176(3):310-8. doi: 10.1001/jamainternmed.2015.7712.
49. Piotrowicz E, Pencina MJ, Opolski G, et al. Effects of a 9-Week Hybrid Comprehensive Telerehabilitation Program on Long-term Outcomes in Patients With Heart Failure: The Telerehabilitation in Heart Failure Patients (TELEREH-HF) Randomized Clinical Trial. *JAMA Cardiol* 2020;5(3):300-08. doi: 10.1001/jamacardio.2019.5006.
50. Szalewska D, Glowczynska R, Piotrowicz R, et al. An aetiology-based subanalysis of the Telerehabilitation in Heart Failure Patients (TELEREH-HF) trial. *ESC Heart Fail* 2021;8(2):1263-73. doi: 10.1002/ehf2.13189.
51. Pekmezaris R, Nouryan CN, Schwartz R, et al. A randomized controlled trial comparing telehealth self-management to standard outpatient management in underserved black and Hispanic patients living with heart failure. *Telemedicine and e-Health* 2019;25(10):917-25.

For peer review only - http://bmjopen.bmj.com/site/about/guidelines.xhtml

52. Brookes ST, Whitely E, Egger M, et al. Subgroup analyses in randomized trials: risks of subgroup-specific analyses; power and sample size for the interaction test. *J Clin Epidemiol* 2004;57(3):229-36. doi: 10.1016/j.jclinepi.2003.08.009.
53. Burke JF, Sussman JB, Kent DM, et al. Three simple rules to ensure reasonably credible subgroup analyses. *BMJ* 2015;351:h5651. doi: 10.1136/bmj.h5651.
54. Brugts JJ, Linssen GCM, Hoes AW, et al. Real-world heart failure management in 10,910 patients with chronic heart failure in the Netherlands : Design and rationale of the Chronic Heart failure ESC guideline-based Cardiology practice Quality project (CHECK-HF) registry. *Neth Heart J* 2018;26(5):272-79. doi: 10.1007/s12471-018-1103-7.

For peer review only

Figure legend

Figure 1: Overview time plan RELEASE-HF study.
HF: Heart Failure; RELEASE-HF: REsponsible roLl-out of E-heAlth through Systematic Evaluation – Heart Failure; NHR: Netherlands Heart Registration; EHR: Electronic Health Record.

Figure 2: Data sources and flow to set up the final RELEASE-HF dataset.
RELEASE-HF: REsponsible roLl-out of E-heAlth through Systematic Evaluation – Heart Failure; H4D: Heart for Data Consortium; HF: Heart Failure.

For peer review only

For peer review only - http://bmjopen.bmj.com/site/about/guidelines.xhtml

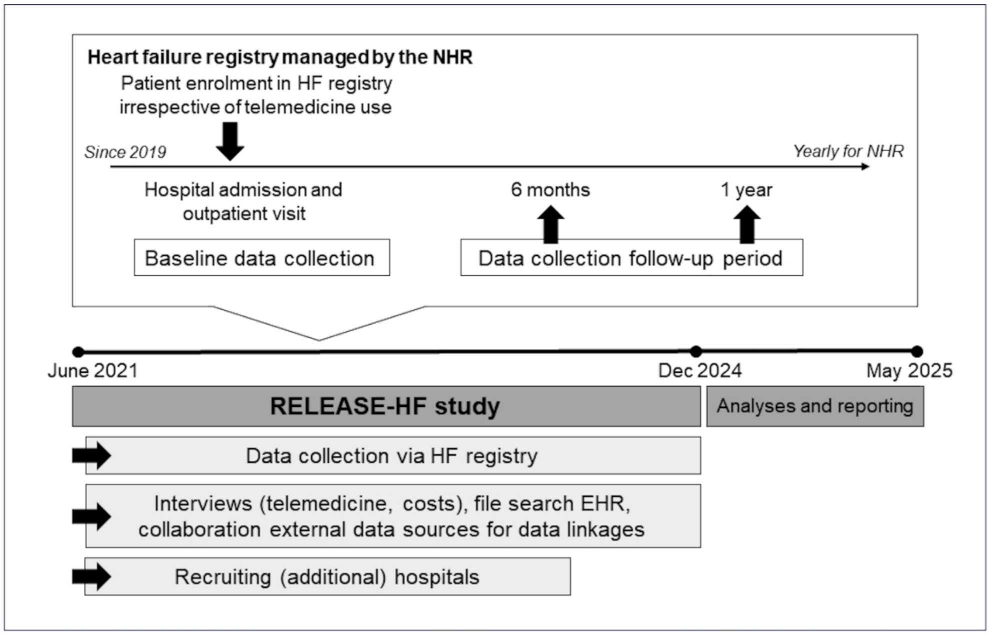

Figure 1 Overview time plan RELEASE-HF study. HF: Heart Failure; RELEASE-HF: REsponsible roLl-out of E-heAlth through Systematic Evaluation – Heart Failure; NHR: Netherlands Heart Registration; EHR: Electronic Health Record

256x161mm (300 x 300 DPI)

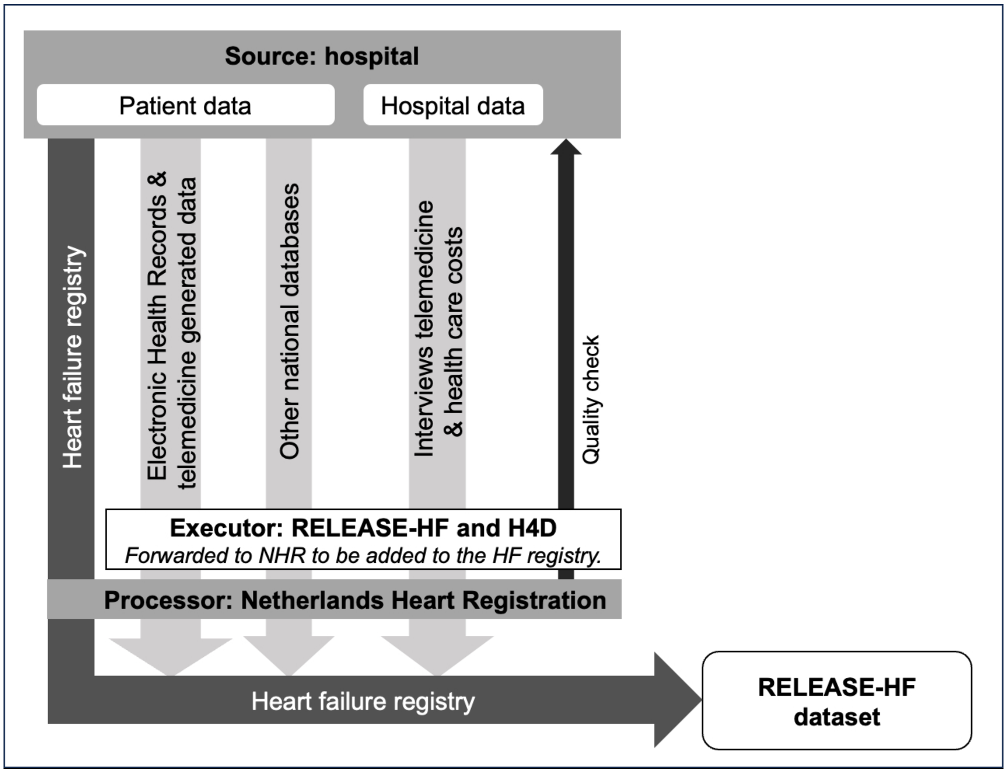

Figure 2 Data sources and flow to set up the final RELEASE-HF dataset. *RELEASE-HF: REsponsible roLl-out of E-heAlth through Systematic Evaluation – Heart Failure; H4D: Heart for Data Consortium; HF: Heart Failure.*

108x83mm (300 x 300 DPI)

Supplementary material 1 Variables of the heart failure registry

NOTE: Based on the dynamic data dictionary version 22.1.4 September 9, 2022. Definitions of variables and included variables are subject to change, since data dictionary depends on the current HF guidelines and is compiled by a committee consisting of delegated cardiologists from Dutch hospitals.

	Variables	Answer possibilities	Baseline (T0)	6 months (T1)	12 months (T2)
Identifying variables	Diagnosis number *	Unique number set up by the hospital	X		
	Date of diagnosis *	Date (DD-MM-YYYY)	X		
	Patient number*	Identification number in the hospital	X		
	Maiden name*	Name	X		
	Surname*	Name	X		
	Date of birth$	Date (DD-MM-YYYY)	X		
	Sex	Male<br>Female	X		
	Zip code*	1000 AA – 9999 ZZ= the Netherlands<br>0000= Abroad<br>-1= Unknown	X		
Diagnostic variables	Setting of inclusion	10= Outpatient clinic<br>20= Hospital, novo patient<br>30= Hospital, acute/chronic HF patient<br>-1 = Unknown	X		
	Previous HF diagnosis	0= No<br>1= Yes<br>-1= Unknown	X		
	Date of previous diagnosis (if applicable)	Date (DD-MM-YYYY)	X		
	Location diagnosis HF	10= Not earlier diagnosed with HF<br>10= Primary care<br>20= Secondary care<br>30= Tertiary care<br>-1= Unknown	X		
	Type of HF	10= HFrEF<br>20= HFmrEF<br>30= HFpEF<br>-1= Unknown	X		
	Etiology HFrEF	0= No HFrEF<br>10= Ischemic cardiomyopathy<br>20= Non-ischemic, hypertensive cardiomyopathy<br>50= Non-ischemic, arrhythmia/tachycardia mediated<br>60= Non-ischemic, hypertrophic cardiomyopathy<br>70= Non-ischemic, dilated cardiomyopathy (eci)<br>80= Non-ischemic, inflammation/infection (i.e., myocarditis)<br>90= Nonischemic, restrictive cardiomyopathy and/or accumulation<br>111= Nonischemic, familiar cq. genetic cardiomyopathy with proven DNA mutation	X		

For peer review only - http://bmjopen.bmj.com/site/about/guidelines.xhtml

		120= Non-ischemic, pericardial<br>135= Non-ischemic, toxic cardiomyopathy due to alcohol/drugs<br>136= Non-ischemic, toxic cardiomyopathy due to chemotherapy/radiation<br>139= Non-ischemic, toxic cardiomyopathy due to other<br>140= Non-ischemic, eci or (as yet) undetermined<br>900= Other (i.e., valve disease, metabolic, peripartum)<br>-1= Unknown			
	Etiology HFpEF	0= No HFpEF<br>10= Secondary HFpEF<br>20= Isolated hypertensive heart disease<br>30= Genetic HCM<br>40= Amyloidosis<br>50= Sarcoidosis<br>60= Hemochromatosis<br>70= Immune/inflammatory<br>80= Aortic valve stenosis<br>90= Pericardial diseases<br>900= Other<br>-1= Unknown	X		
**Patient characteristics**	***Identifying variables***				
	Follow-up moment	0 = T0<br>6 = T1<br>12 = T2<br>24 = T3<br>36 = T4<br>48 = T5	X	X	X
	Comorbidities				
	Hypertension	0= No<br>1= Yes<br>-1= Unknown	X	X	X
	Cardiovascular accident	0= No<br>1= Yes<br>-1= Unknown	X	X	X
	Extracardiac arterial vascular pathology	0= No<br>1= Yes<br>-1= Unknown	X	X	X
	Chronic respiratory disease	0= No<br>1= Yes<br>-1= Unknown	X	X	X
	Obstructive Sleep Apnea Syndrome	0= No<br>1= Yes<br>-1= Unknown	X	X	X
	Diabetes Mellitus	0= No<br>1= Diabetes, treatment unknown<br>2= Diabetes, no treatment<br>10= Diabetes, diet<br>20= Diabetes, oral medication<br>30= Diabetes, insulin<br>90= Diabetes, other<br>-1= Unknown	X	X	X
	Thyroid disorder	0= No<br>10= Yes, hypothyroidism<br>20= Yes, hyperthyroidism	X	X	X

For peer review only

For peer review only - http://bmjopen.bmj.com/site/about/guidelines.xhtml

		-1= Unknown			
	Malignancy	0= No<br>10= Yes, in the past<br>20= Yes, currently<br>-1= Unknown	X	X	X
	Measurements				
	Length	Measurement in centimeters	X	X	X
	Weight	Measurement in kilogram	X	X	X
	Systolic blood pressure	Measurement in mmHg	X	X	X
	Diastolic blood pressure	Measurement in mmHg	X	X	X
	Heart rhythm	10= Sinus rhythm<br>20= Atrium fibrillation<br>30= Atrial pacing<br>90= Other<br>-1= Unknown	X	X	X
	Heartrate	Measurement in beats per minute	X	X	X
	Atrial heart rhythm disorder	0= No<br>1= Yes<br>-1= Unknown	X	X	X
	Left bundle branch block	0= No<br>10= Yes, own heart's electrical conduction<br>20= Yes, pacing rhythm<br>-1= Unknown	X	X	X
	QRS complex duration	Measurement in milliseconds	X	X	X
	Left ventricle ejection fraction, NYHA classification and laboratory parameters				
	Left ventricle ejection fraction	Measurement in percentages	X	X	X
	NYHA class	1= Class I<br>2= Class II<br>3= Class III<br>4= Class IV<br>-1= Unknown	X	X	X
	NT-proBNP	1 t/m 8000= Value in pmol/l<br>0= No NT-proBNP measurement<br>-1= Unknown	X	X	X
	Date measurement NT-proBNP[$]	Date (DD-MM-YYYY)	X	X	X
	Serum creatinine	Value in µmol/l	X	X	X
	Iron deficiency	1= No<br>2= Yes, absolute ferritin < 100 µg/L<br>3= Yes, relative ferritin 100-300 µg/L with transferrin saturation < 20%<br>-1= Unknown	X	X	X
	Anemia	0= No<br>1= Yes<br>-1= Unknown	X	X	X
**Medication**	***Medication with target dose***				
	Type ACE inhibitor	0= No ACE inhibitor prescribed at Tn<br>10= Captopril (td in ESC 2021 = 150 mg)<br>20= Enalapril (td in ESC 2021= 20-40 mg)<br>3-= Lisinopril (td in ESC 2021 = 20-35 mg)<br>40= Ramipril (td in ESC 2021 = 10 mg)<br>50= Perindopril (td in ESC 2021 = 8 mg)	(X)	X	X

For peer review only - http://bmjopen.bmj.com/site/about/guidelines.xhtml

	60= Quinapril (td in ESC 2021 = 20 mg)<br>70= Fosinopril (td in ESC 2021 = 20-40 mg)<br>90= Other<br>-1= Unknown			
Target dose ACE inhibitor	0= No ACE inhibitor prescribed at Tn<br>1= No, target dose in ESC 2021 not reached due to contraindication or otherwise<br>2= No, target dose in ESC 2021 not reached due to titration phase<br>10= Yes, target dose in ESC 2021 reached or maximally tolerable for this patient<br>-1= Unknown	**(X)**	**X**	**X**
Type ARB	0= No ARB prescribed at Tn<br>10= Candesartan (td in ESC 2021 = 32 mg)<br>20= Losartan (td in ESC 2021 = 150 mg)<br>30= Valsartan (td in ESC 2021 = 320 mg)<br>40= Telmisartan (td in ESC 2021 = 80 mg)<br>50= Irbesartan (td in ESC 2021 = 300 mg)<br>90= Other<br>-1= Unknown	**(X)**	**X**	**X**
Target dose ARB	0= No ARB prescribed at Tn<br>1= No, target dose in ESC 2021 not reached due to contraindication or otherwise<br>2= No, target dose in ESC 2021 not reached due to titration phase<br>10= Yes, target dose in ESC 2021 reached or maximally tolerable for this patient<br>-1= Unknown	**(X)**	**X**	**X**
ARNI	0= No<br>1= Yes (td in ESC 2021 = 194-206 mg)<br>-1= Unknown	**(X)**	**X**	**X**
Target dose ARNI	0= No ARNI prescribed at Tn<br>1= No, target dose in ESC 2021 not reached due to contraindication or otherwise<br>2= No, target dose in ESC 2021 not reached due to titration phase<br>10= Yes, target dose in ESC 2021 reached or maximally tolerable for this patient<br>-1= Unknown	**(X)**	**X**	**X**
Type beta-blocker	0 = No beta blocker prescribed at Tn<br>10= Bisoprolol (td in ESC 2021 = 10 mg)<br>20= Metoprolol (td in ESC 2021 = 200 mg)<br>30= Carvedilol (td in ESC 2021 = 50 mg)	**(X)**	**X**	**X**

For peer review only - http://bmjopen.bmj.com/site/about/guidelines.xhtml

For peer review only

		40= Nebivolol (td in ESC 2021 = 10 mg)<br>50= Atenolol (td in ESC 2021 = 100 mg)<br>60= Pindolol (td in ESC 2021 = 20 mg)<br>70= Propranolol (td in ESC 2021 = 160 mg)<br>90= Other<br>-1= Unknown			
	Target dose beta-blocker	0= No beta blocker prescribed at Tn<br>1= No, target dose in ESC 2021 not reached due to contraindication or otherwise<br>2= No, target dose in ESC 2021 not reached due to titration phase<br>10= Yes, target dose in ESC 2021 reached or maximally tolerable for this patient<br>-1= Unknown	**(X)**	**X**	**X**
	Type MRA	0= No MRA prescribed at Tn<br>10= Eplerenone (td in ESC 2021= 50 mg)<br>20= Spironolactone (td in ESC 2021= 50 mg)<br>90= Other<br>-1= Unknown	**(X)**	**X**	**X**
	Target dose MRA	0= No MRA prescribed at Tn<br>1= No, target dose in ESC 2021 not reached due to contraindication or otherwise<br>2= No, target dose in ESC 2021 not reached due to titration phase<br>10= Yes, target dose in ESC 2021 reached or maximally tolerable for this patient<br>-1= Unknown	**(X)**	**X**	**X**
	Type SGLT2 inhibitor	0= No SGLT2 inhibitor prescribed at Tn<br>10= Canagliflozin (td in ESC 2021 = 10 mg)<br>20= Dapagliflozin (td in ESC 2021 = 10 mg)<br>30= Empagliflozin (td in ESC 2021 = 10 mg)<br>90= Other<br>-1= Unknown	**(X)**	**X**	**X**
	Target dose SGLT2 inhibitor	0= No SGLT2 inhibitor prescribed at Tn<br>1= No, target dose in ESC 2021 not reached due to contraindication or otherwise<br>2= No, target dose in ESC 2021 not reached due to titration phase<br>10= Yes, target dose in ESC 2021 reached or maximally tolerable for this patient<br>-1= Unknown	**(X)**	**X**	**X**
	Ivabradine	0= No<br>1= Yes	**(X)**	**X**	**X**

For peer review only - http://bmjopen.bmj.com/site/about/guidelines.xhtml

		-1= Unknown			
	Target dose Ivabradine	0= No ivabradine prescribed at Tn<br>1= No, target dose in ESC 2021 not reached due to contraindication or otherwise<br>2= No, target dose in ESC 2021 not reached due to titration phase<br>10= Yes, target dose in ESC 2021 reached or maximally tolerable for this patient<br>-1= Unknown	**(X)**	**X**	**X**
	Additional medication				
	Diuretic	0= No<br>1= Yes (target dose in ESC 2021 = 15mg)<br>-1= Unknown	**(X)**	**X**	**X**
	Vericiguat	0= No<br>1= Yes<br>-1= Unknown	**(X)**	**X**	**X**
	Digoxin	0= No<br>1= Yes<br>-1= Unknown	**(X)**	**X**	**X**
	Amiodarone	0= No<br>1= Yes<br>-1= Unknown	**(X)**	**X**	**X**
	Sotalol	0= No<br>1= Yes<br>-1= Unknown	**(X)**	**X**	**X**
	Hydralazine / Isosorbide dinitrate	0= No<br>1= Yes<br>-1= Unknown	**(X)**	**X**	**X**
**Outcome variables**	***Cardiac intervention and implantable***				
	Pacemaker – VVI	0= No<br>1= Yes<br>-1= Unknown	**(X)**	**X**	**X**
	Pacemaker – VVI date$	Date (DD-MM-YYYY)	**(X)**	**X**	**X**
	Pacemaker – DDD	0= No<br>1= Yes<br>-1= Unknown	**(X)**	**X**	**X**
	Pacemaker – DDD date$	Date (DD-MM-YYYY)	**(X)**	**X**	**X**
	CRT-P	0= No<br>1= Yes<br>-1= Unknown	**(X)**	**X**	**X**
	CRT-P date$	Date (DD-MM-YYYY)	**(X)**	**X**	**X**
	CRT-D	0= No<br>1= No, because of medical condition<br>2= No, because of patients' wish<br>10= Yes<br>-1= Unknown	**(X)**	**X**	**X**
	CRT-D date$	Date (DD-MM-YYYY)	**(X)**	**X**	**X**
	ICD – VVI	0= No<br>1= No, because of medical condition<br>2= No, because of patients' wish<br>10= Yes<br>-1= Unknown	**(X)**	**X**	**X**
	ICD – VVI date$	Date (DD-MM-YYYY)	**(X)**	**X**	**X**
	ICD – DDD	0= No<br>1= No, because of medical condition<br>2= No, because of patients' wish	**(X)**	**X**	**X**

For peer review only

For peer review only - http://bmjopen.bmj.com/site/about/guidelines.xhtml

		10= Yes<br>-1= Unknown			
	ICD – DDD date[$]	Date (DD-MM-YYYY)	**(X)**	**X**	**X**
	PCI	0= No<br>1= Yes<br>-1= Unknown	**(X)**	**X**	**X**
	PCI date[$]	Date (DD-MM-YYYY)	**(X)**	**X**	**X**
	CABG	0= No<br>1= Yes<br>-1= Unknown	**(X)**	**X**	**X**
	CABG date[$]	Date (DD-MM-YYYY)	**(X)**	**X**	**X**
	LVAD	0= No<br>1= Yes<br>-1= Unknown	**(X)**	**X**	**X**
	LVAD date[$]	Date (DD-MM-YYYY)	**(X)**	**X**	**X**
	Heart transplantation	0= No<br>1= Yes<br>-1= Unknown	**(X)**	**X**	**X**
	Heart transplantation date[$]	Date (DD-MM-YYYY)	**(X)**	**X**	**X**
	Heart revalidation	0= No<br>1= Yes<br>-1= Unknown	**(X)**	**X**	**X**
	Telemonitoring	0= No telemonitoring<br>10= Telemonitoring by telephone<br>20= Telemonitoring, non-invasive based on traditional parameters (e.g., blood pressure, ECG)<br>30= Telemonitoring, using ICD based on HF parameters<br>40= Telemonitoring, invasive by sensors in the blood stream or heart<br>-1= Unknown	**(X)**	**X**	**X**
	Patient status and quality of life				
	Mortality	0= Alive<br>1= Deceased<br>-1= Unknown		**X**	**X**
	Date mortality[$]	Date (DD-MM-YYYY)		**X**	**X**
	Number of hospitalizations	Number		**X**	**X**
	Date hospitalization[$]	Date (DD-MM-YYYY)		**X**	**X**
	Number of hospitalization days	Number		**X**	**X**
	Location follow-up	0= No follow-up, because of mortality<br>10= Patient stays in secondary care, outpatient HF clinic<br>20= Patient stays in tertiary care, outpatient HF clinic<br>30= Patient transferred to primary care<br>40= Patient transferred to the cardiology outpatient clinic<br>90= Other<br>-1= Unknown		**X**	**X**
	Type quality of life questionnaire	0= Not measured<br>10= SF12-2<br>20= SF36-2<br>30= SF36-1<br>-1= Unknown	**X**	**X**	**X**
	Quality of life	Coding depends on questionnaire	**X**	**X**	**X**

*Variables are collected in the Heart failure registry, but the data is not distributed and available for research.

For peer review only

For peer review only - http://bmjopen.bmj.com/site/about/guidelines.xhtml

$^{\$}$All variables which consist of a date will be transformed to number of days since diagnosed with HF. The actual date is not available in research.

HF: Heart Failure; HFrEF: Heart Failure with reduced Ejection Fraction; HFmrEF: Heart Failure with mildly reduced Ejection Fraction; HFpEF: Heart failure with preserved Ejection Fraction; NYHA: New York Heart Association; eci: e causa ignota (unknown cause); NT-proBNP: N-Terminal pro B-type Natriuretic Peptide; ACE: Angiotensin Converting Enzyme; td: target dose; ARB: Angiotensin Receptor Blocker; ARNI: Angiotensin Receptor-Neprilysin Inhibitor; MRA: Mineralocorticoid Receptor Antagonists; SGLT2: Sodium-glucose co-transporter-2; VVI: Ventricular pacing; DDD: dual-chamber antibradycardia pacing; CRT-P: Cardiac Resynchronization Therapy with a Pacemaker; CRT-D: Cardiac Resynchronization Therapy with a pacemaker and an ICD; ICD: Implantable Cardioverter-Defibrillator; PCI: Percutaneous Coronary Intervention; CABG: Coronary Artery Bypass Graft; LVAD: Left Ventricular Assist Device; ECG: Electro Cardio Gram; SF12/SF36: Short Form Health Survey with 12 questions or 36 questions.

For peer review only

Supplementary material 2 Interview guide telemedicine

Introduction

What do we mean by telemedicine?

With telemedicine, we mean the provision of care at a distance. The healthcare provider and the patient are not physically present with each other at that moment. Telemedicine can consist of telemonitoring or tele-education (according to the Collaboration Agreement and Quality Criteria for Telemedicine).

- *Telemonitoring* involves monitoring heart failure symptoms and relevant parameters used to support patients and healthcare providers in the treatment of heart failure.
- *Tele-education* is a service that allows the patient/user to receive remote education. Receiving this education can be accessed through a variety of applications and devices.

Background Information: Use of Telemedicine

1) Does your hospital offer telemedicine to outpatient heart failure patients?
   - Yes, we offer telemedicine.
     - i. Why do you offer telemedicine?
       - ➢ *Possibilities: monitoring, education, self-care/self-management, consultation, cost reduction, reduction in hospital admissions, improvement in quality of life, reducing workload.*
   - No, we do not offer telemedicine.
     - i. Why don't you offer telemedicine? (How strong is the influence of [x] in this?)
       - ➢ *Possibilities: no patient/clinician demand, costs, evidence, user-unfriendly technology, no connection to EHR, HF care pathway is different, time, shortage of healthcare professionals, significant investment compared to the amount of patients, legal/privacy concerns, others.*
     - ii. Have you considered using telemedicine for HF patients?
       - ➢ What prompted you to consider it but not proceed?
     - iii. Which professionals were involved in this decision?
   - No, but we plan to start telemedicine.
     - i. Why are you planning to start telemedicine?
       - ➢ *Possibilities: patient/clinician desire, guidelines, COVID-19, funding, others.*
     - ii. To what extent did COVID-19 influence this decision?
     - iii. Which professionals are involved in this decision?
   - No, we stopped telemedicine.
     - i. Why did you stop telemedicine?
       - ➢ *Possibilities: no patient demand, didn't meet expectations, more time, moderate positive effects, cost, lack of staff, others.*
     - ii. Who were involved in this decision? (Professionals)

Users of Telemedicine

From this point on, the conversation continues with the focus on the performers of telemedicine from the perspective of healthcare professional.

For peer review only - http://bmjopen.bmj.com/site/about/guidelines.xhtml

2) Which system do you use for telemedicine?
    - Luscii
    - Sananet/SanaCoach
    - Motiva (Philips)
    - 24Care
    - Hartwacht
    - CardioMEMS
    - Empower
    - Other, namely: ...

3) Why do you use telemedicine for heart failure patients?
    - Why is telemedicine used for [goal X]?
        i. *Functionalities: monitoring, education, self-care/self-management, consultation.*
        ii. *Endpoints: cost reduction, reduction in hospital admissions, improvement in quality of life, reducing workload for healthcare professionals.*
        iii. *Other, namely: ...*

For peer review only

Offering Telemedicine

4) Which patients visit the heart failure outpatient clinic?
    i. *Severity, age, duration of illness.*

5) Which heart failure patients at your outpatient clinic are eligible for telemedicine?
    i. What factors are considered in the decision to use telemedicine?
    ii. *Telemedicine guideline: Chronic HF, recently diagnosed, readmission due to exacerbation, anxious/uncertain/depressive patients, assistance in early detection of deterioration.*
    iii. *Characteristics: caregiver network, patient/donor digital skills, language proficiency, patient mobility, internet connection, distance to hospital, other.*
    - Does the severity of heart failure play a role in initiating telemedicine?
        - Yes
        - No
    - At which NYHA class is telemedicine used?
        - NYHA 1
        - NYHA 2
        - NYHA 3
        - NYHA 4
    - Why this NYHA class?

6) Which heart failure patients at your outpatient clinic are not eligible for telemedicine?
    - Why are these patients not eligible?
        i. *Telemedicine guideline: Chronic HF, recently diagnosed, readmission due to exacerbation, anxious/uncertain/depressive patients, assistance in early detection of deterioration.*
        ii. *Characteristics: caregiver network, patient/donor digital skills, language proficiency, patient mobility, internet connection, distance to hospital, other.*

7) When is telemedicine for the first time considered for heart failure patients? (multiple answers possible)
    - For every newly diagnosed heart failure patient
    - After a hospital admission

For peer review only - http://bmjopen.bmj.com/site/about/guidelines.xhtml

- For patients attending the heart failure outpatient clinic (regardless of previous hospital admission)
- After an exacerbation (patient is already diagnosed with heart failure)
    i. When is telemedicine offered after an exacerbation?
        - After 1 exacerbation
        - After ... exacerbations
        - If the patient has more than ... exacerbations
    i. Does the time period in which the patient has an exacerbation play a role in offering telemedicine?
        - Yes, when ... exacerbations in ... weeks
        - No
        - In the titration phase
    i. Is telemedicine offered during the titration phase?
        - Yes, every time there is a change in medication
        - Yes, only when the patient is newly diagnosed with the disease
        - Sometimes, depending on the situation, ease of titration, patient's distance, pandemic, etc.
        - No
    i. For which medication?
- In the stable phase of HF
    i. Is telemedicine offered in every stable phase of heart failure?
        - Yes
        - No
- Others, namely: ...

8) Which healthcare professionals are involved in determining whether or not to use telemedicine for the patient? (multiple answers possible)
    - Cardiologist
    - Heart failure nurse
    - Nurse Specialist / Physician Assistant
    - General Practitioner
    - Home care nurse
    - General practice nurse
    - Other, namely: ...

Stopping/On-Off principle of telemedicine

9) Is telemedicine offered multiple times in a heart failure care trajectory?
    - Yes
        i. How is this offered?
        ii. For which patients?
        iii. What determines the on/off/on/off principle?
        iv. Is the equipment removed?
    - No

10) When is telemedicine stopped?
    i. *Patient request, death, healthcare professional's assessment, disease severity, treatment phase, established time period, other*
    - Does NYHA class play a role in this?

For peer review only - http://bmjopen.bmj.com/site/about/guidelines.xhtml

Modules of the Telemedicine System

11) On which device can the patient receive telemedicine? *(multiple answers possible)*
    - Application on a mobile phone (smartphone)
    - Computer
    - Television
    - Watch (smartwatch)
    - iPad
    - Other, namely: ...

Telemonitoring

12) Does your telemedicine system include telemonitoring (e.g., vital signs, nutrition, medication, heart failure symptoms)?
    - Yes
        i. What is the main reason for using telemonitoring?
            - *Early detection of exacerbation, titration, longitudinal follow-up, self-management, other*
    - No

13) What type of telemonitoring do you use?
    - Non-invasive
    - Invasive
    - Non-invasive and invasive

14) Which measurements are monitored via telemonitoring?
    - Weight
    - Blood pressure
    - Heart rate
    - Heart rhythm (irregular, regular)
    - Temperature
    - Saturation
    - Intake (e.g., in the context of sodium restriction)
    - Fluid intake
    - Medication
    - Heart failure-related symptoms
        i. What symptoms do you monitor?
            - *Fatigue, decreased appetite, shortness of breath, swollen legs and ankles, cold hands and feet, a full feeling in the upper abdomen, palpitations, nocturia, constipation, tickling cough, restless sleep, memory and concentration problems, dizziness*
            - *Other, namely: ....*
    - Other, namely: ....

15) What equipment has the patient received for telemonitoring? *(multiple answers possible)*
    - No equipment
    - Patient uses their own equipment
        i. What equipment?
    - Weight scale
    - Bluetooth-enabled weight scale
    - Blood pressure monitor
    - Bluetooth-enabled blood pressure monitor
    - Pulse oximeter
    - Bluetooth-enabled pulse oximeter
    - ECG device

For peer review only - http://bmjopen.bmj.com/site/about/guidelines.xhtml

- CardioMEMS
- Other, namely: ....

16) Can a patient transmit monitored (vital) signs via the telemonitoring system to the healthcare provider?
    - Yes
        i. When can the patient transmit these monitored (vital) signs? (time of day)
            - *Possible options: 24/7, office hours, weekend, evening, night, set times, other, namely*
        ii. How does the patient transmit the monitored (vital) signs to the healthcare professional? (communication)
            - *Possible options: letter, phone call, video call, chat, SMS, email, via applications, other*
    - No

17) Does the healthcare professional receive a notification when monitored (vital) signs are entered into the telemonitoring system?
    - Yes
        i. When does the healthcare professional receive a notification?
            - *Possible options: always, when threshold values are exceeded, other*
        i. Where does the healthcare professional receive the notification?
            - *Possible options: Electronic Patient Record (EPD), standalone applications, email inbox, other*
        ii. Does the healthcare professional always receive the notification in the same manner, or does severity of exceeding the threshold play a role?
            - Yes
            - No
                i. What is the difference?
        iii. Which healthcare professional receives these messages?
            - *Possible options: cardiologist, heart failure nurse, nurse practitioner, physician assistant, general practitioner (GP), practice nurse, home care worker, other*
        iv. Are there erroneous notifications?
            - *How do you handle them?*
    - No
        v. How often does the healthcare professional evaluate the monitored vital signs, questionnaires, outcomes filled in by the patient in the telemonitoring system?
            a. Per week: ....
            b. Per day: ....
            c. Per patient: ....

18) Can a healthcare professional respond to the received monitored (vital) signs?
    - Yes
        i. Which healthcare professional responds to the received results?
            - *Possible options: cardiologist, heart failure nurse, nurse practitioner, physician assistant, general practitioner (GP), practice nurse, home care worker, other*
        ii. When can the healthcare professional respond to these monitored (vital) signs? (time of the day)
            - *Possible options: 24/7, office hours, weekend, evening, night, set times, other*
        iii. How does the healthcare professional respond to the monitored (vital) signs?
            - *Possible options: letter, phone call, video call, chat, SMS, email, via applications, other*
        iv. Does the patient receive a notification if the healthcare professional has sent a message to the patient?
    - No

For peer review only

19) Can thresholds be set for the monitored (vital) signs in the telemonitoring system?
- No
- Yes, general thresholds (not patient-specific)
  - i. What are the thresholds based on? / How are the thresholds formulated?
- Yes, patient-specific thresholds
  - i. Can the thresholds be adjusted per patient, per situation, per moment?
    - Yes
    - No
  - i. What are the thresholds based on? / How are the thresholds formulated?

20) Is there always contact between the patient and the healthcare professional for the monitored (vital) signs transmitted via the telemonitoring system?
- Yes
  - i. Is the contact direct or indirect (store-and-forward) between the patient and the healthcare professional?
    - ➢ *Direct: The patient and healthcare professional are using the system simultaneously, allowing for direct contact via one of the techniques provided by the telemonitoring system.*
    - ➢ *Indirect: The patient and healthcare professional have asynchronous contact with each other. The patient/healthcare professional sends their information to the recipient. The recipient reads the message later and responds to the sender.*
    - ➢ *Combination of direct and indirect contact*
- No
  - i. When is there contact between the patient and healthcare professional?

21) What actions does the healthcare professional take with the received monitored (vital) signs?
  - i. How do you initiate these actions?
    - ➢ *Possible options: letter, phone call, video call, chat, SMS, email, via applications, other*
  - ii. Does the telemonitoring system provide automatically generated advice to the patient when they enter data in a module in the telemonitoring system?
    - ➢ *Yes*
    - ➢ *No*

22) What is your experience with the telemonitoring provided by the telemonitoring system?

Education and self-care

23) Does your telemedicine system include the modules education and self-care?
- Yes
  - i. Does the education module need to be actively activated for each patient by the healthcare professional? (Note: Can the telemonitoring system also exist without the education module?)
    - ➢ Yes
    - ➢ No
  - ii. Which topics are included in the education?
    - ➢ General medical information about heart failure
    - ➢ Information about treatment
    - ➢ Prevention of symptoms/exacerbations
    - ➢ Medication use
    - ➢ Fluid and dietary management
    - ➢ Physical activity
    - ➢ Other, namely: ...

For peer review only - http://bmjopen.bmj.com/site/about/guidelines.xhtml

iii. Do you actively refer the patient to the education module in the telemedicine system? (Note: Do you discuss this topic in your consultation?)
   - Yes
   - No

i. Is the education module the same for every patient?

ii. Which self-care components are included in the telemedicine system?
   - Lifestyle interventions,
     - Which behaviors/lifestyle interventions are included?
       - *Possible answers: exercise, nutrition, stress management*
   - Psychological well-being: information, knowledge, tools
   - Patient's medication overview
   - Medication explanation (medication glossary)
   - Medication intake reminder as per prescription
   - Referral to external sources and links related to heart failure
   - Knowledge quiz/ability to test knowledge about heart failure
   - Monitoring
   - Self-detection and the ability to anticipate/adjust medication (e.g., detecting weight gain through monitoring and then taking and recording medication [diuretics])
   - Other, namely: ...

- No

24) To what extent does the patient make decisions based on the telemedicine system?
   - Are efforts made that the patient take independent action / anticipate in response to measured (vital) signs?

25) To what extent does the telemedicine system promote the patient's self-care? / What is the effect of this module on the patient's self-care (skills)?

26) What are your experiences with the built-in self-care and education modules in the telemedicine system?

Heart Failure Community

27) Can the patient use the telemedicine system to get in touch with peers (for example, a heart failure community)?
   - Yes, always
   - Yes, but this module needs to be activated
     i. How is the heart failure community organized?
        - *Local (hospital)*
        - *Regional*
        - *National*
   - No, this is not possible

For peer review only - http://bmjopen.bmj.com/site/about/guidelines.xhtml

Analog Telemonitoring (via telephone)

28) Do you have contact with heart failure patients by phone (hospital to patient)?
   - Yes
      - i. Who initiates contact with these patients by phone?
         - ➢ *Possible options: cardiologist, heart failure nurse, nurse practitioner (NP), physician assistant (PA), general practitioner (GP), general practice nurse (POH), home care nurse, other*
      - ii. When do you have contact with the patient by phone?
      - iii. What is the purpose of contact by phone?
      - iv. How often do you have contact with the patient by phone?
      - v. Which patients receive telephone guidance?
      - vi. Why these patients?
   - No

29) Can the patient initiate contact by phone with the heart failure outpatient clinic (patient to hospital)?
   - Yes
      - i. When can/may the patient initiate this type of contact?
      - ii. Who does the patient have telephone contact with?
         - ➢ *Possible options: cardiologist, heart failure nurse, nurse practitioner (NP), physician assistant (PA), general practitioner (GP), general practice nurse (POH), home care nurse, other*
   - No

30) Can a heart failure patient or healthcare professional seek contact in any other way?
   - Through which channel does this contact occur?
      - ➢ *Possible answer: patient portal*
   - When does this contact occur via this channel?

31) What is your experience with telemonitoring by phone?

External Influences on the Use of Telemonitoring
Evaluation

32) Is the telemedicine system evaluated (experiences, application in HF care, impact on objectives) among healthcare professionals?
   - Yes
   - No

33) What is the influence of telemedicine on the patient population that visits the (regular) heart failure outpatient clinic?
   - What are the characteristics of these patients?

34) Is the telemedicine system evaluated (experiences, application in their disease, usage) between the healthcare professional and the patient?
   - Yes
   - No
      - i. Are expectations discussed with the patient regarding the use of telemedicine?
      - ii. What feedback do you receive?
      - iii. What is the experience of telemedicine from the patients' perspective?

For peer review only - http://bmjopen.bmj.com/site/about/guidelines.xhtml

Experience

35) Do you believe that telemedicine in your hospital has led to efficiency?
   - Yes
     - i. On which outcomes?
     - ii. Has this been objectively assessed?
   - No
     - i. What makes you say that?

36) Would you recommend the telemedicine system you use?
   - Why?

37) What would you like to change/improve about your telemedicine system?

38) If you could choose a telemedicine system again, what would it look like?

COVID-19

39) Does COVID-19 have an impact on the use of telemedicine in your heart failure outpatient clinic? (multiple answers possible)
   - Yes, telemedicine is used for more patients as an intervention.
   - Yes, telemedicine is used less frequently for patients as an intervention.
   - Yes, telemedicine is used differently by patients who were already using this intervention before COVID-19.
     - i. Which devices of the telemedicine system are used differently since COVID-19?
       - ➢ *Possible answers: phone calls, video calls, chat, email, SMS, chatbot, other.*
     - i. Which modules of the telemedicine system are used differently since COVID-19?
       - ➢ *Possible answers: telemonitoring, education, self-care/self-management, heart failure community, other.*
     - i. What do you think of this development?
   - No, there is no difference in the use of telemedicine before, during, and in the current COVID-19 period.

Time Investment in telemedicine vs. No telemedicine in Heart Failure Care (HTA)

40) How much time do you, on average, spend on an appointment at a heart failure outpatient clinic without telemedicine?

    ... minutes / hours

41) How much time do you, on average, spend on an appointment at a heart failure outpatient clinic with telemedicine?

    ... minutes / hours

42) How much time does it take to explain telemedicine to a heart failure patient (for the initial setup)?

    ... minutes / hours

43) How much time do you, on average, spend per week on providing telemedicine? (this includes monitoring digital statuses transmitted through the telemonitoring system, contacting the patient as needed, evaluating measurements, etc.)

    ... minutes / hours

For peer review only - http://bmjopen.bmj.com/site/about/guidelines.xhtml

RELEASE-HF study protocol: An observational, registry-based study on the effectiveness of telemedicine in heart failure in the Netherlands

Journal:	*BMJ Open*
Manuscript ID	bmjopen-2023-078021.R2
Article Type:	Protocol
Date Submitted by the Author:	24-Nov-2023
Complete List of Authors:	van Eijk, Jorna; University Medical Centre Utrecht, General Practice & Nursing Science, Julius Center for Health Sciences and Primary Care<br>Luijken, Kim; University Medical Centre Utrecht, Epidemiology & Health Economics, Julius Center for Health Sciences and Primary Care<br>Jaarsma, Tiny; University Medical Centre Utrecht, General Practice & Nursing Science, Julius Center for Health Sciences and Primary Care<br>Reitsma, Johannes; University Medical Centre Utrecht, Epidemiology & Health Economics, Julius Center for Health Sciences and Primary Care<br>Schuit, Ewoud; University Medical Centre Utrecht, Epidemiology & Health Economics, Julius Center for Health Sciences and Primary Care<br>Frederix, Geert; Universitair Medisch Centrum Utrecht, Epidemiology & Health Economics, Julius Center for Health Sciences and Primary Care<br>Derks, Lineke; Netherlands Heart Registration<br>Schaap, Jeroen; Amphia Hospital, Cardiology; WCN, Dutch Network for Cardiovascular Research<br>Rutten, Frans; University Medical Centre Utrecht, General Practice & Nursing Science, Julius Center for Health Sciences and Primary Care<br>Brugts, Jasper; Erasmus Medical Center, Cardiology<br>de Boer, Rudolf; Erasmus Medical Center, Cardiology<br>Asselbergs, Folkert; Amsterdam University Medical Centres, Department of Cardiology; University College London, Health Data Research UK and Institute of Health Informatics<br>Trappenburg, Jaap; University Medical Centre Utrecht, The Healthcare Innovation Center, Julius Center for Health Sciences and Primary Care<br>investigators, RELEASE-HF ; University Medical Centre Utrecht
**Primary Subject Heading**:	Cardiovascular medicine
Secondary Subject Heading:	Patient-centred medicine, Health economics, Nursing
Keywords:	Heart failure < CARDIOLOGY, Telemedicine < BIOTECHNOLOGY & BIOINFORMATICS, REGISTRIES, HEALTH ECONOMICS, Protocols & guidelines < HEALTH SERVICES ADMINISTRATION & MANAGEMENT, Clinical Decision-Making

RELEASE-HF study protocol: An observational, registry-based study on the effectiveness of telemedicine in heart failure in the Netherlands

Authors:

Jorna van Eijk, MSc RN[1]*
Kim Luijken, PhD[2]
Tiny Jaarsma, PhD RN[1]
Johannes B Reitsma, PhD[2]
Ewoud Schuit, PhD[2]
Geert WJ Frederix, PhD[2]
Lineke Derks, MSc[3]
Jeroen Schaap, MD PhD[4, 5]
Frans H Rutten, MD PhD[1]
Jasper J Brugts, MD PhD MSc[6]
Rudolf A de Boer, MD PhD[6]
Folkert W Asselbergs, MD PhD[7, 8]†
Jaap CA Trappenburg, PhD[9] †
RELEASE-HF INVESTIGATORS

RELEASE-HF INVESTIGATORS (group authorship/collaborator):

C Jan Willem Borleffs, MD PhD[10]
Dirk H van Dalen, MSc RN[11]
Ayten Erol-Yilmaz, MD PhD[12]
M Louis Handoko, MD PhD MSc[7, 13]
Gerardus PJ van Hout, MD[14, 15]
Jaco Houtgraaf, MD PhD[16]
Wouter W Jansen Klomp, MD MSc[17]
Stefan Koudstaal, MD PHD MSc FESC FHFA[18]
Gerard CM Linssen, MD PhD FESC FHFA[19]
Manon G van der Meer, MD PhD[15]
Marco C Post, MD, PhD[14, 15]
Sandra Sanders-van Wijk, MD PhD[20]
Eric Wierda, MD PhD[21]
Stijn CW Wouters, MD PhD[22]

For peer review only - http://bmjopen.bmj.com/site/about/guidelines.xhtml

1 Department of General Practice & Nursing Science, Julius Center for Health Sciences and Primary Care, University Medical Center Utrecht, Utrecht University, Utrecht, the Netherlands
2 Department of Epidemiology & Health Economics, Julius Center for Health Sciences and Primary Care, University Medical Center Utrecht, Utrecht University, Utrecht, The Netherlands
3 Netherlands Heart Registration, Utrecht, The Netherlands
4 Department of Cardiology, Amphia Hospital, Breda, The Netherlands
5 Dutch Network for Cardiovascular Research (WCN), Utrecht, The Netherlands
6 Department of Cardiology, Erasmus Medical Center, Rotterdam, the Netherlands
7 Department of Cardiology, Amsterdam University Medical Centers, VU University, Amsterdam, The Netherlands
8 Health Data Research UK and Institute of Health Informatics, University College London, London, United Kingdom
9 The Healthcare Innovation Center, Julius Center for Health Sciences and Primary Care, University Medical Center Utrecht, Utrecht University, Utrecht, the Netherlands
10 Department of Cardiology, Haga Teaching Hospital, Den Haag, the Netherlands
11 Department of Cardiology, Jeroen Bosch Hospital, 's-Hertogenbosch, the Netherlands
12 Department of Cardiology, Bernhoven Hospital, Uden, the Netherlands
13 Amsterdam Cardiovascular Sciences, Heart Failure and Arrhythmias, Amsterdam, The Netherlands
14 Department of Cardiology, St. Antonius Hospital, Nieuwegein/Utrecht, the Netherlands
15 Department of Cardiology, University Medical Center Utrecht, Utrecht, the Netherlands
16 Department of Cardiology, Diakonessenhuis, Utrecht, the Netherlands
17 Department of Cardiology, Deventer hospital, Deventer, the Netherlands
18 Department of Cardiology, Groene Hart Ziekenhuis Gouda, Gouda, the Netherlands
19 Department of Cardiology, Hospital Group Twente, Almelo & Hengelo, the Netherlands
20 Department of Cardiology, Zuyderland Medical Center, Heerlen & Sittard-Geleen, the Netherlands
21 Department of Cardiology, Dijklander hospital, Hoorn, The Netherlands
22 Department of Cardiology, Maasziekenhuis Pantein, Beugen, the Netherlands

***Corresponding author.** Department of General Practice & Nursing Science, Julius Center for Health Sciences and Primary Care, University Medical Center Utrecht, Utrecht University, Heidelberglaan 100, 3584 CX Utrecht, the Netherlands. Email: j.vaneijk-4@umcutrecht.nl

† **Contributed equally**

Word count:
4496

For peer review only - http://bmjopen.bmj.com/site/about/guidelines.xhtml

ABSTRACT

Introduction

Meta-analyses show positive effects of telemedicine in heart failure (HF) management on hospitalization, mortality, and costs. However, these effects are heterogeneous due to variation in the included HF population, telemedicine components, and the quality of the comparator usual care. Still, telemedicine is gaining acceptance in HF management. The current nationwide study aims to identify (i) in which subgroup(s) of HF patients telemedicine is (cost-)effective, and (ii) which components of telemedicine are most (cost-)effective.

Methods and analysis

The 'REsponsible roLl-out of E-heAlth through Systematic Evaluation – Heart Failure' study (RELEASE-HF) is a multi-centre, observational, registry-based cohort study planning to enrol 6,480 HF patients using data from the HF registry facilitated by the Netherlands Heart Registration. Collected data include patient characteristics, treatment information, and clinical outcomes, and are measured at HF diagnosis, six and 12 months afterwards. Components of telemedicine are described at hospital level based on close-ended interviews with clinicians and at patient level based on additional data extracted from Electronic Health Records and telemedicine generated data. Costs of telemedicine are calculated using registration data and interviews with clinicians and finance department staff. To overcome missing data, additional national databases will be linked to the HF registry if feasible. Heterogeneity of effects of offering telemedicine compared to not offering on days alive without unplanned hospitalizations in one year are assessed across predefined patient characteristics using exploratory stratified analyses. Effects of telemedicine components are assessed by fitting separate models for component contrasts.

Ethics and dissemination

The study has been approved by the medical ethics committee 2021 at University Medical Center Utrecht (the Netherlands). Results will be published in peer-reviewed journals and presented at (inter)national conferences. Effective telemedicine scenarios will be proposed amongst hospitals throughout the country and abroad, if applicable and feasible.

Trial registration number

NCT05654961

Key words

Study protocol
Heart failure
Telemedicine
Registry
Clinical effectiveness
Cost effectiveness

For peer review only

STRENGTHS AND LIMITATIONS OF THIS STUDY

- A strength of this study is using nationwide routine clinical care data linked to national registry databases to capture current data on telemedicine use, heart failure patient characteristics, treatments, and clinically relevant outcomes with a one-year follow-up.
- Another strength is that data of the Heart Failure registry in the Netherlands and a separate data collection on telemedicine features using additional questionnaires, allow consideration of heterogeneous treatment effects across the heart failure population and telemedicine components.
- A limitation is that most data on telemedicine characteristics will be collected on a hospital level, rather than on the individual patient level.
- Another limitation is that cost-effectivity analyses are specific to the Dutch healthcare system, which may hamper generalizability of findings to other settings or countries.
- A final limitation is that selection bias can occur when healthcare providers do not include all patients in the outpatient clinic in the Heart Failure registry, because the registry is not incorporated in care pathways in Electronic Health Record systems.

For peer review only

INTRODUCTION

Heart failure (HF) poses a major socio-economic and patient burden. Healthcare systems are seeking innovative health models to support care. Optimized guideline-directed medical therapy (GDMT), self-care (i.e., healthy diet, medication adherence, exercise), and adequate monitoring of vital signs and symptoms may all help to reduce HF-related morbidity.[1-3] Health models therefore use telemedicine as a tool to support patients in optimizing HF management, self-care support and symptom monitoring to improve care and prevent (re-)hospitalization.[2-4] “Telemedicine” is a heterogeneous intervention that includes a wide range of digital technologies (smartphones, mobile wireless devices/sensors, video connections, implantable devices, etc.) exchanging digital health information between patient and clinician to support and optimize the care process remotely.[5, 6]

Many meta-analyses have evaluated the (cost-)effectiveness of telemedicine for HF patients.[7-19] Overall, these meta-analyses point towards a positive effect of telemedicine on hospital admission, length of hospital stay, mortality, and costs.[2, 7-19] However, effects are heterogeneous across subgroups, [20-24] and vary with telemedicine components, such as type of monitored functions, number of alerts and risk of alert fatigue, contact with healthcare professional, type of device, and different quality of usual care.[7, 14, 15, 19, 25] This can lead to problems in comparability of studies. Understanding of the cost-effectiveness of telemedicine is fragmented because studies do not evaluate the same costs, with the focus shifting between the purchase costs of telemedicine, personnel costs, and the variation in the costs of telemedicine components.[4, 12, 16]

As a result, HF guidelines lack specific recommendations on *how, when* and in *whom* telemedicine should be provided. Despite the lack of solid evidence and advice, payers (e.g., procurement officers, insurance companies) and patient organizations are advocating to accelerate implementation of telemedicine for HF patients in outpatient clinics.[26] Consequently, telemedicine is implemented in different formats with varying objectives, intervention components, and implementation strategies. To reveal the real potential of telemedicine for HF patients and its implementation in everyday practice, clarity on *how, when* and in *whom,* and which telemedicine components are (cost-)effective, is essential.

Here, we present the protocol for the ‘REsponsible roLl-out of E-heAlth through Systematic Evaluation – Heart Failure’ (RELEASE-HF) study. In this study, we focus on both patient-related uncertainties in telemedicine use and telemedicine intervention-related uncertainties, from a clinical as well as an economical perspective. Our specific study objectives are to examine: (1) which patient characteristics are related to an increase in number of days spent alive without unplanned hospitalizations within one year when telemedicine is part of HF care compared to regular HF care; (2) which components of telemedicine as part of HF care lead to an increased number of days alive without unplanned hospitalizations within one year; (3) which HF patient characteristics are related to cost-effectiveness when telemedicine is part of HF care compared to regular HF care; and (4) which components of telemedicine as part of HF care are cost-effective.

For peer review only - http://bmjopen.bmj.com/site/about/guidelines.xhtml

METHODS AND ANALYSIS

Study design

RELEASE-HF is a nationwide, observational, registry-based cohort study across multiple hospitals in the Netherlands.[27] The study collects routine data longitudinally. We record the health status of and cardiac interventions in HF patients, and simultaneously observe the natural (de)implementation of telemedicine. RELEASE-HF is linked to the Heart4Data consortium that started in 2022 to create a national and sustainable infrastructure for cardiovascular registry-based research in the Netherlands.[28] This infrastructure will develop a framework for the governance, ethical, legal, financial, information technology and methodological factors necessary for registry-based research.

Progress and time plan

RELEASE-HF started in June 2021 and will last four years (Figure 1). This study includes a collaboration with at least 29 hospitals that signed a letter of commitment. Additional hospitals may enter the study later if they can complete a 12-month follow-up per included patient through the HF registry.

Data sources

RELEASE-HF will combine various sources of data: the Netherlands Heart Registration (NHR) HF registry, other national databases, interviews, electronic health records (EHR), and telemedicine generated data (Figure 2). A detailed description of each data source is provided below.

Heart Failure registry

The HF registry is an ongoing quality registry in the Netherlands, facilitated by the NHR, a non-profit organization that aims to contribute to quality improvement and safety in cardiac care.[29, 30] The HF registry serves as an ongoing learning healthcare system through benchmarking and quality control. Since 2019, it has been introduced as a voluntary, nationwide HF registration. Patient data are collected non-consecutively, and for the registry no informed consent is asked. Data of included patients are collected at three timepoints: at baseline, six months, and 12 months from time since diagnosis. The HF registry includes variables on three levels defined in a data dictionary: patient characteristics, treatment characteristics, and clinically relevant outcomes. The data dictionary is a dynamic document (i.e., included variables and definitions may change over time) because it is based on current HF guidelines and compiled by a committee consisting of delegated cardiologists from Dutch hospitals. A detailed up-to-date data dictionary with an overview of the variables collected within the HF registry is always published on the NHR website (Supplementary material 1 provides an overview of the data dictionary of the HF registry, version September 2022. The content is subject to change).[31]

National registries and databases

If legally and technically feasible, we plan to link the HF registry data, specific for RELEASE-HF, to external data sources to (1) complement missing variables, (2) enrich and validate the HF registry, and (3) reduce registration burden for healthcare professionals/data managers. The external data sources considered are Statistics Netherlands (CBS), national health insurance database (VEKTIS),

For peer review only - http://bmjopen.bmj.com/site/about/guidelines.xhtml

national registry for hospital care (Dutch Hospital Data (DHD)), and national database for drug utilization and drug safety (PHARMO). Variables of interest are visits to the outpatient clinic, to the emergency room, and to the general practitioner related to HF, admission days at an intensive care unit, and treatment characteristics specific to HF (i.e., medication as class of diuretics).

Interviews on the use of telemedicine

Separate data collection will be performed using semi-structured interviews among clinicians and finance department staff on features and costs of telemedicine at the hospital level (Supplementary material 2 provides the interview guide).

Electronic Health Records and telemedicine generated data

File search in EHR of patients and telemedicine generated data will be used to identify telemedicine components at the patient level.

Study population

The HF registry comprises patients admitted to a Dutch hospital or outpatient clinic for HF irrespective of telemedicine use (figure 1). All patients who meet the diagnostic criteria for HF and its phenotype, according to the ESC 2021 guidelines on HF, are included.[1] If a patient has been diagnosed with HF in a setting other than the one where the patient currently presents (primary, secondary or tertiary care), the patient will also be included.[31] Current HF registry is not yet incorporated and implemented in care pathways in EHR systems, therefore selected patients fulfilling the NHR criteria are included in the HF registry and not on a consecutive basis or full coverage of all patients at the outpatient HF clinic. This may lead to selection bias. To prevent this selection bias, a researcher of RELEASE-HF is alerting healthcare professionals regularly to include all patients with HF in the HF registry, regardless of their telemedicine use, treatment, and disease severity. Additionally, national working groups are progressing to fully incorporate the HF registry into the EHR.

Outcome measures

Primary outcome

Number of days alive without unplanned hospitalization within one year of follow-up is derived from the number of unplanned hospital days as collected in the HF registry (Table 1).

Table 1 Operationalization of exposure variable and study outcomes

Variable	Source	Time of collection	Operationalization
**Exposure**			
**Telemedicine**	HF registry, telemedicine generated data	T0, T1[a], T2	Whether a patient receives telemedicine. Five categories: 1. No telemonitoring[b], 2. Telemonitoring by telephone, 3. Telemonitoring, non-invasive based on traditional parameters (e.g., blood pressure, ECG), 4. Telemonitoring

For peer review only - http://bmjopen.bmj.com/site/about/guidelines.xhtml

			using ICD based on HF parameters, and 5. Telemonitoring, invasive using sensors in the bloodstream or heart.
**Primary outcome**			
**Number of days spent alive without unplanned hospitalizations within one year of follow-up**	HF registry	T1[a], T2	Number of days directly related to unplanned cardiac hospital admission due to HF. Number of admission days will be summed up over a period between follow-up moments and subtracted from 365 days.
**Secondary outcomes**			
**Costs**	Derived from HF registry, external data source, EHR, interviews	T0, T1[a], T2	Costs estimated from patient, disease, and treatment characteristics. Information taken into account includes medication use, whether the patient underwent cardiac interventions (e.g., pacemaker implantation, percutaneous coronary intervention), use of telemedicine, hospital admission days, visits to the outpatient clinic, visits to the emergency room, admission days at an intensive care unit, visits at the GP related to HF.
**All-cause mortality**	HF registry, external data source	T1[a], T2	Mortality status, determined after verification at the Personal Records Database (in Dutch: Basisregistratie Personen). Mortality is independent of HF (all-cause).
**Functional status**	HF registry	T0, T1[a], T2	New York Heart Association (NYHA) classification: a functional classification of patients, based on severity of symptoms and physical activity, with specific attention to fatigue, palpitation, and dyspnoea. Scores are linked to one of four NYHA classes. Class I: no limitation, class II: slight limitation, class III: marked limitation, class IV: unable to carry on any physical activity without discomfort.[1]
**Health status**	HF registry	T0, T1[a], T2	SF-36 or SF-12 questionnaire (subset of SF-36)[32]: a validated patient-reported survey of patient health. Both questionnaires consist of eight sections with scores: vitality, physical functioning, bodily pain, general health perceptions, physical role functioning, emotional role functioning, social role functioning, and mental health. Each score is transformed into a 0-100 scale on the assumption that each question carries equal weight. The lower the score, the more disability.
**Health related Quality of Life**	HF registry	T0, T1[a], T2	SF-36 or SF-12 questionnaire. Quality Adjusted Life Years (QALY) will be calculated based on the SF-6D, a model in which a single, preference-based score can be directly calculated for the SF-36 and SF-12.[33] Scores range from 0.0 (worst health state) to 1.0 (best health state).
**Healthcare utilization**	External data source, EHR	T1, T2	Healthcare utilization based on 1) number of outpatient visits, plus 2) number of visits at the general practice related to HF.

[a] The HF registry collects data standard at baseline (T0), after six months (T1), and after 12 months (T2). The RELEASE-HF study conforms to the timeframes of the HF registry. Therefore, data will be collected at six months, although the outcome measurements are after 12 months.
[b] The HF registry defines telemedicine as *telemonitoring*.
HF: Heart Failure; EHR: Electronic Health Record; ECG: Electrocardiogram; ICD: Implantable Cardioverter Defibrillator; GP: General Practitioner.

<ins>Secondary outcomes</ins>

For peer review only - http://bmjopen.bmj.com/site/about/guidelines.xhtml

For peer review only

Secondary outcomes are all-cause mortality, functional status, health status, hrQoL, healthcare utilization within one year of follow-up, and costs of care (Table 1).

Exposure measurement

Exposure to telemedicine is measured in the HF registry at six months and 12 months follow-up as non-use of telemedicine, telephone only, non-invasive telemedicine, implantable-cardioverter-defibrillator-based telemedicine, or invasive telemedicine (Table 1). Telemonitoring output can be different from the close-ended interviews that correspond to all outpatient clinic contacts in general. Components of the telemedicine intervention will be assessed at the hospital level at one time during follow-up period. Table 2 provides an overview and operationalization of main components of the telemedicine intervention that have been described in existing literature or at local (e.g., hospital) level.[7, 34-36] The components will be validated and possibly further refined based on the interviews. Figure 3 illustrates the flow of data collected at the hospital level and at the patient level.

Table 2 Overview and operationalization of telemedicine variables collected in the qualitative study

Component	Definition	Operationalization (examples)
**Supplier**	(External) Supplier of telemedicine for HF patients	Sanacoach, Luscii, Motiva/Philips, linked in personal environment in EHR
**Purpose of telemedicine**	The intention/motives for which the telemedicine intervention is administered: patient level and/or hospital level	Monitoring, prevent exacerbation, reduce workload, reduce costs, patient centred care
**Considering telemedicine**	First time a clinician considered telemedicine in HF management	After diagnosis, (re)hospitalization, titration phase
**Structured telephone support**	Structured monitoring by telephone without using applications or devices specific to telemedicine and monitoring HF	Present or not present
**Applications**	Technologies or platforms on which the patient could receive telemedicine	Smartphone, tablet, laptop, television, smartwatch
**Devices**	Accessory a patient could use to perform telemedicine	Blood pressure device (with or without Bluetooth), weight scale (with or without Bluetooth), smartwatch
**Involved healthcare workers**	Involved healthcare workers and their role in considering and executing telemedicine	HF nurse, nurse specialist, cardiologist
**Control centrum**	The presence or absence of a control centrum to check the submitted measurements and questions	Present or not present
**Use of telemedicine**	When a patient could use telemedicine and have contact with clinicians	24/7, office hours, during weekend, at night
**Type of contact**	The manner of contact between the clinician and patient	Direct or indirect (store-and-forward) contact with a clinician
**Measurements**	Type of measurements: vital functions and HF-related complaints, used to detect deterioration of and/or to monitor HF	Blood pressure, heart rate, weight, temperature, oxygen level, HF complaints such as swelling ankles, nycturia, shortness of breath, tiredness, loss of appetite, coughing/wheezing, dizziness
**Notifications**	Messages from the telemedicine intervention. Notifications can be two-sided: from patient to clinician and vice versa	Automatic or non-automatic generated messages. Notifications present or not present
**Modifiable aspects**	The option to set up *thresholds* of monitored vital function, and the possibility to tailor these thresholds	Available or not available

For peer review only - http://bmjopen.bmj.com/site/about/guidelines.xhtml

	per patient, severity, type of HF or other aspects of the telemedicine intervention (e.g., set up a tele-education environment)	
**Connection with EHR**	Feature of telemedicine if the intervention is integrated with the EHR. This means that data entered by the patient through the telemedicine intervention is visible to the clinician in the EHR without using other applications	Connected or not connected with EHR
**Education**	The presence or absence of an educational environment and the manner in which this is shaped	Present or not present
**Educational topics**	The covered topics in the educational environment of telemedicine	Nutrition, behaviour, exercise, medication
**Self-care**	The presence or absence of self-care modules, and actions taken by the patient based on a digital advice or measurement	Available or not available
**Protocol**	Local (hospital) protocol which consists of definitions about how often a patient should use telemedicine/monitor the vital signs and HF complaints, depending on the HF complaints or phase (i.e., titration, stable monitoring)	Low-intensity protocol defined as measurement of vital signs <2 times per week. High-intensity protocol defined as measurement of vital signs ≥2 times per week
HF: Heart Failure; EHR: Electronic Health Record		

For peer review only

Additional variables

Additional information includes age at HF diagnosis, sex, social economic status (SES), body mass index (BMI), New York Heart Association classification (NYHA), left ventricular ejection fraction (LVEF), etiology of HF, systolic and diastolic blood pressure, heart rhythm and rate. Comorbidities are chronic respiratory disorders, obstructive sleep apnoea syndrome (OSAS), stroke, extra cardiac arterial vascular pathology, diabetes mellitus (DM), hypertension, anaemia, chronic kidney disease (CKD), malignancy, heart rhythm disorders, depression, and thyroid disease. Overview of these variables can be found in table S1 and are published in detail at the NHR website.[31] Table 3 provides an overview and operationalization of variables derived from this HF registry.

Table 3 Operationalization of variables calculated from included variables in the HF registry

Variable	Operationalization
**Social economic status**	Score for socioeconomic status (including degree of education, wealth and employment history (WOA)) by zip code area according to 2019 data from the Social Cultural Planning Agency. Divided into quartiles or quintiles, where 1 is a low SES-WOA score
**Body mass index**	Derived from weight in kilogram and length in centimetres; calculated as weight/(length*length)
**Chronic kidney disease**	Based on cut-off values of eGFR calculated from serum creatinine level (collected by HF registry). The HF registry calculates eGFR as<br>Male: (175*(creatinine level/88.4)^- 1.154)*(age Tn^-0,203)<br>Female: (175*(creatinine level/88.4)^- 1.154)*(age Tn^-0,203)*0,742<br><br>CKD = eGFR ≥60 mL/min/1.73 m^2<br>No CKD = eGFR <60 mL/min/1.73 m^2

For peer review only - http://bmjopen.bmj.com/site/about/guidelines.xhtml

**Depression**	Health Related QoL derived from the SF-12 or SF-36 questionnaire.<br><br>Scoring:<br>0-100 = The lower the score, the more disability. The higher the score the less disability, i.e., a score of zero is equivalent to maximum disability and a score of 100 is equivalent to no disability
SES: Social Economic Status; WOA: Wealth, educational level and employment history (in Dutch: welvaart, opleidingsniveau, arbeidsverleden); HF: Heart Failure; CKD: Chronic Kidney Disease; QoL: Quality of Life; SF-12 or SF-36: type of Short Form questionnaire	

Data collection and quality control

Data of the HF registry will be remotely monitored for major outliers and missing variables using the NHR; these processes have been described elsewhere.[30, 37] The NHR uses a unique individual patient identifier (NHRpersonID) to deduplicate registrations of the same patient (at the same or different hospitals) in the HF registry.[38] During the RELEASE-HF study, researchers will perform an additional check on completed data and provide assistance to hospitals to reduce missing values, as well as assistance to reduce the registration burden.

Telemedicine interviews, using an interview guide with close-ended questions, will be performed by a RELEASE-HF investigator and conducted with clinicians (e.g., cardiologist, HF nurse, nurse specialist, physician assistant). Interviews will be held during the 36-months period of data collection. All hospitals with telemedicine in HF care will be interviewed once, and the data will be checked with the hospitals once during the data collection period to be aware of changes in the components and status of telemedicine. For each hospital, the six-monthly costs of telemedicine are determined via interviews and registration data (if at hand). The units will be linked to the unit cost guideline provided by the Dutch National Health Care Institute (Dutch: Zorginstituut Nederland).[39] These interviews will be performed by a Health Technology Assessment (HTA) scientist, and conducted with clinicians or finance department staff. All participants will provide informed consent.

Patient-level telemedicine data are collected by a clinician, data manager or researcher at the hospital where the patient is being treated, because they have access to the identification log of the HF registry. Collected data will include the start date of telemedicine use, and the frequency at which measurements are sent to the treating hospital, with a high rate typically referred to as a high-intensity (or acute) protocol, and a low rate as a low-intensity (or stable) protocol.

The Heart4Data consortium will establish opportunities for data linkage between the HF registry and other national registry databases, in collaboration with public partners (Figure 2).[28] At the time of writing, a collaboration between these databases and the HF registry is not yet established for all external national databases.

Statistical analysis

<u>Clinical effectiveness</u>

Descriptives are presented for all variables of the HF registry and telemedicine components. We will present the number of days without unplanned hospitalization for HF during 365 days after baseline, the average duration of unplanned HF-related hospital stays, the number of deaths, and time until death.

For peer review only - http://bmjopen.bmj.com/site/about/guidelines.xhtml

Preparation of the statistical model

As a first step, we describe how the overall effect of offering any type of telemedicine versus not offering telemedicine will be estimated. This analysis will subsequently be used for a comparison of telemedicine effects on days alive without unplanned HF-related hospitalization during one year across subgroups.

The cohort is restricted to individuals with newly diagnosed HF, i.e., at most three months prior to baseline. The exposure contrast is defined as offering any type of telemedicine after first presentation with HF at the hospital outpatient clinic versus patients not offered telemedicine. The telemedicine status for the entire follow-up is defined by the six months after baseline measurement of telemedicine, used as an observational analogue of an "intention to treat" analysis in which the stopping of telemedicine is disregarded.

Because telemedicine administration is dependent on patient characteristics, confounding by indication will be accounted for. Based on clinical consensus, the following patient-level confounding variables measured at baseline will be adjusted for: age, sex, SES, BMI, NYHA, LVEF, etiology of HF, systolic blood pressure, diastolic blood pressure, heart rhythm, heart rate, chronic respiratory disorders, OSAS, stroke, extra cardiac arterial vascular pathology, DM, hypertension, anaemia, CKD, malignancy, heart rhythm disorders, depression, and thyroid disease (see Tables 3 and S1).

The primary outcome model is a linear mixed model with a random intercept for participating centre, and is specified as follows. The outcome is measured in days and because the final measurement may be less or more than 365 days after baseline, we rescale the number of days of unplanned hospitalization to the number of days under follow-up to 365 days. The outcome is computed by subtracting days of unplanned hospitalization or death from 365. The earlier described confounding variables are added to the primary outcome model to adjust for confounding. Continuous covariates are modelled using splines. Multicollinearity between confounding variables and the telemedicine status is evaluated. The coefficient for telemedicine in the primary outcome model represents the average treatment effect of offering any telemedicine within three months after HF diagnosis compared to not offering telemedicine, on days with unplanned HF-related hospitalization during a year in the population of newly diagnosed HF patients. Robust standard errors are estimated to compute confidence intervals

For the secondary outcome functional status, a similar approach is taken to estimate the average treatment effect of offering any telemedicine within three months after HF diagnosis compared to not offering telemedicine on functional status after one year in the population of newly diagnosed HF patients. For this analysis, the outcome model is a multinomial regression model, and baseline functional status is added as a covariate. The risk difference relative to the reference outcome category is assessed using the predicted outcomes of the primary outcome model offering of telemedicine versus not offering, to obtain the marginal risk difference.[40-42] Similarly, for the secondary outcomes health status and hrQoL, the outcome model is a probit regression model, with baseline measurements added as a covariate. Healthcare utilization will be assessed using a Poisson regression model, and all-cause mortality will be assessed using binary logistic regression.

We plan to perform the following additional analyses. First, we plan to perform the analysis with the start of follow-up defined at the T1 measurement and outcome of unplanned hospitalization days during six months. This is because telemedicine status can be misclassified when it is started shortly after baseline but is not registered in the data. Second, we plan to perform a per protocol analysis of

For peer review only - http://bmjopen.bmj.com/site/about/guidelines.xhtml

telemedicine use if detailed information on starting and stopping of telemedicine is available through linkage. Finally, we plan to perform all analyses in the entire cohort, rather than the cohort restricted on HF diagnosis to a maximum of three months prior to T0.

RQ 1: Effectiveness of telemedicine in patient subgroups

Research question 1 investigates the effectiveness of telemedicine across subgroups of HF patients. Patient-related subgroups were identified by a systematic literature review of RCTs of telemedicine: age, severity of HF (NYHA class at baseline), sex, SES, presence of depression, atrial fibrillation (AF), type of HF (HFpEF, HFmrEF, HFrEF) (Table 3).[20-24, 43-51] Stratified analyses of the primary outcome model are performed to estimate the above defined average treatment effect in each of the subgroups. Heterogeneity in telemedicine effect across age is assessed on a continuous scale, where an interaction between age and telemedicine status is added to the primary outcome model, and expected number of days out of hospital across age ranges 50 to 90 years are predicted from this model under telemedicine offered versus not offered.

In an additional analysis, heterogeneity across time since diagnosis is explored for the entire cohort, without restricting to patients with a maximum of three months since HF diagnosis at baseline (diagnosed ≤3 months before baseline compared to >3 months before baseline).

The secondary outcomes functional status, health status, and hrQoL are assessed using similar approaches but instead using a multinomial, probit, and probit regression model, respectively. Healthcare utilization will be assessed using a Poisson regression model, and all-cause mortality will be assessed using binary logistic regression.

RQ 2: Effectiveness of different telemedicine components

Research question 2 investigates the effectiveness of the different forms of telemedicine intervention. We assess this question by performing three separate analyses in which intervention aspects are contrasted in the subset of participants that received telemedicine. The presence or absence of a telemedicine component is determined on a hospital level. Components of telemedicine that are contrasted are: presence versus absence of a service centre, presence versus absence of an educational module, and high-intensity versus low-intensity protocol (Table 2).

The population of interest are newly diagnosed patients with HF (maximum three months prior to baseline) who received telemedicine. Offering of telemedicine is measured at six months after baseline.

In addition to the set of confounding variables at the patient level used in the analysis above, confounding variables at the hospital level are considered because indication for telemedicine is expected to differ across hospitals. Confounding variables include academic or non-academic hospital, presence of a specialized heart centre, presence of an outpatient ward, size of outpatient ward (number of patients/year), staffing of the HF outpatient clinic, and full-time equivalent of outpatient-ward staff.

For each of the three contrasts, the primary outcome model is fitted to estimate the average treatment effect of a component relative to the corresponding reference component on days of unplanned hospitalisation during one year in the population of newly diagnosed HF patients that initiated telemedicine.

For peer review only - http://bmjopen.bmj.com/site/about/guidelines.xhtml

The secondary outcomes functional status, health status, and hrQoL are assessed using similar approaches but instead using a multinomial, probit, and probit regression model, respectively. Healthcare utilization and all-cause mortality will be assessed using a Poisson regression model, and binary logistic regression, respectively.

Cost effectiveness

Costs will be estimated by computing the average costs (using cost guideline provided by the National Health Care Institute) per individual based on the information collected by NHR and in the interviews with hospitals. Since 2023, Dutch hospitals can claim costs of telemedicine using the National Health Care Institute's Diagnosis Treatment Combination (DBCs). These DBCs can be used to estimate the average costs.[39] A 95% credible interval (CE) will be computed using the percentiles of a Monte-Carlo bootstrap analysis with 5,000 resamplings. This average cost represents a sum of the costs of telemedicine use, inpatient days, days at ICU, HF-related hospital procedures, and outpatient visits. In a secondary analysis, we will re-estimate average costs and 95% CE using data enriched with VEKTIS data (if linking is available), meaning that costs of visits to the GP, pharmacy, and care at home are also taken into account.

Linkage with CBS may be incomplete for several participants because of missing values or incomplete datasets (for example twins, different available time windows between registries of collected data). If the number of non-linked participants is below 10%, we will perform multiple imputation; if it is above 10%, we will perform HTA analysis on the complete subset.

Utility values will be estimated using the average hrQoL score collected in the HF registry and, if needed, from literature. The difference in average QoL score between the group that uses telemedicine versus the group that does not represents the difference in disutility between the groups under the assumption that the collected hrQoL is a good representation of the utility of the particular health state that individuals were in.

Quality Adjusted Life Years (QALYs) are estimated by multiplying the observed number of follow-up years by the corresponding hrQoL score. Subsequently, the Incremental Cost Effectiveness Ratio (ICER) is computed by taking the ratio between the difference of costs of the average patient in the telemedicine group and those not in the telemedicine group, and the difference in QALY of both groups, thereby providing the cost per additional QALY gained. The 95% CEs are obtained using the Monte-Carlo percentile methods described above. Both deterministic and probabilistic sensitivity analyses will be performed to completely outline uncertainty on individual and combined parameters in the model.

In addition, willingness to pay curves will be drawn to highlight the impact of different threshold on cost-effectiveness outcomes.

RQ 3: Cost-effectiveness of telemedicine in patient subgroups

For research question 3, costs, utility, QALYs, ICER, and willingness to pay curves will be computed to estimate the difference in cost-effectiveness between users and non-users of telemedicine in general. Subsequently, the analyses will be repeated to estimate cost-effectiveness in subgroups, similar to the groups defined for research question 2 in the clinical effectiveness analysis.

For peer review only - http://bmjopen.bmj.com/site/about/guidelines.xhtml

RQ 4: Cost-effectiveness of different telemedicine components

For research question 4, costs, utility, QALYs, ICER, and willingness to pay curves will be computed to estimate the difference in cost-effectiveness between components of telemedicine, similar to the comparisons defined for research question 3 in the clinical effectiveness analysis.

Sample size calculation

The primary focus in RELEASE-HF is on the clinical effectiveness of telemedicine in HF management. Hence, the sample size calculation is based on parameters relevant to this analysis. The sample size calculation was conducted in three steps: (1) computing required sample size for the main effect of telemedicine on the primary outcome, (2) taking into account that subgroup effects are of primary interest, and (3) anticipating the accrual rate.

For the first part, we assumed a mean increase in number of days spent alive without unplanned hospitalization of 6.4 (SD 21.1) days based on findings of the TIM-HF2 study.[46] This corresponds to a required sample size of 432 patients in total, based on a type I error probability of 5%, a type II error probability of 20%, and accounting for 20% drop out.

Such a sample size would allow for an overall estimate of the difference between telemedicine and no telemedicine in days spent alive without unplanned hospitalization in the overall patient population; however, our interest is in heterogeneous intervention effects. Hence, for the second part, the sample size was inflated to estimate subgroup effects.[52, 53] To detect interaction effects that are 50% to 60% of the size of the overall effect (i.e., increase of more than 3.2 days to 3.8 days spent outside the hospital due to the interaction given the main effect) with a power of 80%, the required sample size is inflated by a factor 12.[52] This results in a required sample size of 5,184 individuals.

Finally, from the HF registry pilot study (CHECK-HF), we know that in view of the estimated average number of patients per hospital, the average proportion of included outpatient clinic patients with HF was above 80%.[54] Taking this accrual rate into account we would require the inclusion of 6,480 HF patients.

Patient and public involvement

Patient and public organizations were involved during grant application. Public organizations were involved in recruiting hospitals, and legal support and advice. Healthcare professionals' involvement in the study includes participating in an interview about telemedicine and motives about telemedicine choice and use. Patients with HF and patient organizations will be involved in formulating and prioritizing relevant research questions which are in line with the need of the patient and the aim of RELEASE-HF. Results will be disseminated with involvement of patient and public organizations.

For peer review only - http://bmjopen.bmj.com/site/about/guidelines.xhtml

ETHICS AND DISSEMINATION

Management and storage of data

RELEASE-HF is an observational, retrospective multicentre cohort study of prospectively collected data registered within the NHR. A waiver for informed consent for analysis with the data of the NHR data registry was obtained. Data collection and registration is performed by the participating centres in a secured online environment. Detailed information on the process of data acquisition, completeness, data quality and analysis of the NHR has been published previously.[30] To obtain reliable data, the NHR has an advanced, certified data quality control system in place to ensure completeness and quality of data.[37]

Participants of the interview study will sign informed consent. Data will be stored in the secured environment at the UMC Utrecht, the Netherlands. Data will be pseudonymized on participant and setting levels. A secured identification log will be used, only accessible by the main RELEASE-HF researcher. Because the interview data will be linked to the HF registry, pseudonymized data will also be stored at the Medical Informatics Department of University Medical Centers Amsterdam (the Netherlands).

Storage of linked datasets (HF registry with other national databases) will be part of the infrastructure established by the Heart4Data consortium.[28] The RELEASE-HF study will follow these principles.

The Medical Ethics Committee of UMC Utrecht (the Netherlands) reviewed the study protocol and confirmed that the study does not fall under the scope of the Medical Research Involving Human Subjects Act (WMO). RELEASE-HF complies with the rules of the General Data Protection Regulation (GDPR).

Dissemination

The results will be published in peer-reviewed journals and presented at (inter)national conferences as deemed relevant for HF and telemedicine. The HF registry data underlying this article were provided by the NHR with the permission of the participating hospitals. Data are available upon reasonable request to the corresponding author and with permission of the NHR. The hospital-specific telemedicine characteristics, which are added to the HF registry data, will also only be available upon reasonable request to the corresponding author and with permission of the participating hospitals.

DISCUSSION

The RELEASE-HF study is a large-scale observational study design, used to better understand heterogeneity in clinical effectiveness between patients using telemedicine. To our knowledge, this is the first study using routine clinical care data. The current design has been chosen because telemedicine is already implemented in various ways in healthcare settings in the Netherlands. Therefore, conducting a randomized controlled trial (RCT) does not fit the current care for HF patients. Additionally, using a national registry instead of a RCT allows us to observe all HF patients, reducing selection bias that would otherwise be introduced by a RCT. A Dutch registry study previously showed that routinely collected data lead to a representative sample.[54] However, we have to be aware of confounding bias introduced by physicians or nurses who decide which patients

For peer review only - http://bmjopen.bmj.com/site/about/guidelines.xhtml

may use telemedicine. We cannot completely avoid this bias, although we perform extensive confounding correction, and conduct interviews with these healthcare professionals, asking about their local guidelines so we understand the selection of patients in that hospital. Consequently, it has been argued that results from observational studies describe a patient outcome when using the intervention rather than assessing the response to the intervention.[55] Another limitation of registry-based studies is that the data to be collected are predetermined, which can lead to relevant missing variables. To overcome the missing data, such as telemedicine data on the patient level or outcome measures, we aim to link other data sources to enrich the RELEASE-HF dataset.

For peer review only

For peer review only - http://bmjopen.bmj.com/site/about/guidelines.xhtml

Acknowledgements

We would like to acknowledge all the study participants, cardiologists and nurses, from all participating hospitals for setting up the RELEASE-HF study and data collection in their hospital.

Study participants RELEASE-HF

John Younge, Marco van Gent (Albert Schweitzer Hospital); Jeroen Schaap (Amphia Hospital); M Louis Handoko (Amsterdam University Medical Centers); Ayten Erol-Yilmaz (Bernhoven Hospital); Frederique Hafkamp (Netherlands Heart Network (NHN); Anna Hospital, Catharina Hospital, Elkerliek, Máxima Medical Center); Wouter W Jansen Klomp (Deventer Hospital); Jaco Houtgraaf (Diakonessenhuis); Eric Wierda (Dijklander Hospital); Balázs Szabo (Elisabeth-TweeSteden Hospital); Rudolf A de Boer, Jasper J Brugts (Erasmus Medical Center); Stefan Koudstaal (Groene Hart Hospital); Jan Willem Borleffs (Haga Hospital); Gerard CM Linssen (Hospital Group Twente, Almelo & Hengelo); Dirk H van Dalen (Jeroen Bosch Hospital); Hans Peter Brunner-La Rocca (Maastricht University Medical Center); Jan van Ramshorst (Noord-West Hospital); Loek van Heerebeek (Onze Lieve Vrouwe Gasthuis); Stijn Wouters (Pantein Hospital); Roland van Kimmenade (Radboud University Medical Center); Gerardus PJ van Hout, Marco C Post (St. Antonius Hospital); Lennaert Kleijn (Treant Hospital); Manon G van der Meer (University Medical Center Utrecht); Sandra Sanders-van Wijk (Zuyderland Medical Center).

Contributors

JE contributed to protocol conception and design, development of statistical analyses, obtained ethics approval, writing, and critical revision of, the protocol manuscript. KL contributed to protocol conception and design, development of statistical analyses, provided statistical and methodological support, writing the statistical analyses paragraph, and critical revision of the protocol manuscript. HR contributed to protocol conception and design, obtaining funding, development of statistical analyses, provided statistical and methodological support, and critically revised the manuscript. ES contributed to the development of statistical analyses, provided statistical and methodological support, and critically revised the manuscript. GF contributed to protocol conception and design, obtaining funding, writing the economic evaluation paragraph, and critically revised the manuscript. LD contributed to protocol conception for data collection combined with the use of the HF registry data, and critically revised the manuscript. JS and JJB are also local investigators and assisted with the data collection. TJ, JS, JJB, FHR, and RAB contributed to protocol conception and design, obtaining funding, and critically revised the manuscript. FWA and JCT contributed to protocol conception and design, secured funding for the project, and critically revised the manuscript. FWA and JCT contributed equally to this paper. All authors approved the final version of the manuscript. The RELEASE-HF INVESTIGATORS consists of all local investigators who are responsible for ethical board approval and data collection. They have all read, refined and approved the final version of the manuscript.

For peer review only - http://bmjopen.bmj.com/site/about/guidelines.xhtml

Collaborators

On behalf of the RELEASE-HF INVESTIGATORS: C Jan Willem Borleffs, Dirk H van Dalen, Ayten Erol-Yilmaz, M Louis Handoko, Gerardus PJ van Hout, Jaco Houtgraaf, Wouter W Jansen Klomp, Stefan Koudstaal, Gerard CM Linssen, Manon G van der Meer, Marco C Post, Sandra Sanders-van Wijk, Eric Wierda, and Stijn CW Wouters.

Funding

This study is supported by the Netherlands Organization for Health Research and Development (ZonMw grant 852002141). Additional support has been received from the Dutch CardioVascular Alliance (DCVA grant number is N/A). Hospitals receive 40 euros per included patient, paid by the RELEASE-HF study, with a complete one-year follow-up (i.e., data collected at three timepoints: at baseline, six months, and 12 months from time since diagnosis). This funding is only related to hospitals participating in the RELEASE-HF study, and will stop when the study is complete. The HF registry does not pay hospitals to participate in the registry.

Competing interests

Dr De Boer has received research grants and/or fees from AstraZeneca, Abbott, Boehringer Ingelheim, Cardior Pharmaceuticals GmbH, Ionis Pharmaceuticals, Inc., Novo Nordisk, and Roche, and has had speaker engagements with Abbott, AstraZeneca, Bayer, Bristol Myers Squibb, Novartis, and Roche.

Dr Borleffs has received speaker fees from AstraZeneca, Boehringer Ingelheim, Novartis.

Dr Handoko is supported by the Dutch Heart Foundation (dr. E. Dekker Senior Clinical Scientist Grant 2020T058) and CVON (2020B008 RECONNEXT). He received an investigator-initiated research grant from Vifor Pharma, an educational grant from Boehringer Ingelheim and Novartis, and speaker/consultancy fees from Abbott, AstraZeneca, Bayer, Boehringer Ingelheim, MSD, Novartis, Sankyo, Dayichi, Quin, and Vifor Pharma, all of which were not related to this study.

Dr Brugts received an independent research grant from Abbott for ISS paid to institution, and has had speaker engagements or advisory boards for AstraZeneca, Abbott, Bayer, Boehringer, Danchii-Sankyo, Novartis and Vifor in the past five years.

For peer review only - http://bmjopen.bmj.com/site/about/guidelines.xhtml

REFERENCES

1. McDonagh TA, Metra M, Adamo M, et al. 2021 ESC Guidelines for the diagnosis and treatment of acute and chronic heart failure. *Eur Heart J* 2021;42(36):3599-726. doi: 10.1093/eurheartj/ehab368.
2. Anker SD, Koehler F, Abraham WT. Telemedicine and remote management of patients with heart failure. *Lancet* 2011;378(9792):731-9. doi: 10.1016/S0140-6736(11)61229-4.
3. Gensini GF, Alderighi C, Rasoini R, et al. Value of Telemonitoring and Telemedicine in Heart Failure Management. *Card Fail Rev* 2017;3(2):116-21. doi: 10.15420/cfr.2017:6:2.
4. Rosen D, McCall JD, Primack BA. Telehealth Protocol to Prevent Readmission Among High-Risk Patients With Congestive Heart Failure. *Am J Med* 2017;130(11):1326-30. doi: 10.1016/j.amjmed.2017.07.007.
5. Ayuso P. REPORT on the eHealth Action Plan 2012-2020 – Innovative healthcare for the 21st century, 2013.
6. Colucci M, Baldo V, Baldovin T, et al. A "matter of communication": A new classification to compare and evaluate telehealth and telemedicine interventions and understand their effectiveness as a communication process. *Health Informatics J* 2019;25(2):446-60. doi: 10.1177/1460458217747109.
7. Ding H, Chen SH, Edwards I, et al. Effects of Different Telemonitoring Strategies on Chronic Heart Failure Care: Systematic Review and Subgroup Meta-Analysis. *J Med Internet Res* 2020;22(11):e20032. doi: 10.2196/20032.
8. Hafkamp FJ, Tio RA, Otterspoor LC, et al. Optimal effectiveness of heart failure management - an umbrella review of meta-analyses examining the effectiveness of interventions to reduce (re)hospitalizations in heart failure. *Heart Fail Rev* 2022 doi: 10.1007/s10741-021-10212-8.
9. Inglis SC, Clark RA, Dierckx R, et al. Structured telephone support or non-invasive telemonitoring for patients with heart failure. *Cochrane Database Syst Rev* 2015(10):CD007228. doi: 10.1002/14651858.CD007228.pub3.
10. Kotb A, Cameron C, Hsieh S, et al. Comparative effectiveness of different forms of telemedicine for individuals with heart failure (HF): a systematic review and network meta-analysis. *PLoS One* 2015;10(2):e0118681. doi: 10.1371/journal.pone.0118681.
11. Kruse CS, Soma M, Pulluri D, et al. The effectiveness of telemedicine in the management of chronic heart disease - a systematic review. *JRSM Open* 2017;8(3):2054270416681747. doi: 10.1177/2054270416681747.
12. Lin MH, Yuan WL, Huang TC, et al. Clinical effectiveness of telemedicine for chronic heart failure: a systematic review and meta-analysis. *J Investig Med* 2017;65(5):899-911. doi: 10.1136/jim-2016-000199.
13. Pandor A, Thokala P, Gomersall T, et al. Home telemonitoring or structured telephone support programmes after recent discharge in patients with heart failure: systematic review and economic evaluation. *Health Technol Assess* 2013;17(32):1-207, v-vi. doi: 10.3310/hta17320.
14. Peters GM, Kooij L, Lenferink A, et al. The Effect of Telehealth on Hospital Services Use: Systematic Review and Meta-analysis. *J Med Internet Res* 2021;23(9):e25195. doi: 10.2196/25195.
15. Yun JE, Park JE, Park HY, et al. Comparative Effectiveness of Telemonitoring Versus Usual Care for Heart Failure: A Systematic Review and Meta-analysis. *J Card Fail* 2018;24(1):19-28. doi: 10.1016/j.cardfail.2017.09.006.
16. Driscoll A, Gao L, Watts JJ. Clinical effectiveness and cost-effectiveness of ambulatory heart failure nurse-led services: an integrated review. *BMC Cardiovasc Disord* 2022;22(1):64. doi: 10.1186/s12872-022-02509-9.
17. Pekmezaris R, Tortez L, Williams M, et al. Home telemonitoring in heart failure: a systematic review and meta-analysis. *Health Affairs* 2018;37(12):1983-89.

For peer review only - http://bmjopen.bmj.com/site/about/guidelines.xhtml

18. Brugts JJ, Radhoe SP, Clephas PRD, et al. Remote haemodynamic monitoring of pulmonary artery pressures in patients with chronic heart failure (MONITOR-HF): a randomised clinical trial. *Lancet* 2023;401(10394):2113-23. doi: 10.1016/S0140-6736(23)00923-6.
19. Scholte NT, Gürgöze MT, Aydin D, et al. Telemonitoring for heart failure: a meta-analysis. *European Heart Journal* 2023:ehad280.
20. Boyne JJ, Vrijhoef HJ, Crijns HJ, et al. Tailored telemonitoring in patients with heart failure: results of a multicentre randomized controlled trial. *Eur J Heart Fail* 2012;14(7):791-801. doi: 10.1093/eurjhf/hfs058.
21. Koehler F, Winkler S, Schieber M, et al. Telemedicine in heart failure: pre-specified and exploratory subgroup analyses from the TIM-HF trial. *Int J Cardiol* 2012;161(3):143-50. doi: 10.1016/j.ijcard.2011.09.007.
22. Koehler J, Stengel A, Hofmann T, et al. Telemonitoring in patients with chronic heart failure and moderate depressed symptoms: results of the Telemedical Interventional Monitoring in Heart Failure (TIM-HF) study. *Eur J Heart Fail* 2021;23(1):186-94. doi: 10.1002/ejhf.2025.
23. Galinier M, Roubille F, Berdague P, et al. Telemonitoring versus standard care in heart failure: a randomised multicentre trial. *Eur J Heart Fail* 2020;22(6):985-94. doi: 10.1002/ejhf.1906.
24. Krzesinski P, Jankowska EA, Siebert J, et al. Effects of an outpatient intervention comprising nurse-led non-invasive assessments, telemedicine support and remote cardiologists' decisions in patients with heart failure (AMULET study): a randomised controlled trial. *Eur J Heart Fail* 2022;24(3):565-77. doi: 10.1002/ejhf.2358.
25. Auener SL, Remers TEP, van Dulmen SA, et al. The Effect of Noninvasive Telemonitoring for Chronic Heart Failure on Health Care Utilization: Systematic Review. *J Med Internet Res* 2021;23(9):e26744. doi: 10.2196/26744.
26. Asselbergs FW, Baars F, Boyne JJJ, et al. Samenwerkingsafspraken en kwaliteitscriteria bij invoeren telebegeleiding bij hartfalen in Nederland 2016.
27. ClinicalTrials.gov. Identifier NCT05654961, REsponsible roLl-out of E-heAlth Through Systematic Evaluation - Heart Failure Study (RELEASE-HF) National Library of Medicine (US): Clinicaltrials.gov; 2022 [cited 2022 December 16]. Available from: https://classic.clinicaltrials.gov/ct2/show/NCT05654961?term=RELEASE-HF&draw=2&rank=1 accessed July 10 2023.
28. DCVA. Heart4Data [cited 2023 30 January]. Available from: https://dcvalliance.nl/heart4data accessed 30 January 2023.
29. NHR. Registratie [cited 2023 May 10]. Available from: https://nederlandsehartregistratie.nl/registratie/ accessed April 25 2022.
30. Timmermans MJ, Houterman S, Daeter ED, et al. Using real-world data to monitor and improve quality of care in coronary artery disease: results from the Netherlands Heart Registration. *Netherlands Heart Journal* 2022:1-9.
31. NHR. Handboek Nederlandse Hart Registratie | Hartfalen. 22.1.3 ed: Nederlandse Hart Registratie, 2022.
32. Aaronson NK, Muller M, Cohen PD, et al. Translation, validation, and norming of the Dutch language version of the SF-36 Health Survey in community and chronic disease populations. *J Clin Epidemiol* 1998;51(11):1055-68. doi: 10.1016/s0895-4356(98)00097-3.
33. Brazier JE, Roberts J. The estimation of a preference-based measure of health from the SF-12. *Med Care* 2004;42(9):851-9. doi: 10.1097/01.mlr.0000135827.18610.0d.
34. Bashshur R, Shannon G, Krupinski E, et al. The taxonomy of telemedicine. *Telemed J E Health* 2011;17(6):484-94. doi: 10.1089/tmj.2011.0103.
35. Edmunds M, Tuckson R, Lewis J, et al. An Emergent Research and Policy Framework for Telehealth. *EGEMS (Wash DC)* 2017;5(2):1303. doi: 10.13063/2327-9214.1303.
36. Tulu B, Chatterjee S, Maheshwari M. Telemedicine taxonomy: a classification tool. *Telemed J E Health* 2007;13(3):349-58. doi: 10.1089/tmj.2006.0055.

For peer review only - http://bmjopen.bmj.com/site/about/guidelines.xhtml

37. Houterman S, van Dullemen A, Versteegh M, et al. Data quality and auditing within the Netherlands Heart Registration: using the PCI registry as an example. *Neth Heart J* 2023 doi: 10.1007/s12471-022-01752-1.
38. NHR. Overzicht berekende velden - Nederlandse Hart Registratie [cited 2023 5 october]. Versie 23.1.0:[Available from: https://nhr.nl/wp-content/uploads/2022/11/NHR-Handboek-berekende-velden.pdf accessed 2 March 2023.
39. Nederland Z. Richtlijnen voor economische evaluatie [cited 2023 May 3]. Available from: https://www.zorginstituutnederland.nl/over-ons/werkwijzen-en-procedures/adviseren-over-en-verduidelijken-van-het-basispakket-aan-zorg/beoordeling-van-geneesmiddelen/richtlijnen-voor-economische-evaluatie2023.
40. Austin PC. Absolute risk reductions, relative risks, relative risk reductions, and numbers needed to treat can be obtained from a logistic regression model. *J Clin Epidemiol* 2010;63(1):2-6. doi: 10.1016/j.jclinepi.2008.11.004.
41. Greenland S. Model-based estimation of relative risks and other epidemiologic measures in studies of common outcomes and in case-control studies. *Am J Epidemiol* 2004;160(4):301-5. doi: 10.1093/aje/kwh221.
42. Localio AR, Margolis DJ, Berlin JA. Relative risks and confidence intervals were easily computed indirectly from multivariable logistic regression. *J Clin Epidemiol* 2007;60(9):874-82. doi: 10.1016/j.jclinepi.2006.12.001.
43. Chaudhry SI, Mattera JA, Curtis JP, et al. Telemonitoring in patients with heart failure. *N Engl J Med* 2010;363(24):2301-9. doi: 10.1056/NEJMoa1010029.
44. Cleland JG, Louis AA, Rigby AS, et al. Noninvasive home telemonitoring for patients with heart failure at high risk of recurrent admission and death: the Trans-European Network-Home-Care Management System (TEN-HMS) study. *J Am Coll Cardiol* 2005;45(10):1654-64. doi: 10.1016/j.jacc.2005.01.050.
45. Dendale P, De Keulenaer G, Troisfontaines P, et al. Effect of a telemonitoring-facilitated collaboration between general practitioner and heart failure clinic on mortality and rehospitalization rates in severe heart failure: the TEMA-HF 1 (TElemonitoring in the MAnagement of Heart Failure) study. *Eur J Heart Fail* 2012;14(3):333-40. doi: 10.1093/eurjhf/hfr144.
46. Koehler F, Koehler K, Deckwart O, et al. Efficacy of telemedical interventional management in patients with heart failure (TIM-HF2): a randomised, controlled, parallel-group, unmasked trial. *The Lancet* 2018;392(10152):1047-57.
47. Lynga P, Persson H, Hagg-Martinell A, et al. Weight monitoring in patients with severe heart failure (WISH). A randomized controlled trial. *Eur J Heart Fail* 2012;14(4):438-44. doi: 10.1093/eurjhf/hfs023.
48. Ong MK, Romano PS, Edgington S, et al. Effectiveness of Remote Patient Monitoring After Discharge of Hospitalized Patients With Heart Failure: The Better Effectiveness After Transition -- Heart Failure (BEAT-HF) Randomized Clinical Trial. *JAMA Intern Med* 2016;176(3):310-8. doi: 10.1001/jamainternmed.2015.7712.
49. Piotrowicz E, Pencina MJ, Opolski G, et al. Effects of a 9-Week Hybrid Comprehensive Telerehabilitation Program on Long-term Outcomes in Patients With Heart Failure: The Telerehabilitation in Heart Failure Patients (TELEREH-HF) Randomized Clinical Trial. *JAMA Cardiol* 2020;5(3):300-08. doi: 10.1001/jamacardio.2019.5006.
50. Szalewska D, Glowczynska R, Piotrowicz R, et al. An aetiology-based subanalysis of the Telerehabilitation in Heart Failure Patients (TELEREH-HF) trial. *ESC Heart Fail* 2021;8(2):1263-73. doi: 10.1002/ehf2.13189.
51. Pekmezaris R, Nouryan CN, Schwartz R, et al. A randomized controlled trial comparing telehealth self-management to standard outpatient management in underserved black and Hispanic patients living with heart failure. *Telemedicine and e-Health* 2019;25(10):917-25.

For peer review only - http://bmjopen.bmj.com/site/about/guidelines.xhtml

52. Brookes ST, Whitely E, Egger M, et al. Subgroup analyses in randomized trials: risks of subgroup-specific analyses; power and sample size for the interaction test. *J Clin Epidemiol* 2004;57(3):229-36. doi: 10.1016/j.jclinepi.2003.08.009.
53. Burke JF, Sussman JB, Kent DM, et al. Three simple rules to ensure reasonably credible subgroup analyses. *BMJ* 2015;351:h5651. doi: 10.1136/bmj.h5651.
54. Brugts JJ, Linssen GCM, Hoes AW, et al. Real-world heart failure management in 10,910 patients with chronic heart failure in the Netherlands : Design and rationale of the Chronic Heart failure ESC guideline-based Cardiology practice Quality project (CHECK-HF) registry. *Neth Heart J* 2018;26(5):272-79. doi: 10.1007/s12471-018-1103-7.
55. Cleland JGF, Tavazzi L, Daubert JC, et al. Cardiac Resynchronization Therapy Are Modern Myths Preventing Appropriate Use? *Journal of the American College of Cardiology* 2009;53(7):608-11. doi: 10.1016/j.jacc.2008.10.040.

For peer review only

Figure legend

Figure 1: Overview time plan RELEASE-HF study
HF: Heart Failure; RELEASE-HF: REsponsible roLl-out of E-heAlth through Systematic Evaluation – Heart Failure; NHR: Netherlands Heart Registration; EHR: Electronic Health Record.

Figure 2: Data sources and flow to set up the final RELEASE-HF dataset
RELEASE-HF: REsponsible roLl-out of E-heAlth through Systematic Evaluation – Heart Failure; H4D: Heart for Data Consortium; HF: Heart Failure.

Figure 3: This figure depicts how telemedicine characteristics are collected at the hospital level and at the individual patient level. It clarifies the source of the collected clinical content used for the subgroup analyses rather than the type of data source.

For peer review only

For peer review only - http://bmjopen.bmj.com/site/about/guidelines.xhtml

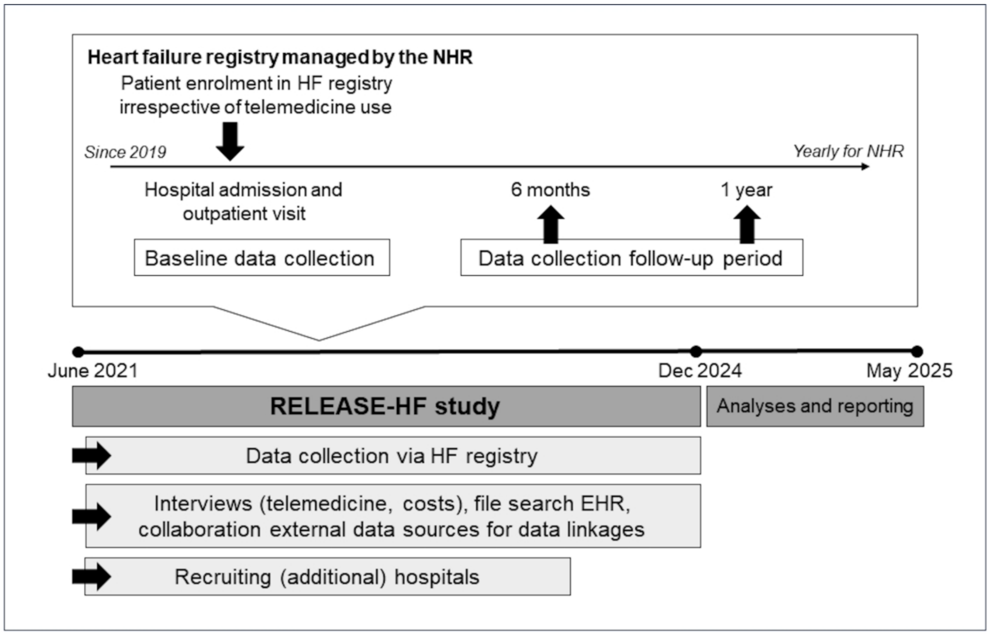

Figure 1 Overview time plan RELEASE-HF study. HF: Heart Failure; RELEASE-HF: REsponsible roLl-out of E-heAlth through Systematic Evaluation – Heart Failure; NHR: Netherlands Heart Registration; EHR: Electronic Health Record

256x161mm (300 x 300 DPI)

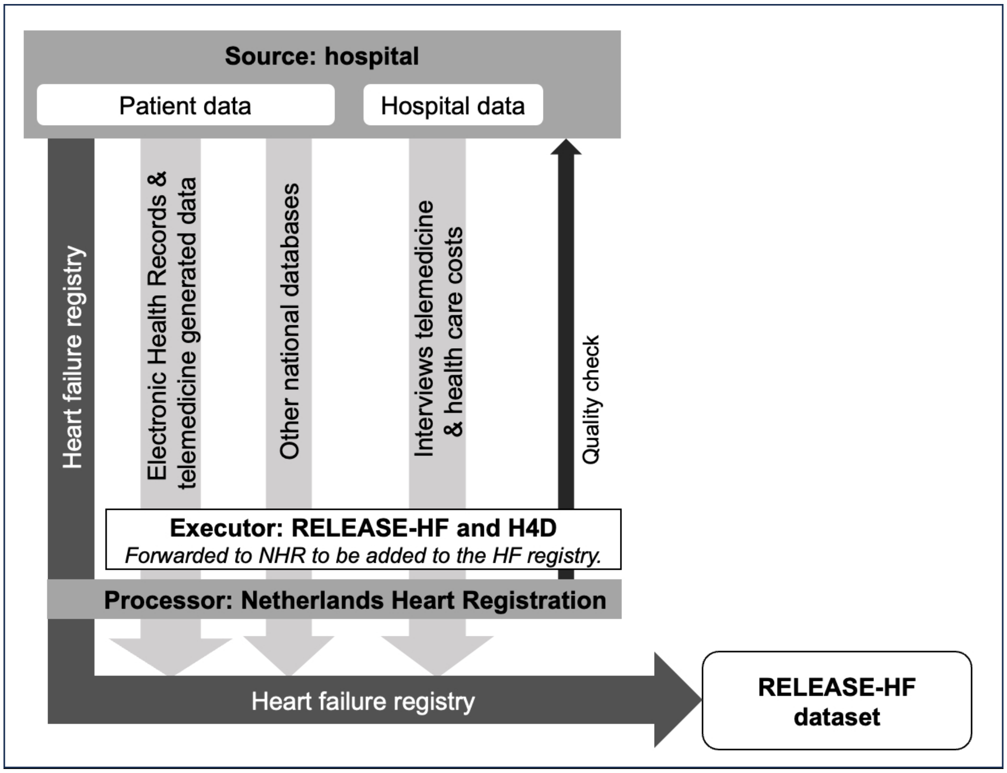

Figure 2 Data sources and flow to set up the final RELEASE-HF dataset. *RELEASE-HF: REsponsible roLl-out of E-heAlth through Systematic Evaluation – Heart Failure; H4D: Heart for Data Consortium; HF: Heart Failure.*

108x83mm (300 x 300 DPI)

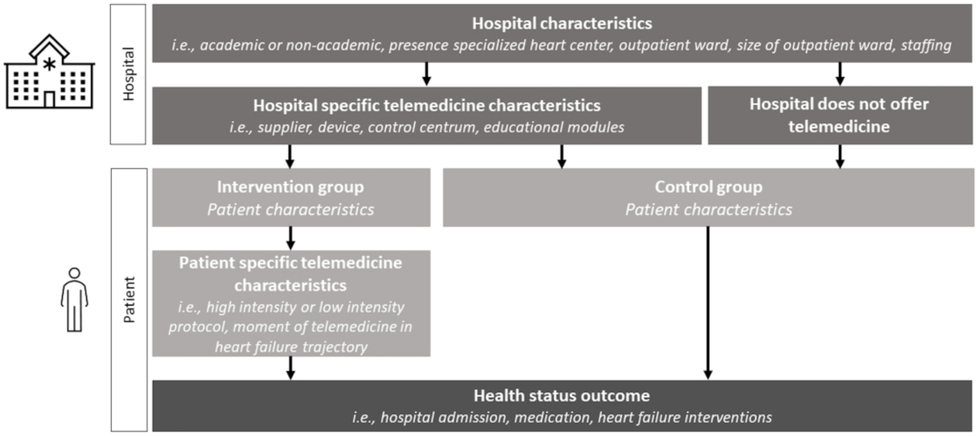

This figure depicts how telemedicine characteristics are collected at the hospital level and at the individual patient level. It clarifies the source of the collected clinical content used for the subgroup analyses rather than the type of data source.

338x190mm (300 x 300 DPI)

Supplementary material 1 Variables of the heart failure registry

NOTE: Based on the dynamic data dictionary version 22.1.4 September 9, 2022. Definitions of variables and included variables are subject to change, since data dictionary depends on the current HF guidelines and is compiled by a committee consisting of delegated cardiologists from Dutch hospitals.

	Variables	Answer possibilities	Baseline (T0)	6 months (T1)	12 months (T2)
Identifying variables	Diagnosis number *	Unique number set up by the hospital	X		
	Date of diagnosis *	Date (DD-MM-YYYY)	X		
	Patient number*	Identification number in the hospital	X		
	Maiden name*	Name	X		
	Surname*	Name	X		
	Date of birth$	Date (DD-MM-YYYY)	X		
	Sex	Male<br>Female	X		
	Zip code*	1000 AA – 9999 ZZ= the Netherlands<br>0000= Abroad<br>-1= Unknown	X		
Diagnostic variables	Setting of inclusion	10= Outpatient clinic<br>20= Hospital, novo patient<br>30= Hospital, acute/chronic HF patient<br>-1 = Unknown	X		
	Previous HF diagnosis	0= No<br>1= Yes<br>-1= Unknown	X		
	Date of previous diagnosis (if applicable)	Date (DD-MM-YYYY)	X		
	Location diagnosis HF	10= Not earlier diagnosed with HF<br>10= Primary care<br>20= Secondary care<br>30= Tertiary care<br>-1= Unknown	X		
	Type of HF	10= HFrEF<br>20= HFmrEF<br>30= HFpEF<br>-1= Unknown	X		
	Etiology HFrEF	0= No HFrEF<br>10= Ischemic cardiomyopathy<br>20= Non-ischemic, hypertensive cardiomyopathy<br>50= Non-ischemic, arrhythmia/tachycardia mediated<br>60= Non-ischemic, hypertrophic cardiomyopathy<br>70= Non-ischemic, dilated cardiomyopathy (eci)<br>80= Non-ischemic, inflammation/infection (i.e., myocarditis)<br>90= Nonischemic, restrictive cardiomyopathy and/or accumulation<br>111= Nonischemic, familiar cq. genetic cardiomyopathy with proven DNA mutation	X		

For peer review only

For peer review only - http://bmjopen.bmj.com/site/about/guidelines.xhtml

		120= Non-ischemic, pericardial<br>135= Non-ischemic, toxic cardiomyopathy due to alcohol/drugs<br>136= Non-ischemic, toxic cardiomyopathy due to chemotherapy/radiation<br>139= Non-ischemic, toxic cardiomyopathy due to other<br>140= Non-ischemic, eci or (as yet) undetermined<br>900= Other (i.e., valve disease, metabolic, peripartum)<br>-1= Unknown			
	Etiology HFpEF	0= No HFpEF<br>10= Secondary HFpEF<br>20= Isolated hypertensive heart disease<br>30= Genetic HCM<br>40= Amyloidosis<br>50= Sarcoidosis<br>60= Hemochromatosis<br>70= Immune/inflammatory<br>80= Aortic valve stenosis<br>90= Pericardial diseases<br>900= Other<br>-1= Unknown	**X**		
**Patient characteristics**	***Identifying variables***				
	Follow-up moment	0 = T0<br>6 = T1<br>12 = T2<br>24 = T3<br>36 = T4<br>48 = T5	**X**	**X**	**X**
	Comorbidities				
	Hypertension	0= No<br>1= Yes<br>-1= Unknown	**X**	**X**	**X**
	Cardiovascular accident	0= No<br>1= Yes<br>-1= Unknown	**X**	**X**	**X**
	Extracardiac arterial vascular pathology	0= No<br>1= Yes<br>-1= Unknown	**X**	**X**	**X**
	Chronic respiratory disease	0= No<br>1= Yes<br>-1= Unknown	**X**	**X**	**X**
	Obstructive Sleep Apnea Syndrome	0= No<br>1= Yes<br>-1= Unknown	**X**	**X**	**X**
	Diabetes Mellitus	0= No<br>1= Diabetes, treatment unknown<br>2= Diabetes, no treatment<br>10= Diabetes, diet<br>20= Diabetes, oral medication<br>30= Diabetes, insulin<br>90= Diabetes, other<br>-1= Unknown	**X**	**X**	**X**
	Thyroid disorder	0= No<br>10= Yes, hypothyroidism<br>20= Yes, hyperthyroidism	**X**	**X**	**X**

For peer review only

		-1= Unknown			
	Malignancy	0= No<br>10= Yes, in the past<br>20= Yes, currently<br>-1= Unknown	X	X	X
	Measurements				
	Length	Measurement in centimeters	X	X	X
	Weight	Measurement in kilogram	X	X	X
	Systolic blood pressure	Measurement in mmHg	X	X	X
	Diastolic blood pressure	Measurement in mmHg	X	X	X
	Heart rhythm	10= Sinus rhythm<br>20= Atrium fibrillation<br>30= Atrial pacing<br>90= Other<br>-1= Unknown	X	X	X
	Heartrate	Measurement in beats per minute	X	X	X
	Atrial heart rhythm disorder	0= No<br>1= Yes<br>-1= Unknown	X	X	X
	Left bundle branch block	0= No<br>10= Yes, own heart's electrical conduction<br>20= Yes, pacing rhythm<br>-1= Unknown	X	X	X
	QRS complex duration	Measurement in milliseconds	X	X	X
	Left ventricle ejection fraction, NYHA classification and laboratory parameters				
	Left ventricle ejection fraction	Measurement in percentages	X	X	X
	NYHA class	1= Class I<br>2= Class II<br>3= Class III<br>4= Class IV<br>-1= Unknown	X	X	X
	NT-proBNP	1 t/m 8000= Value in pmol/l<br>0= No NT-proBNP measurement<br>-1= Unknown	X	X	X
	Date measurement NT-proBNP[$]	Date (DD-MM-YYYY)	X	X	X
	Serum creatinine	Value in µmol/l	X	X	X
	Iron deficiency	1= No<br>2= Yes, absolute ferritin < 100 µg/L<br>3= Yes, relative ferritin 100-300 µg/L with transferrin saturation < 20%<br>-1= Unknown	X	X	X
	Anemia	0= No<br>1= Yes<br>-1= Unknown	X	X	X
**Medication**	***Medication with target dose***				
	Type ACE inhibitor	0= No ACE inhibitor prescribed at Tn<br>10= Captopril (td in ESC 2021 = 150 mg)<br>20= Enalapril (td in ESC 2021= 20-40 mg)<br>3-= Lisinopril (td in ESC 2021 = 20-35 mg)<br>40= Ramipril (td in ESC 2021 = 10 mg)<br>50= Perindopril (td in ESC 2021 = 8 mg)	(X)	X	X

For peer review only

For peer review only - http://bmjopen.bmj.com/site/about/guidelines.xhtml

		60= Quinapril (td in ESC 2021 = 20 mg)<br>70= Fosinopril (td in ESC 2021 = 20-40 mg)<br>90= Other<br>-1= Unknown			
	Target dose ACE inhibitor	0= No ACE inhibitor prescribed at Tn<br>1= No, target dose in ESC 2021 not reached due to contraindication or otherwise<br>2= No, target dose in ESC 2021 not reached due to titration phase<br>10= Yes, target dose in ESC 2021 reached or maximally tolerable for this patient<br>-1= Unknown	**(X)**	**X**	**X**
	Type ARB	0= No ARB prescribed at Tn<br>10= Candesartan (td in ESC 2021 = 32 mg)<br>20= Losartan (td in ESC 2021 = 150 mg)<br>30= Valsartan (td in ESC 2021 = 320 mg)<br>40= Telmisartan (td in ESC 2021 = 80 mg)<br>50= Irbesartan (td in ESC 2021 = 300 mg)<br>90= Other<br>-1= Unknown	**(X)**	**X**	**X**
	Target dose ARB	0= No ARB prescribed at Tn<br>1= No, target dose in ESC 2021 not reached due to contraindication or otherwise<br>2= No, target dose in ESC 2021 not reached due to titration phase<br>10= Yes, target dose in ESC 2021 reached or maximally tolerable for this patient<br>-1= Unknown	**(X)**	**X**	**X**
	ARNI	0= No<br>1= Yes (td in ESC 2021 = 194-206 mg)<br>-1= Unknown	**(X)**	**X**	**X**
	Target dose ARNI	0= No ARNI prescribed at Tn<br>1= No, target dose in ESC 2021 not reached due to contraindication or otherwise<br>2= No, target dose in ESC 2021 not reached due to titration phase<br>10= Yes, target dose in ESC 2021 reached or maximally tolerable for this patient<br>-1= Unknown	**(X)**	**X**	**X**
	Type beta-blocker	0 = No beta blocker prescribed at Tn<br>10= Bisoprolol (td in ESC 2021 = 10 mg)<br>20= Metoprolol (td in ESC 2021 = 200 mg)<br>30= Carvedilol (td in ESC 2021 = 50 mg)	**(X)**	**X**	**X**

For peer review only - http://bmjopen.bmj.com/site/about/guidelines.xhtml

		40= Nebivolol (td in ESC 2021 = 10 mg)<br>50= Atenolol (td in ESC 2021 = 100 mg)<br>60= Pindolol (td in ESC 2021 = 20 mg)<br>70= Propranolol (td in ESC 2021 = 160 mg)<br>90= Other<br>-1= Unknown			
	Target dose beta-blocker	0= No beta blocker prescribed at Tn<br>1= No, target dose in ESC 2021 not reached due to contraindication or otherwise<br>2= No, target dose in ESC 2021 not reached due to titration phase<br>10= Yes, target dose in ESC 2021 reached or maximally tolerable for this patient<br>-1= Unknown	**(X)**	**X**	**X**
	Type MRA	0= No MRA prescribed at Tn<br>10= Eplerenone (td in ESC 2021= 50 mg)<br>20= Spironolactone (td in ESC 2021= 50 mg)<br>90= Other<br>-1= Unknown	**(X)**	**X**	**X**
	Target dose MRA	0= No MRA prescribed at Tn<br>1= No, target dose in ESC 2021 not reached due to contraindication or otherwise<br>2= No, target dose in ESC 2021 not reached due to titration phase<br>10= Yes, target dose in ESC 2021 reached or maximally tolerable for this patient<br>-1= Unknown	**(X)**	**X**	**X**
	Type SGLT2 inhibitor	0= No SGLT2 inhibitor prescribed at Tn<br>10= Canagliflozin (td in ESC 2021 = 10 mg)<br>20= Dapagliflozin (td in ESC 2021 = 10 mg)<br>30= Empagliflozin (td in ESC 2021 = 10 mg)<br>90= Other<br>-1= Unknown	**(X)**	**X**	**X**
	Target dose SGLT2 inhibitor	0= No SGLT2 inhibitor prescribed at Tn<br>1= No, target dose in ESC 2021 not reached due to contraindication or otherwise<br>2= No, target dose in ESC 2021 not reached due to titration phase<br>10= Yes, target dose in ESC 2021 reached or maximally tolerable for this patient<br>-1= Unknown	**(X)**	**X**	**X**
	Ivabradine	0= No<br>1= Yes	**(X)**	**X**	**X**

For peer review only - http://bmjopen.bmj.com/site/about/guidelines.xhtml

		-1= Unknown			
	Target dose Ivabradine	0= No ivabradine prescribed at Tn<br>1= No, target dose in ESC 2021 not reached due to contraindication or otherwise<br>2= No, target dose in ESC 2021 not reached due to titration phase<br>10= Yes, target dose in ESC 2021 reached or maximally tolerable for this patient<br>-1= Unknown	**(X)**	**X**	**X**
	Additional medication				
	Diuretic	0= No<br>1= Yes (target dose in ESC 2021 = 15mg)<br>-1= Unknown	**(X)**	**X**	**X**
	Vericiguat	0= No<br>1= Yes<br>-1= Unknown	**(X)**	**X**	**X**
	Digoxin	0= No<br>1= Yes<br>-1= Unknown	**(X)**	**X**	**X**
	Amiodarone	0= No<br>1= Yes<br>-1= Unknown	**(X)**	**X**	**X**
	Sotalol	0= No<br>1= Yes<br>-1= Unknown	**(X)**	**X**	**X**
	Hydralazine / Isosorbide dinitrate	0= No<br>1= Yes<br>-1= Unknown	**(X)**	**X**	**X**
**Outcome variables**	***Cardiac intervention and implantable***				
	Pacemaker – VVI	0= No<br>1= Yes<br>-1= Unknown	**(X)**	**X**	**X**
	Pacemaker – VVI date$	Date (DD-MM-YYYY)	**(X)**	**X**	**X**
	Pacemaker – DDD	0= No<br>1= Yes<br>-1= Unknown	**(X)**	**X**	**X**
	Pacemaker – DDD date$	Date (DD-MM-YYYY)	**(X)**	**X**	**X**
	CRT-P	0= No<br>1= Yes<br>-1= Unknown	**(X)**	**X**	**X**
	CRT-P date$	Date (DD-MM-YYYY)	**(X)**	**X**	**X**
	CRT-D	0= No<br>1= No, because of medical condition<br>2= No, because of patients' wish<br>10= Yes<br>-1= Unknown	**(X)**	**X**	**X**
	CRT-D date$	Date (DD-MM-YYYY)	**(X)**	**X**	**X**
	ICD – VVI	0= No<br>1= No, because of medical condition<br>2= No, because of patients' wish<br>10= Yes<br>-1= Unknown	**(X)**	**X**	**X**
	ICD – VVI date$	Date (DD-MM-YYYY)	**(X)**	**X**	**X**
	ICD – DDD	0= No<br>1= No, because of medical condition<br>2= No, because of patients' wish	**(X)**	**X**	**X**

For peer review only

For peer review only - http://bmjopen.bmj.com/site/about/guidelines.xhtml

		10= Yes<br>-1= Unknown			
	ICD – DDD date$	Date (DD-MM-YYYY)	(X)	X	X
	PCI	0= No<br>1= Yes<br>-1= Unknown	(X)	X	X
	PCI date$	Date (DD-MM-YYYY)	(X)	X	X
	CABG	0= No<br>1= Yes<br>-1= Unknown	(X)	X	X
	CABG date$	Date (DD-MM-YYYY)	(X)	X	X
	LVAD	0= No<br>1= Yes<br>-1= Unknown	(X)	X	X
	LVAD date$	Date (DD-MM-YYYY)	(X)	X	X
	Heart transplantation	0= No<br>1= Yes<br>-1= Unknown	(X)	X	X
	Heart transplantation date$	Date (DD-MM-YYYY)	(X)	X	X
	Heart revalidation	0= No<br>1= Yes<br>-1= Unknown	(X)	X	X
	Telemonitoring	0= No telemonitoring<br>10= Telemonitoring by telephone<br>20= Telemonitoring, non-invasive based on traditional parameters (e.g., blood pressure, ECG)<br>30= Telemonitoring, using ICD based on HF parameters<br>40= Telemonitoring, invasive by sensors in the blood stream or heart<br>-1= Unknown	(X)	X	X
	Patient status and quality of life				
	Mortality	0= Alive<br>1= Deceased<br>-1= Unknown		X	X
	Date mortality$	Date (DD-MM-YYYY)		X	X
	Number of hospitalizations	Number		X	X
	Date hospitalization$	Date (DD-MM-YYYY)		X	X
	Number of hospitalization days	Number		X	X
	Location follow-up	0= No follow-up, because of mortality<br>10= Patient stays in secondary care, outpatient HF clinic<br>20= Patient stays in tertiary care, outpatient HF clinic<br>30= Patient transferred to primary care<br>40= Patient transferred to the cardiology outpatient clinic<br>90= Other<br>-1= Unknown		X	X
	Type quality of life questionnaire	0= Not measured<br>10= SF12-2<br>20= SF36-2<br>30= SF36-1<br>-1= Unknown	X	X	X
	Quality of life	Coding depends on questionnaire	X	X	X

*Variables are collected in the Heart failure registry, but the data is not distributed and available for research.

For peer review only - http://bmjopen.bmj.com/site/about/guidelines.xhtml

$All variables which consist of a date will be transformed to number of days since diagnosed with HF. The actual date is not available in research.

HF: Heart Failure; HFrEF: Heart Failure with reduced Ejection Fraction; HFmrEF: Heart Failure with mildly reduced Ejection Fraction; HFpEF: Heart failure with preserved Ejection Fraction; NYHA: New York Heart Association; eci: e causa ignota (unknown cause); NT-proBNP: N-Terminal pro B-type Natriuretic Peptide; ACE: Angiotensin Converting Enzyme; td: target dose; ARB: Angiotensin Receptor Blocker; ARNI: Angiotensin Receptor-Neprilysin Inhibitor; MRA: Mineralocorticoid Receptor Antagonists; SGLT2: Sodium-glucose co-transporter-2; VVI: Ventricular pacing; DDD: dual-chamber antibradycardia pacing; CRT-P: Cardiac Resynchronization Therapy with a Pacemaker; CRT-D: Cardiac Resynchronization Therapy with a pacemaker and an ICD; ICD: Implantable Cardioverter-Defibrillator; PCI: Percutaneous Coronary Intervention; CABG: Coronary Artery Bypass Graft; LVAD: Left Ventricular Assist Device; ECG: Electro Cardio Gram; SF12/SF36: Short Form Health Survey with 12 questions or 36 questions.

For peer review only

Supplementary material 2 Interview guide telemedicine

Introduction

What do we mean by telemedicine?

With telemedicine, we mean the provision of care at a distance. The healthcare provider and the patient are not physically present with each other at that moment. Telemedicine can consist of telemonitoring or tele-education (according to the Collaboration Agreement and Quality Criteria for Telemedicine).

- *Telemonitoring* involves monitoring heart failure symptoms and relevant parameters used to support patients and healthcare providers in the treatment of heart failure.
- *Tele-education* is a service that allows the patient/user to receive remote education. Receiving this education can be accessed through a variety of applications and devices.

For peer review only

Background Information: Use of Telemedicine

1) Does your hospital offer telemedicine to outpatient heart failure patients?
   - Yes, we offer telemedicine.
     i. Why do you offer telemedicine?
        - *Possibilities: monitoring, education, self-care/self-management, consultation, cost reduction, reduction in hospital admissions, improvement in quality of life, reducing workload.*
   - No, we do not offer telemedicine.
     i. Why don't you offer telemedicine? (How strong is the influence of [x] in this?)
        - *Possibilities: no patient/clinician demand, costs, evidence, user-unfriendly technology, no connection to EHR, HF care pathway is different, time, shortage of healthcare professionals, significant investment compared to the amount of patients, legal/privacy concerns, others.*

     ii. Have you considered using telemedicine for HF patients?
        - What prompted you to consider it but not proceed?

     iii. Which professionals were involved in this decision?
   - No, but we plan to start telemedicine.
     i. Why are you planning to start telemedicine?
        - *Possibilities: patient/clinician desire, guidelines, COVID-19, funding, others.*

     ii. To what extent did COVID-19 influence this decision?

     iii. Which professionals are involved in this decision?
   - No, we stopped telemedicine.
     i. Why did you stop telemedicine?
        - *Possibilities: no patient demand, didn't meet expectations, more time, moderate positive effects, cost, lack of staff, others.*

     ii. Who were involved in this decision? (Professionals)

Users of Telemedicine

From this point on, the conversation continues with the focus on the performers of telemedicine from the perspective of healthcare professional.

2) Which system do you use for telemedicine?
   - Luscii
   - Sananet/SanaCoach
   - Motiva (Philips)
   - 24Care
   - Hartwacht
   - CardioMEMS
   - Empower
   - Other, namely: ...

3) Why do you use telemedicine for heart failure patients?
   - Why is telemedicine used for [goal X]?
     i. *Functionalities: monitoring, education, self-care/self-management, consultation.*
     ii. *Endpoints: cost reduction, reduction in hospital admissions, improvement in quality of life, reducing workload for healthcare professionals.*
     iii. *Other, namely: ...*

Offering Telemedicine

4) Which patients visit the heart failure outpatient clinic?
   i. *Severity, age, duration of illness.*

5) Which heart failure patients at your outpatient clinic are eligible for telemedicine?
   i. What factors are considered in the decision to use telemedicine?
   ii. *Telemedicine guideline: Chronic HF, recently diagnosed, readmission due to exacerbation, anxious/uncertain/depressive patients, assistance in early detection of deterioration.*
   iii. *Characteristics: caregiver network, patient/donor digital skills, language proficiency, patient mobility, internet connection, distance to hospital, other.*
   - Does the severity of heart failure play a role in initiating telemedicine?
     - Yes
     - No
   - At which NYHA class is telemedicine used?
     - NYHA 1
     - NYHA 2
     - NYHA 3
     - NYHA 4
   - Why this NYHA class?

6) Which heart failure patients at your outpatient clinic are not eligible for telemedicine?
   - Why are these patients not eligible?
     i. *Telemedicine guideline: Chronic HF, recently diagnosed, readmission due to exacerbation, anxious/uncertain/depressive patients, assistance in early detection of deterioration.*
     ii. *Characteristics: caregiver network, patient/donor digital skills, language proficiency, patient mobility, internet connection, distance to hospital, other.*

7) When is telemedicine for the first time considered for heart failure patients? (multiple answers possible)
   - For every newly diagnosed heart failure patient
   - After a hospital admission

For peer review only - http://bmjopen.bmj.com/site/about/guidelines.xhtml

- For patients attending the heart failure outpatient clinic (regardless of previous hospital admission)
- After an exacerbation (patient is already diagnosed with heart failure)
  - i. When is telemedicine offered after an exacerbation?
    - After 1 exacerbation
    - After ... exacerbations
    - If the patient has more than ... exacerbations
  - i. Does the time period in which the patient has an exacerbation play a role in offering telemedicine?
    - Yes, when ... exacerbations in ... weeks
    - No
    - In the titration phase
  - i. Is telemedicine offered during the titration phase?
    - Yes, every time there is a change in medication
    - Yes, only when the patient is newly diagnosed with the disease
    - Sometimes, depending on the situation, ease of titration, patient's distance, pandemic, etc.
    - No
  - i. For which medication?
- In the stable phase of HF
  - i. Is telemedicine offered in every stable phase of heart failure?
    - Yes
    - No
- Others, namely: ...

8) Which healthcare professionals are involved in determining whether or not to use telemedicine for the patient? (multiple answers possible)
   - Cardiologist
   - Heart failure nurse
   - Nurse Specialist / Physician Assistant
   - General Practitioner
   - Home care nurse
   - General practice nurse
   - Other, namely: ...

Stopping/On-Off principle of telemedicine

9) Is telemedicine offered multiple times in a heart failure care trajectory?
   - Yes
     - i. How is this offered?
     - ii. For which patients?
     - iii. What determines the on/off/on/off principle?
     - iv. Is the equipment removed?
   - No

10) When is telemedicine stopped?
    - *i. Patient request, death, healthcare professional's assessment, disease severity, treatment phase, established time period, other*
    - Does NYHA class play a role in this?

For peer review only - http://bmjopen.bmj.com/site/about/guidelines.xhtml

Modules of the Telemedicine System

11) On which device can the patient receive telemedicine? *(multiple answers possible)*
    - Application on a mobile phone (smartphone)
    - Computer
    - Television
    - Watch (smartwatch)
    - iPad
    - Other, namely: ...

Telemonitoring

12) Does your telemedicine system include telemonitoring (e.g., vital signs, nutrition, medication, heart failure symptoms)?
    - Yes
        i. What is the main reason for using telemonitoring?
            - *Early detection of exacerbation, titration, longitudinal follow-up, self-management, other*
    - No

13) What type of telemonitoring do you use?
    - Non-invasive
    - Invasive
    - Non-invasive and invasive

14) Which measurements are monitored via telemonitoring?
    - Weight
    - Blood pressure
    - Heart rate
    - Heart rhythm (irregular, regular)
    - Temperature
    - Saturation
    - Intake (e.g., in the context of sodium restriction)
    - Fluid intake
    - Medication
    - Heart failure-related symptoms
        i. What symptoms do you monitor?
            - *Fatigue, decreased appetite, shortness of breath, swollen legs and ankles, cold hands and feet, a full feeling in the upper abdomen, palpitations, nocturia, constipation, tickling cough, restless sleep, memory and concentration problems, dizziness*
            - *Other, namely: ....*
    - Other, namely: ....

15) What equipment has the patient received for telemonitoring? *(multiple answers possible)*
    - No equipment
    - Patient uses their own equipment
        i. What equipment?
    - Weight scale
    - Bluetooth-enabled weight scale
    - Blood pressure monitor
    - Bluetooth-enabled blood pressure monitor
    - Pulse oximeter
    - Bluetooth-enabled pulse oximeter
    - ECG device

For peer review only - http://bmjopen.bmj.com/site/about/guidelines.xhtml

For peer review only

- CardioMEMS
- Other, namely: ....

16) Can a patient transmit monitored (vital) signs via the telemonitoring system to the healthcare provider?
    - Yes
        i. When can the patient transmit these monitored (vital) signs? (time of day)
            - *Possible options: 24/7, office hours, weekend, evening, night, set times, other, namely*
        ii. How does the patient transmit the monitored (vital) signs to the healthcare professional? (communication)
            - *Possible options: letter, phone call, video call, chat, SMS, email, via applications, other*
    - No

17) Does the healthcare professional receive a notification when monitored (vital) signs are entered into the telemonitoring system?
    - Yes
        i. When does the healthcare professional receive a notification?
            - *Possible options: always, when threshold values are exceeded, other*
        i. Where does the healthcare professional receive the notification?
            - *Possible options: Electronic Patient Record (EPD), standalone applications, email inbox, other*
        ii. Does the healthcare professional always receive the notification in the same manner, or does severity of exceeding the threshold play a role?
            - Yes
            - No
                i. What is the difference?
        iii. Which healthcare professional receives these messages?
            - *Possible options: cardiologist, heart failure nurse, nurse practitioner, physician assistant, general practitioner (GP), practice nurse, home care worker, other*
        iv. Are there erroneous notifications?
            - *How do you handle them?*
    - No
        v. How often does the healthcare professional evaluate the monitored vital signs, questionnaires, outcomes filled in by the patient in the telemonitoring system?
            a. Per week: ....
            b. Per day: ....
            c. Per patient: ....

18) Can a healthcare professional respond to the received monitored (vital) signs?
    - Yes
        i. Which healthcare professional responds to the received results?
            - *Possible options: cardiologist, heart failure nurse, nurse practitioner, physician assistant, general practitioner (GP), practice nurse, home care worker, other*
        ii. When can the healthcare professional respond to these monitored (vital) signs? (time of the day)
            - *Possible options: 24/7, office hours, weekend, evening, night, set times, other*
        iii. How does the healthcare professional respond to the monitored (vital) signs?
            - *Possible options: letter, phone call, video call, chat, SMS, email, via applications, other*
        iv. Does the patient receive a notification if the healthcare professional has sent a message to the patient?
    - No

For peer review only

19) Can thresholds be set for the monitored (vital) signs in the telemonitoring system?
   - No
   - Yes, general thresholds (not patient-specific)
      i. What are the thresholds based on? / How are the thresholds formulated?
   - Yes, patient-specific thresholds
      i. Can the thresholds be adjusted per patient, per situation, per moment?
         - Yes
         - No
      i. What are the thresholds based on? / How are the thresholds formulated?

20) Is there always contact between the patient and the healthcare professional for the monitored (vital) signs transmitted via the telemonitoring system?
   - Yes
      i. Is the contact direct or indirect (store-and-forward) between the patient and the healthcare professional?
         - *Direct: The patient and healthcare professional are using the system simultaneously, allowing for direct contact via one of the techniques provided by the telemonitoring system.*
         - *Indirect: The patient and healthcare professional have asynchronous contact with each other. The patient/healthcare professional sends their information to the recipient. The recipient reads the message later and responds to the sender.*
         - *Combination of direct and indirect contact*
   - No
      i. When is there contact between the patient and healthcare professional?

21) What actions does the healthcare professional take with the received monitored (vital) signs?
      i. How do you initiate these actions?
         - *Possible options: letter, phone call, video call, chat, SMS, email, via applications, other*
      ii. Does the telemonitoring system provide automatically generated advice to the patient when they enter data in a module in the telemonitoring system?
         - *Yes*
         - *No*

22) What is your experience with the telemonitoring provided by the telemonitoring system?

Education and self-care

23) Does your telemedicine system include the modules education and self-care?
   - Yes
      i. Does the education module need to be actively activated for each patient by the healthcare professional? (Note: Can the telemonitoring system also exist without the education module?)
         - Yes
         - No
      ii. Which topics are included in the education?
         - General medical information about heart failure
         - Information about treatment
         - Prevention of symptoms/exacerbations
         - Medication use
         - Fluid and dietary management
         - Physical activity
         - Other, namely: ...

For peer review only - http://bmjopen.bmj.com/site/about/guidelines.xhtml

iii. Do you actively refer the patient to the education module in the telemedicine system? (Note: Do you discuss this topic in your consultation?)
   - Yes
   - No
i. Is the education module the same for every patient?
ii. Which self-care components are included in the telemedicine system?
   - Lifestyle interventions,
     - Which behaviors/lifestyle interventions are included?
       - *Possible answers: exercise, nutrition, stress management*
   - Psychological well-being: information, knowledge, tools
   - Patient's medication overview
   - Medication explanation (medication glossary)
   - Medication intake reminder as per prescription
   - Referral to external sources and links related to heart failure
   - Knowledge quiz/ability to test knowledge about heart failure
   - Monitoring
   - Self-detection and the ability to anticipate/adjust medication (e.g., detecting weight gain through monitoring and then taking and recording medication [diuretics])
   - Other, namely: ...

- No

24) To what extent does the patient make decisions based on the telemedicine system?
   - Are efforts made that the patient take independent action / anticipate in response to measured (vital) signs?

25) To what extent does the telemedicine system promote the patient's self-care? / What is the effect of this module on the patient's self-care (skills)?

26) What are your experiences with the built-in self-care and education modules in the telemedicine system?

Heart Failure Community

27) Can the patient use the telemedicine system to get in touch with peers (for example, a heart failure community)?
   - Yes, always
   - Yes, but this module needs to be activated
     i. How is the heart failure community organized?
        - *Local (hospital)*
        - *Regional*
        - *National*
   - No, this is not possible

For peer review only

Analog Telemonitoring (via telephone)

28) Do you have contact with heart failure patients by phone (hospital to patient)?
    - Yes
        1. Who initiates contact with these patients by phone?
            - *Possible options: cardiologist, heart failure nurse, nurse practitioner (NP), physician assistant (PA), general practitioner (GP), general practice nurse (POH), home care nurse, other*
        2. When do you have contact with the patient by phone?
        3. What is the purpose of contact by phone?
        4. How often do you have contact with the patient by phone?
        5. Which patients receive telephone guidance?
        6. Why these patients?
    - No

29) Can the patient initiate contact by phone with the heart failure outpatient clinic (patient to hospital)?
    - Yes
        1. When can/may the patient initiate this type of contact?
        2. Who does the patient have telephone contact with?
            - *Possible options: cardiologist, heart failure nurse, nurse practitioner (NP), physician assistant (PA), general practitioner (GP), general practice nurse (POH), home care nurse, other*
    - No

30) Can a heart failure patient or healthcare professional seek contact in any other way?
    - Through which channel does this contact occur?
        - *Possible answer: patient portal*
    - When does this contact occur via this channel?

31) What is your experience with telemonitoring by phone?

External Influences on the Use of Telemonitoring
Evaluation

32) Is the telemedicine system evaluated (experiences, application in HF care, impact on objectives) among healthcare professionals?
    - Yes
    - No

33) What is the influence of telemedicine on the patient population that visits the (regular) heart failure outpatient clinic?
    - What are the characteristics of these patients?

34) Is the telemedicine system evaluated (experiences, application in their disease, usage) between the healthcare professional and the patient?
    - Yes
    - No
        1. Are expectations discussed with the patient regarding the use of telemedicine?
        2. What feedback do you receive?
        3. What is the experience of telemedicine from the patients' perspective?

For peer review only - http://bmjopen.bmj.com/site/about/guidelines.xhtml

Experience

35) Do you believe that telemedicine in your hospital has led to efficiency?
    - Yes
        i. On which outcomes?
        ii. Has this been objectively assessed?
    - No
        i. What makes you say that?

36) Would you recommend the telemedicine system you use?
    - Why?

37) What would you like to change/improve about your telemedicine system?

38) If you could choose a telemedicine system again, what would it look like?

COVID-19

39) Does COVID-19 have an impact on the use of telemedicine in your heart failure outpatient clinic? (multiple answers possible)
    - Yes, telemedicine is used for more patients as an intervention.
    - Yes, telemedicine is used less frequently for patients as an intervention.
    - Yes, telemedicine is used differently by patients who were already using this intervention before COVID-19.
        i. Which devices of the telemedicine system are used differently since COVID-19?
            - *Possible answers: phone calls, video calls, chat, email, SMS, chatbot, other.*
        i. Which modules of the telemedicine system are used differently since COVID-19?
            - *Possible answers: telemonitoring, education, self-care/self-management, heart failure community, other.*
        i. What do you think of this development?
    - No, there is no difference in the use of telemedicine before, during, and in the current COVID-19 period.

Time Investment in telemedicine vs. No telemedicine in Heart Failure Care (HTA)

40) How much time do you, on average, spend on an appointment at a heart failure outpatient clinic without telemedicine?
    ... minutes / hours

41) How much time do you, on average, spend on an appointment at a heart failure outpatient clinic with telemedicine?
    ... minutes / hours

42) How much time does it take to explain telemedicine to a heart failure patient (for the initial setup)?
    ... minutes / hours

43) How much time do you, on average, spend per week on providing telemedicine? (this includes monitoring digital statuses transmitted through the telemonitoring system, contacting the patient as needed, evaluating measurements, etc.)
    ... minutes / hours

For peer review only - http://bmjopen.bmj.com/site/about/guidelines.xhtml
